# Copper-Catalyzed Annulation–Cyanotrifluoromethylation of 1,6-Enynes Toward 1-Indanones via a Radical Process

**DOI:** 10.3389/fchem.2020.00234

**Published:** 2020-04-17

**Authors:** Tian-Shu Zhang, Wen-Juan Hao, Pei-Jun Cai, Guigen Li, Shu-Jiang Tu, Bo Jiang

**Affiliations:** ^1^School of Chemical Engineering & Technology, China University of Mining and Technology, Xuzhou, China; ^2^Jiangsu Key Laboratory of Green Synthetic Chemistry for Functional Materials, School of Chemistry & Materials Science, Jiangsu Normal University, Xuzhou, China; ^3^Collaborative Innovation Center of Chemistry for Life Sciences, Institute of Chemistry and BioMedical Sciences, Nanjing University, Nanjing, China; ^4^Department of Chemistry and Biochemistry, Texas Tech University, Lubbock, TX, United States

**Keywords:** Cu(II) catalysis, annulation–difunctionalization, cyanotrifluoromethylation, 1, 6-enynes, 1-indanones

## Abstract

A new Cu(II)-catalyzed annulation–cyanotrifluoromethylation of 1,6-enynes with Togni's reagent and trimethylsilyl cyanide (TMSCN) has been established, enabling the direct construction of trifluoromethylated 1-indanones with an all-carbon quaternary center in good yields. This reaction was performed by using low-cost Cu(OTf)_2_ as the catalyst and Togni's reagent as both the radical initiator and a CF_3_ source, providing an efficient protocol for building up an 1-indanone framework with wide functional group compatibility. The reaction mechanism was proposed through a radical triggered addition/5-*exo*-*dig* cyclization/oxidation/nucleophilic cascade.

**Graphical Abstract:**
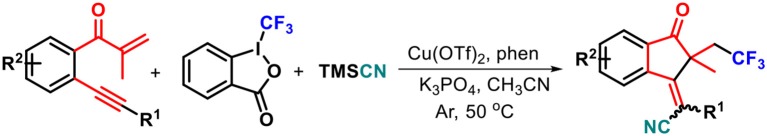
A new Cu(II)-catalyzed annulation-cyanotrifluoromethylation of 1,6-enynes with Togni's reagent and TMSCN has been established, enabling the direct construction of trifluoromethylated 1-indanones with an all-carbon quaternary center in good yields. This reaction was performed by using low-cost Cu(OTf)_2_ as the catalyst and Togni's reagent as both the radical initiator and a CF3 source, providing an efficieA new Cu(II)-catalyzed annulation-cyanotrifluoromethylation of 1,6-enynes with Togni's reagent and TMSCN has been established, enabling the direct construction of trifluoromethylated 1-indanones with an all-carbon quaternary center in good yields. This reaction was performed by using low-cost Cu(OTf)_2_ as the catalyst and Togni's reagent as both the radical initiator and a CF_3_ source, providing an efficient protocol for building up 1-indanone framework with wide functional group compatibility. The reaction mechanism was proposed through a radical triggered addition/5-exo-dig cyclization/oxidation/nucleophilic cascade. nt protocol for building up 1-indanone framework with wide functional group compatibility. The reaction mechanism was proposed through a radical triggered addition/5-exo-dig cyclization/oxidation/nucleophilic cascade.

## Introduction

**Figure 1:**
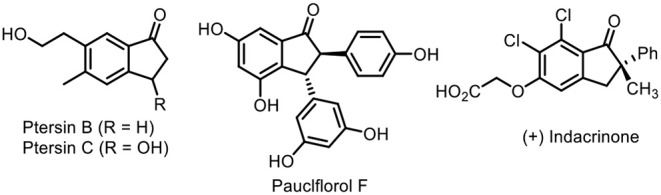
1-Indanone-containing natural products.

Trifluoromethylation of organic molecular skeletons has attracted considerable attention in pharmaceutical chemistry, agrochemicals, and functional materials, owing to the fact that incorporation of the trifluoromethyl group into organic molecules can modulate their abilities including lipophilicity, bioavailability, and metabolic stability (Umemoto, 1996; Müller et al., 2007; Hagmann, 2008; Studer, 2012; Yang et al., 2015). Therefore, many efforts have been done in the past few decades, which mainly depended on transition-metal-catalyzed trifluoromethylation reactions. Such reactions enable direct construction of the C–CF_3_ bond in an atom-economic manner and provide efficient and practical methods for the collection of trifluoromethyl-containing compounds, such as catalytic trifluoromethylation of alkane (Pan et al., 2011; Fu et al., 2012; Kuninobu et al., 2015; Wang et al., 2015; Xiao et al., 2019), alkenes (Chu and Qing, 2012; Shimizu et al., 2012; Zhu and Buchwald, 2013; Lin et al., 2016; He et al., 2018), and alkynes (Ge et al., 2014; Iqbal et al., 2014; Tomita et al., 2015; Wu et al., 2017; Huang et al., 2018). Among them, a vast majority of reports focused on the difunctionalization of alkenes or enynes (He et al., 2014a,b), such as hydrotrifluoromethylation (Wilger et al., 2013; Wu et al., 2013), carbotrifluoromethylation (Chen et al., 2013; Egami et al., 2013; Liu et al., 2013), and oxytrifluoromethylation (Egami et al., 2012; Janson et al., 2012; Li and Studer, 2012; Zhu and Buchwald, 2012) for their high utilization by incorporating trifluoromethyl groups into target molecules across the unsaturated π system. On the other hand, 1-indanones are privileged structural motifs commonly present in many bioactive molecules and natural products such as Pterosin B and C (Nagle et al., 2000; Wessig and Teubner, 2006), pauciflorol F (Dai et al., 1998; Nitta et al., 2002; Ito et al., 2004), and (+)-indacrinone (DeSolms et al., 1978) (Figure 1). Consequently, many chemists made their contributions to establish numerous elegant protocols for their synthesis including Friedel–Crafts acylation (Koelsch, 1932; Frank et al., 1944), Grignard reactions (Bergmann, 1956; Manning et al., 1981), and transition metal-catalyzed annulation of arylalkynes (Shintani et al., 2007; Chernyak et al., 2011; He et al., 2018; Song et al., 2019), radical addition–cyclization of 1,6-enynes (Shen et al., 2018a,b, 2019), and other methods (Zhu et al., 2017, 2018a,b; Shi et al., 2019a). To the best of our knowledge, introduction of a trifluoromethyl group into the 1-indanone framework via a radical-triggered annulation–difunctionalization strategy remains elusive.

**Scheme 1:**
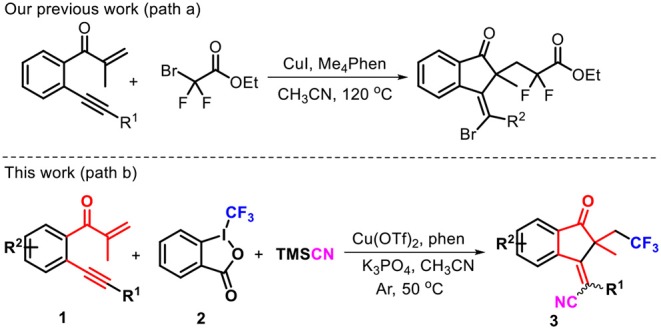
Profiles for annulation–difunctionalization of 1,6-enynes.

Multicomponent reactions (MCRs) represent an attractive and powerful tool for building complex molecular architectures under usually mild conditions (Hao et al., 2016; Wang et al., 2016a,b; Ji et al., 2019; Liu et al., 2019; Qin et al., 2019; Shi et al., 2019b). Radical-triggered annulation–difunctionalization cascades, standing at the intersection of both radical and multicomponent transformations, constitute a unique reaction category, which enables direct assembly of difunctionalized cyclic systems containing both isocyclic and heterocyclic skeletons which are not available from other methods. As a result, lots of unsaturated compounds endowed with alkene and/or alkyne units are devised and prepared as radical acceptors to capture the various radical species (Chen et al., 2008; Liu et al., 2014; Kong et al., 2015; Wang F. et al., 2016; Zhang et al., 2019). Generally, the success of the radical annulation–difunctionalization relies on the radical continuous transfer across the unsaturated systems through a synergistic orientation process. Over the years, our group has been heavily involved in the development of new annulation–difunctionalization cascades for multiple ring formations. For example, we reported a copper-catalyzed annulation–halofluoroalkylation of 1,6-enynes, leading to the atom-economic and highly stereoselective protocol toward functionalized 1-indenones (Scheme 1, path a) (Shen et al., 2019). To continue our interest in this project, we approach a radical addition–cyclization strategy to install both trifluoromethyl and cyano moieties into the 1-indenone framework, due to the behaviors of trifluoromethyl and cyano groups in the wide application potentiality in assigning and discovering new biological lead compounds. An extensive literature survey revealed that the radical-triggered annulation–cyanotrifluoromethylation of 1,6-enynes toward 1-indanones remains unreported to date. For this reason, the copper-catalyzed annulation–cyanotrifluoromethylation of 1,6-enynes **1** with Togni's reagent **2a** and trimethylsilyl cyanide (TMSCN) was carried out by 1,10-phenanthroline (phen) as the ligand, enabling a radical-induced three-component cascade to access trifluoromethylated 1-indanones **3** with generally good yields (Scheme 1, path b). Remarkably, some cases showed complete stereoselectivity, and only *E*-selectivity was observed. Herein, we report this copper-catalyzed radical transformation.

## Results and Discussion

At the outset of our studies, we chose the preformed 1,6-enyne **1a**, Togni's reagent **2a**, and TMSCN as the model substrate (Table 1). To our delight, the reaction of **1a** with **2a** and TMSCN in a 1:2:2 mol ratio catalyzed by 10 mol% Cu(OAc)_2_ proceeded smoothly in acetonitrile at 50°C by using 1,10-phenanthroline (phen, L_1_) as a ligand, and the target product **3a** as a sole (*E*)-stereoisomer was obtained in 36% yield. The following screening of solvents showed that the use of DMSO and DMF led to a slightly decreased yield of **3a** compared with acetonitrile (entries 2 and 3 vs. entry 1), whereas both 1,4-dioxane and THF completely suppressed the formation of **3a** (entries 4 and 5). Thus, acetonitrile was the best solvent for the reaction. An employment of NaOAc as the base facilitated the reaction process, delivering 40% yield of the desired product **3a** (entry 6). After that, we conducted the screening of a variety of copper salts, such as Cu(CH_3_CN)_4_PF_6_, CuCN, CuI, and Cu(OTf)_2_, that are often utilized in catalytic transformations, for this addition–cyclization cascade by using acetonitrile as the reaction medium. All these catalysts could promote the conversion of **1a** into **3a** (entries 7–10), and the latter one showed the best catalytic performance in the current reaction, generating product **3a** in 55% yield (entry 10). As the next optimization step, several ligands, such as 1,10-phenanthroline-5,6-dione (L_2_), 2,2′-bipyridine (L_3_), and 2,2′:6′,2″-terpyridine (L_4_), were investigated and anticipated to enhance the yield of product **3a**. Disappointingly, ligands L_2_-L_4_ showed slightly weaker performance on the conversion of **1a** into **3a** as compared with L_1_ (entries 11–13). Using Togni's reagent **2b** to replace **2a** resulted in a lower conversion (42%, entry 14 vs. entry 10). Different bases such as potassium phosphate tribasic (K_3_PO_4_), trimethylamine (Et_3_N), and cesium carbonate (Cs_2_CO_3_) were then screened. The results indicated that K_3_PO_4_ could improve the reaction, providing product **3a** in 64% yield. After careful optimizations, we found that fine-tuning the substrate ratio **1a**/**2a**/TMSCN to 1:3:2 delivered product **3a** in a higher 87% yield (entry 18).

**Table 1:** Optimization of reaction conditions^[a]^.

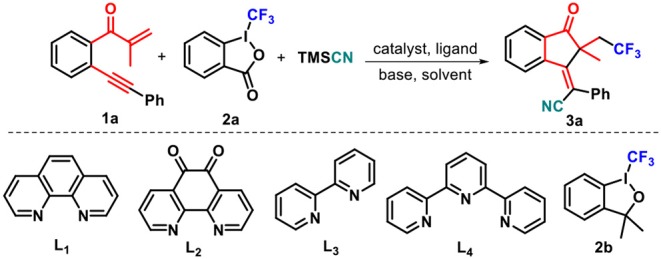					
**Entry**	**Cat. (mol%)**	**Ligand (mol%)**	**Solvent**	**Base (equiv)**	**Yield (%)**^***b***^
1	Cu(OAc)_2_ (10)	**L**_**1**_ (20)	CH_3_CN	–	36
2	Cu(OAc)_2_ (10)	**L**_**1**_ (20)	DMSO	–	34
3	Cu(OAc)_2_ (10)	**L**_**1**_ (20)	DMF	–	31
4	Cu(OAc)_2_ (10)	**L**_**1**_ (20)	1,4-Dioxane	–	NR
5	Cu(OAc)_2_ (10)	**L**_**1**_ (20)	THF	–	ND
6	Cu(OAc)_2_ (10)	**L**_**1**_ (20)	CH_3_CN	NaOAc (2)	40
7	Cu(CH_3_CN)_4_PF_6_ (10)	**L**_**1**_ (20)	CH_3_CN	NaOAc (2)	41
8	CuCN (10)	**L**_**1**_ (20)	CH_3_CN	NaOAc (2)	48
9	CuI (10)	**L**_**1**_ (20)	CH_3_CN	NaOAc (2)	46
10	Cu(OTf)_2_ (10)	**L**_**1**_ (20)	CH_3_CN	NaOAc (2)	55
11	Cu(OTf)_2_ (10)	**L**_**2**_ (20)	CH_3_CN	NaOAc (2)	53
12	Cu(OTf)_2_ (10)	**L**_**3**_ (20)	CH_3_CN	NaOAc (2)	47
13	Cu(OTf)_2_ (10)	**L**_**4**_ (20)	CH_3_CN	NaOAc (2)	50
14^c^	Cu(OTf)_2_ (10)	**L**_**1**_ (20)	CH_3_CN	NaOAc (2)	42
15	Cu(OTf)_2_ (10)	**L**_**1**_ (20)	CH_3_CN	K_3_PO_4_ (2)	64
16	Cu(OTf)_2_ (10)	**L**_**1**_ (20)	CH_3_CN	Cs_2_CO_3_ (2)	52
17	Cu(OTf)_2_ (10)	**L**_**1**_ (20)	CH_3_CN	Et_3_N (2)	39
18^d^	Cu(OTf)_2_ (10)	**L**_**1**_ (20)	CH_3_CN	K_3_PO_4_ (2)	87

[a] *Reaction conditions: **1** (0.2 mmol), **2** (0.4 mmol), Cu(OTf)_2_ (10 mol%), **L1** (20 mol%), K_3_PO_4_ (0.4 mmol), acetonitrile (2.0 ml), TMSCN (0.4 mmol), Ar conditions at 50°C for 3 h*.

[b] *Isolated yield based on substrates **1***.

[c] *Umemoto's reagent **2b** was used*.

[d] *Mole ratio of **1a**, **2a**, and TMSCN in 1:3:2*.

**Scheme 2:**
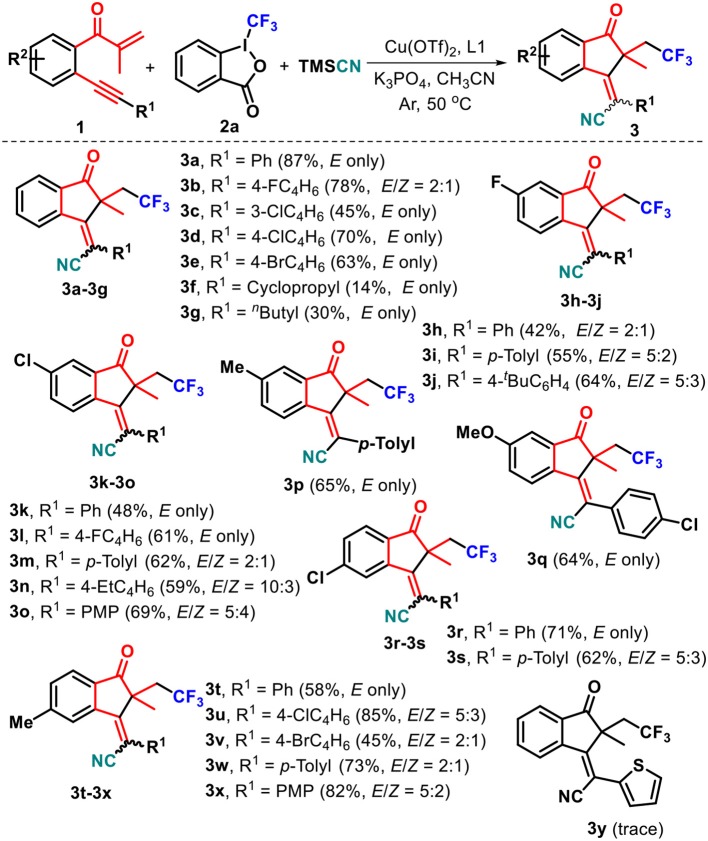
Substrate scope of 1,6-enynes.

With the optimized conditions in hand (Table 1, entry 18), the substrate scope of this radical-triggered annulation–cyanotrifluoromethylation of 1,6-enynes was investigated. The results were presented in Scheme 2. Upon repeating the reaction with **2a** and TMSCN, substrate **1** with diverse substituents such as fluoro (**1b**), chloro (**1c** and **1d**), and bromo (**1e**) groups on the arylalkynyl moiety all work well, giving the corresponding functionalized (*E*)-1-indanones **3b**–**3e** in 45–78% yields. Notably, substrates **1c**–**1e** could completely orient the *E*-selectivity to the target products **3c**–**3e**. Alternatively, both cyclopropyl **1f** and *n*-butyl **1g** counterparts were proven to be favorable, enabling radical-induced cyclization reactions to offer the corresponding (*E*)-1-indanones **3f** and **3g** with complete stereoselectivities, albeit with low yields. Due to the pharmacological significance of fluorine-containing molecules compared to their non-fluorinated analogs, we decided to prepare 1,6-enynes **1h**–**1j** containing the fluoro group residing in the 5-position of the internal arene ring and employed them to react with **2a** and TMSCN. The reaction worked well, accessing the corresponding polyfluoro products **3h**–**3j** in 42–64% yields and 5:3 to 5:2 *E*/*Z* ratios. Other substituents including chloro (**1k**–**1o**, **1r**, and **1s**), methyl (**1p** and **1t**–**1x**), and methoxy (**1q**) located at the C4- or C5-position on the internal arene ring did not hamper this copper-catalyzed reaction, and a range of new substituted 1-indanones **3k**–**3x** can be isolated in synthetically useful yields, in which a complete diastereoselectivity was also observed in the cases of **3k**, **3l**, **3p**, **3q**, **3r**, and **3t**. However, unsatisfactory *E*/*Z* ratios were detected for the other products. Either electronically neutral (H), poor (fluoro, chloro, and bromo), or rich [methyl, ethyl, *t*-butyl, and methoxy (PMP = *p*-methoxyphenyl)] groups at the *para*-position of the arylalkynyl moiety (R^1^) are well-tolerated with the catalytic conditions. Unfortunately, 1,6-enyne **1y** carrying a 2-thienyl group was an ineffective reaction partner in this transformation. The structures of these resulting 1-indanones were fully characterized by NMR spectroscopy and HRMS data (Data Sheet 1).

**Scheme 3:**
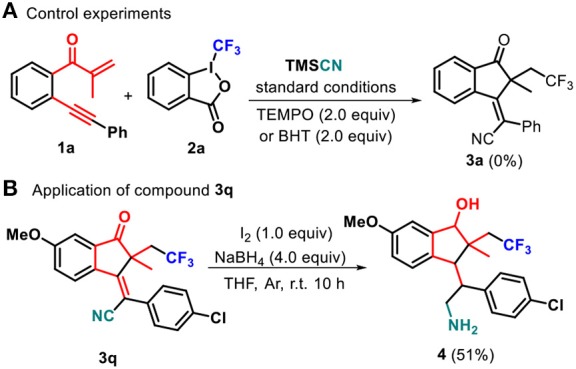
Control experiments and application of compound 3q.

To gain mechanistic insight into this transformation, radical trapping experiments were performed. When 2,2,6,6-tetramethylpiperidine-1-oxyl (TEMPO) as a radical scavenger was subjected to the reaction conditions, the generation of **3a** was completely suppressed (Scheme 3a). Similarly, BHT could inhibit the formation of **3a**. These results showed that the reaction may include a radical process. Moreover, the developed transformation could be valorized through post-functionalization of indanone **3q** (Scheme 3b). The combination of NaBH_4_ and I_2_ was found to be effective to reduce **3q** to give 2,3-dihydro-1*H*-inden-1-ol **4** (51% yield) (He et al., 2015; Chen et al., 2018).

## Mechanism

**Scheme 4:**
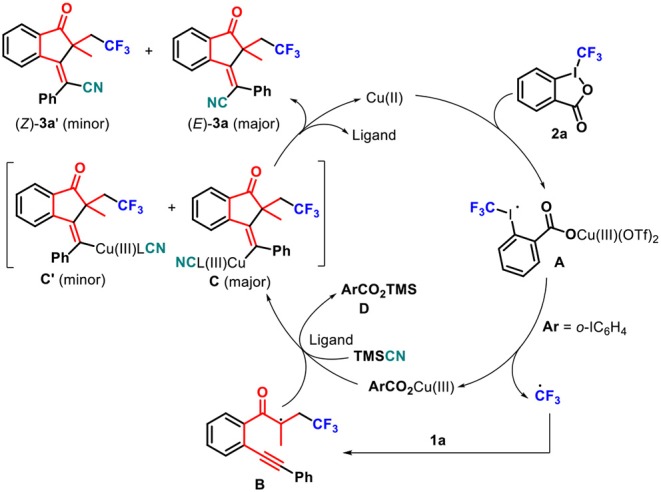
Proposed reaction mechanism.

According to these results and related literature (Kamigata et al., 1990; Liu et al., 2012; Pair et al., 2013; Yasu et al., 2013; He et al., 2014b; Shen et al., 2019), a plausible mechanism was proposed (Scheme 4). The copper(II) catalyst activates Togni's reagent **2a** to give the activated complex **A**, which releases a Cu(III) species and the trifluoromethyl radical. The latter rapidly adds to 1,6-enyne **1a** to give the radical intermediate **B**. In the presence of ligands and TMSCN, Cu(III) species activates an alkyne unit of intermediate **B** to drive 5-*exo-dig* cyclization, giving favorable *anti*-Cu(III) species **C** (Shen et al., 2019), some of which is converted into *syn*-Cu(III) species **C**′, together with trimethylsilyl 2-iodobenzoate **D**. Finally, anti-Cu(III) species **C** undergoes reductive elimination to give the desired (*E*)-product **3a** as a major isomer and regenerate a Cu(II) complex to complete a catalytic cycle through the release of ligands (He et al., 2014b), whereas *syn*-Cu(III) species **C**′ undergoes the same reductive elimination to access minor (*Z*)-product **3a**.

## Conclusion

In summary, we have established a copper-catalyzed annulation–cyanotrifluoromethylation of 1,6-enynes with easily available Togni's reagent and TMSCN, by which a wide range of 1-indanones with a quaternary carbon center were stereoselectively synthesized in generally good yields. Notably, a complete stereoselectivity could be detected in most cases. This approach is efficiently induced by Togni's reagent as a radical donor and ultimately terminated by TMSCN as the nucleophilic reagent. The transformation offered a new entry to prepare the CF_3_-containing 1-indanone skeleton via a complex radical addition–cyclization cascade. Further investigations into the mechanism and its application will be conducted in due course.

## Materials and Methods

### General

^1^H NMR (^13^C NMR, ^19^F NMR) spectra were measured on a Bruker DPX 400-MHz spectrometer in CDCl_3_ (DMSO-*d*_6_) with chemical shift (δ) given in ppm relative to TMS as internal standard [(s = singlet, d = doublet, t = triplet, brs = broad singlet, m = multiplet), coupling constant (Hz)]. HRMS (ESI) was done by using a micrOTOF-Q II HRMS/MS instrument (Bruker).

### General Procedure for the Synthesis of 3

#### Example for the Synthesis of 3a

Under Ar conditions, a mixture of Togni's reagent **2** (0.6 mmol), Cu(OTf)_2_ (0.02 mmol), K_3_PO_4_ (0.4 mmol), and ligand **L1** (0.04 mmol) was added in a Schlenk tube. Acetonitrile was added into the tube. Then, 2-methyl-1-[2-(phenylethynyl)phenyl]prop-2-en-1-one **1a** (0.2 mmol) and TMSCN (0.4 mmol) were put in the system, stirred for 3 h at 50°C until thin-layer chromatography (TLC) revealed that conversion of the starting material **1a** was complete. Next, the reaction mixture was concentrated in vacuum, and the resulting residue was purified by silica gel column chromatography (petroleum ether/ethyl acetate = 25:1, v/v) to afford the desired product **3a**.

### General Procedure for the Synthesis of 4

Under Ar conditions, **3q** (0.05 mmol), NaBH_4_ (3.0 equiv), and I_2_ (1.0 equiv) were added in a Schlenk tube. THF was added, and the reaction mixture was stirred at room temperature for 10 h. The solution was treated with water and extracted with dichloromethane. The combined organic layers were washed with brine, dried over Na_2_SO_4_, concentrated in vacuum, and purified by preparative TLC (petroleum ether/ethyl acetate = 2/1) to afford product **4** (He et al., 2015; Chen et al., 2018).

#### (*E*)-2-[2-Methyl-3-oxo-2-(2,2,2-trifluoroethyl)-2,3-dihydro-1*H*-inden-1-ylidene]-2-phenylacetonitrile (3a)

Light yellow solid, 59 mg, 87% yield; mp 105.2–106.1°C. ^1^H NMR (400 MHz, CDCl_3_; δ, ppm): 8.96 (d, *J* = 8.0 Hz, 1H), 7.94 (d, *J* = 7.6 Hz, 1H), 7.90–7.85 (m, 1H), 7.73–7.67 (m, 1H), 7.54–7.49 (m, 3H), 7.46–7.41 (m, 2H), 2.66–2.53 (m, 1H), 2.25–2.12 (m, 1H), 1.19 (s, 3H). ^13^C NMR (100 MHz, CDCl_3_; δ, ppm): 202.3, 153.2, 144.5, 136.3, 135.5, 133.0, 132.4, 129.9, 129.5, 129.2, 125.0 (q, *J* = 85.1 Hz, CF_3_), 118.9, 109.1, 49.8, 40.4 (q, *J* = 27.6 Hz, CH_2_CF_3_), 25.1. ^19^F NMR (376 MHz, CDCl_3_; δ, ppm): −61.29 (s, 3F). IR (KBr, ν, cm^−1^): 2,200, 1,721, 1,577, 1,447, 1,361, 1,256, 1,138, 967, 775. HRMS (ESI, *m*/*z*): calcd for C_20_H_14_F_3_NONa [M + Na]^+^ 364.0919, found 364.0928.

#### (*E*)-2-(4-Fluorophenyl)-2-[2-methyl-3-oxo-2-(2,2,2-trifluoroethyl)-2,3-dihydro-1*H*-inden-1-ylidene]acetonitrile (3b, Major)

Light yellow solid, 56 mg, 78% yield; mp 148.9–150.9°C. ^1^H NMR (400 MHz, CDCl_3_; δ, ppm): 8.94 (d, *J* = 8.0 Hz, 1H), 7.94 (d, *J* = 7.6 Hz, 1H), 7.86 (d, *J* = 8.0 Hz, 1H), 7.73–7.67 (m, 1H), 7.46–7.42 (m, 2H), 7.27–7.22 (m, 2H), 2.67–2.58 (m, 1H), 2.18–2.09 (m, 1H), 1.20 (s, 3H). ^13^C NMR (100 MHz, CDCl_3_; δ, ppm): 202.0, 163.3 (d, ^1^*J* = 247.0 Hz, CF), 153.9, 144.3, 136.3, 135.3, 132.6, 131.6 (d, ^3^*J* = 8.3 Hz, CF), 128.9 (d, ^4^*J* = 3.7 Hz, CF), 126.6, 125.0 (q, *J* = 80.7 Hz, CF_3_), 117.4, 117.2, 116.4 (d, ^2^*J* = 21.8 Hz, CF), 108.0, 49.8, 40.3 (q, *J* = 27.8 Hz, CH_2_CF_3_), 25.2. ^19^F NMR (376 MHz, CDCl_3_; δ, ppm): −61.31 (s, 3F), −109.82 (s, 1F). IR (KBr, ν, cm^−1^): 2,202, 1,724, 1,599, 1,509, 1,361, 1,257, 1,142, 1,070, 776. HRMS (ESI, *m*/*z*): calcd for C_20_H_13_F_4_NONa [M + Na]^+^ 382.0825, found 382.0784.

#### (*E*)-2-(3-Chlorophenyl)-2-[2-Methyl-3-oxo-2-(2,2,2-Trifluoroethyl)-2,3-Dihydro-1*H*-Inden-1-Ylidene]Acetonitril (3c)

Light yellow solid, 34 mg, 45% yield; mp 174.6–177.1°C. ^1^H NMR (400 MHz, CDCl_3_; δ, ppm): 8.94 (d, *J* = 8.0 Hz, 1H), 7.96 (d, *J* = 7.6 Hz, 1H), 7.91–7.86 (m, 1H), 7.75–7.69 (m, 1H), 7.52–7.42 (m, 3H), 7.34 (d, *J* = 7.2 Hz, 1H), 2.69–2.61 (m, 1H), 2.21–2.12 (m, 1H), 1.21 (s, 3H). ^13^C NMR (100 MHz, CDCl_3_; δ, ppm): 201.9, 154.0, 144.2, 136.4, 135.6, 135.2, 134.6, 132.7, 130.5. 130.2, 129.8, 127.7, 125.1 (q, *J* = 78.5 Hz, CF_3_), 118.5, 107.6, 49.8, 40.2 (q, *J* = 55.5 Hz, CH_2_CF_3_), 25.2. ^19^F NMR (376 MHz, CDCl_3_; δ, ppm): −61.31 (s, 3F). IR (KBr, ν, cm^−1^): 2,204, 1,728, 1,595, 1,336, 1,260, 1,140, 1,069, 776. HRMS (ESI, *m*/*z*): calcd for C_20_H_13_ClF_3_NONa [M + Na]^+^ 398.0529, found 398.0520.

####  (*E*)-2-(4-Chlorophenyl)-2-[2-Methyl-3-oxo-2-(2,2,2-Trifluoroethyl)-2,3-Dihydro-1*H*-Inden-1-ylidene]Acetonitrile (3d)

Light yellow solid, 53 mg, 70% yield; mp 154.5–155.3°C. ^1^H NMR (400 MHz, CDCl_3_; δ, ppm): 8.94 (d, *J* = 8.4 Hz, 1H), 7.95 (d, *J* = 7.6 Hz, 1H), 7.91–7.85 (m, 1H), 7.75–7.69 (m, 1H), 7.50 (d, *J* = 8.4 Hz, 2H), 7.39 (d, *J* = 8.4 Hz, 2H), 2.69–2.59 (m, 1H), 2.20–2.11 (m, 1H), 1.20 (s, 3H). ^13^C NMR (100 MHz, CDCl_3_; δ, ppm): 201.9, 153.9, 144.2, 136.4, 136.3, 135.6, 132.7, 131.4, 131.0, 129.6, 125.1 (q, *J* = 78.5 Hz, CF_3_), 118.6, 107.8, 49.8, 40.7 (q, *J* = 27.7 Hz, CH_2_CF_3_), 25.2. ^19^F NMR (376 MHz, CDCl_3_; δ, ppm): −61.28 (s, 3F). IR (KBr, ν, cm^−1^): 2,204, 1,729, 1,593, 1,491, 1,360, 1,256, 1,143, 1,072, 835, 776. HRMS (ESI, *m*/*z*): calcd for C_20_H_13_ClF_3_NONa [M + Na]^+^ 398.0529, found 398.0569.

####  (*E*)-2-(4-Bromophenyl)-2-[2-Methyl-3-oxo-2-(2,2,2-Trifluoroethyl)-2,3-dihydro-1*H*-inden-1-ylidene]Acetonitrile (3e)

Light yellow solid, 53 mg, 63% yield; mp 103.9–104.7°C. ^1^H NMR (400 MHz, CDCl_3_; δ, ppm): 8.93 (d, *J* = 8.0 Hz, 1H), 7.95 (d, *J* = 7.6 Hz, 1H), 7.91–7.85 (m, 1H), 7.74–7.68 (m, 1H), 7.66 (d, *J* = 8.4 Hz, 2H), 7.32 (d, *J* = 8.4 Hz, 2H), 2.71–2.59 (m, 1H), 2.21–2.11 (m, 1H), 1.20 (s, 3H). ^13^C NMR (100 MHz, CDCl_3_; δ, ppm): 201.9, 153.9, 144.2, 136.4, 135.6, 132.7, 132.5, 131.9, 131.2, 125.1 (q, *J* = 77.7 Hz, CF_3_), 124.5, 118.5, 107.8, 49.8, 40.4 (q, *J* = 27.8 Hz, CH_2_CF_3_), 25.2. ^19^F NMR (376 MHz, CDCl_3_; δ, ppm): −61.27 (s, 3F). IR (KBr, ν, cm^−1^): 2,205, 1,728, 1,585, 1,486, 1,360, 1,255, 1,142, 1,069, 1,011, 968, 832, 723. HRMS (ESI, *m*/*z*): calcd for C_20_H_13_BrF_3_NONa [M + Na]^+^ 442.0024, found 442.0020.

#### (*E*)-2-Cyclopropyl-2-[2-Methyl-3-oxo-2-(2,2,2-trifluoroethyl)-2,3-dihydro-1*H*-inden-1-ylidene]Acetonitrile (3f)

Light yellow oil, 9 mg, 14% yield. ^1^H NMR (400 MHz, CDCl_3_; δ, ppm): 7.82 (d, *J* = 7.6 Hz, 1H), 7.72–7.61 (m, 2H), 7.46–7.39 (m, 1H), 5.99–5.89 (m, 1H), 2.86–2.77 (m, 1H), 2.65–2.58 (m, 1H), 2.58–2.46 (m, 4H), 1.33 (s, 3H). ^13^C NMR (100 MHz, CDCl_3_; δ, ppm): 203.5, 146.2, 135.9, 129.2, 124.4 (q, *J* = 28.8 Hz, CF_3_), 118.9, 110.0, 97.0, 48.1, 40.0 (q, *J* = 28.1 Hz, CH_2_CF_3_), 25.3, 25.2, 17.1. ^19^F NMR (376 MHz, CDCl_3_; δ, ppm): −61.78 (s, 3F). IR (KBr, ν, cm^−1^): 2,248, 1,964, 1,719, 1,602, 1,471, 1,362, 1,261, 1,142, 1,069, 799. HRMS (ESI, *m*/*z*): calcd for C_17_H_14_F_3_NONa [M + Na]^+^ 328.0920, found 328.0856.

#### (*E*)-2-[2-Methyl-3-oxo-2-(2,2,2-Trifluoroethyl)-2,3-dihydro-1*H*-inden-1-ylidene]hexanenitrile (3g)

Light yellow oil, 19 mg, 30% yield. ^1^H NMR (400 MHz, CDCl_3_; δ, ppm): 8.88 (d, *J* = 8.0 Hz, 1H), 7.90 (d, *J* = 7.6 Hz, 1H), 7.83–7.77 (m, 1H), 7.64–7.59 (m, 1H), 3.08–2.99 (m, 1H), 2.82–2.72 (m, 1H), 2.57–2.48 (m, 2H), 1.83–1.69 (m, 2H), 1.49–1.45 (m, 2H), 1.41 (s, 3H), 1.01 (t, *J* = 7.2 Hz, 3H). ^13^C NMR (100 MHz, CDCl_3_; δ, ppm): 202.7, 149.9, 144.8, 136.2, 134.8, 131.6, 124.8 (q, *J* = 49.1 Hz, CF_3_), 118.8, 110.3, 49.2, 40.3 (q, *J* = 25.2 Hz, CH_2_CF_3_), 31.8, 30.0, 23.7, 22.6, 13.9. ^19^F NMR (376 MHz, CDCl_3_; δ, ppm): −62.09 (s, 3F). IR (KBr, ν, cm^−1^): 2,210, 1,731, 1,596, 1,469, 1,365, 1,257, 1,142, 1,070, 777. HRMS (ESI, *m*/*z*): calcd for C_18_H_18_F_3_NONa [M + Na]^+^ 344.1213, found 344.1197.

####  (*E*)-2-[5-Fluoro-2-methyl-3-oxo-2-(2,2,2-trifluoroethyl)-2,3-dihydro-1*H*-inden-1-ylidene]-2-phenylacetonitrile (3h, Major)

Light yellow solid, 30 mg, 42% yield; mp 107.0–109.0°C. ^1^H NMR (400 MHz, CDCl_3_; δ, ppm): 9.00–8.94 (m, 1H), 7.59–7.55 (m, 2H), 7.51–7.48 (m, 3H), 7.44–7.42 (m, 2H), 2.62–2.53 (m, 1H), 2.24–2.15 (m, 1H), 1.19 (s, 3H). ^13^C NMR (100 MHz, CDCl_3_; δ, ppm): 201.3, 165.0 (d, ^1^*J* = 256.3 Hz, CF), 152.1, 140.6 (d, ^5^*J* = 2.5 Hz, CF), 132.7, 130.1, 129.2, 127.8 (d, ^4^*J* = 8.6 Hz, CF), 126.3, 123.5, 123.3 (d, ^2^*J* = 26.8 Hz, CF), 118.9, 110.6 (d, ^3^*J* = 22.2 Hz, CF), 50.4, 40.5 (q, *J* = 27.8 Hz, CH_2_CF_3_), 25.1. ^19^F NMR (376 MHz, CDCl_3_; δ, ppm): −61.30 (s, 3F), −104.65 (s, 1F). IR (KBr, ν, cm^−1^): 2,205, 1,732, 1,600, 1,488, 1,362, 1,257, 1,186, 1,141, 1,067, 949, 833. HRMS (ESI, *m*/*z*): calcd for C_20_H_13_F_4_NONa [M + Na]^+^ 382.0825, found 382.0832.

#### (*E*)-2[(5-Fluoro-2-methyl-3-oxo-2-(2,2,2-trifluoroethyl)-2,3-dihydro-1*H*-inden-1-ylidene]-2-(*p*-tolyl)acetonitrile (3i, Major)

Light yellow solid, 41 mg, 55% yield; mp 1,041–105.8°C. ^1^H NMR (400 MHz, CDCl_3_; δ, ppm): 8.98–8.93 (m, 1H), 7.58–7.52 (m, 2H), 7.35–7.30 (m, 4H), 2.62–2.52 (m, 1H), 2.43 (s, 3H), 2.27–2.18 (m, 1H), 1.20 (s, 3H). ^13^C NMR (100 MHz, CDCl_3_; δ, ppm): 201.5, 164.9 (d, ^1^*J* = 256.2 Hz, CF), 152.0, 140.2, 130.8, 129.9, 129.3, 127.7 (d, ^4^*J* = 8.6 Hz, CF), 126.3, 123.5, 123.2 (d, ^2^*J* = 23.6 Hz, CF), 119.0, 110.5 (d, ^3^*J* = 22.2 Hz, CF), 50.4, 40.4 (q, *J* = 27.7 Hz, CH_2_CF_3_), 25.1, 21.4. ^19^F NMR (376 MHz, CDCl_3_; δ, ppm): −61.29 (s, 3F), −104.91 (s, 1F). IR (KBr, ν, cm^−1^): 2,201, 1,729, 1,596, 1,447, 1,361, 1,256, 1,178, 1,138, 1,069, 967, 775, 712. HRMS (ESI, *m*/*z*): calcd for C_21_H_15_F_4_NONa [M + Na]^+^ 396.0982, found 396.0956.

#### (*E*)-2-[4-(tert-Butyl)phenyl]-2-[5-fluoro-2-methyl-3-oxo-2-(2,2,2-trifluoroethyl)-2,3-dihydro-1*H*-inden-1-ylidene]acetonitrile (3j, Major)

Light yellow oil, 53 mg, 64% yield. ^1^H NMR (400 MHz, CDCl_3_; δ, ppm): 8.99–8.92 (m, 1H), 7.53–7.48 (m, 3H), 7.36–7.32 (m, 3H), 2.63–2.53 (m, 1H), 2.31–2.18 (m, 1H), 1.37 (s, 9H), 1.20 (s, 3H). ^13^C NMR (100 MHz, CDCl_3_; δ, ppm): 201.5, 164.9 (d, ^1^*J* = 256.0 Hz, CF), 153.4, 140.8 (d, ^5^*J* = 2.6 Hz, CF), 129.7, 129.1, 128.3, 127.7 (d, ^4^*J* = 8.5 Hz, CF), 126.1, 123.5, 123.2 (d, ^2^*J* = 23.6 Hz, CF), 119.0, 110.5 (d, ^3^*J* = 22.2 Hz, CF), 50.4, 40.6 (q, *J* = 27.7 Hz, CH_2_CF_3_), 34.9, 31.3, 25.0. ^19^F NMR (376 MHz, CDCl_3_; δ, ppm): −61.26 (s, 3F), −104.94 (s, 1F). IR (KBr, ν, cm^−1^): 2,206, 1,734, 1,599, 1,487, 1,362, 1,258, 1,187, 1,141, 1,071, 949, 808. HRMS (ESI, *m*/*z*): calcd for C_24_H_21_F_4_NONa [M + Na]^+^ 438.1451, found 438.1458.

####  (*E*)-2-[5-Chloro-2-methyl-3-oxo-2-(2,2,2-trifluoroethyl)-2,3-dihydro-1*H*-inden-1-ylidene]-2-phenylacetonitrile (3k)

Light yellow solid, 36 mg, 48% yield; mp 144.7–146.9°C. ^1^H NMR (400 MHz, CDCl_3_; δ, ppm): 8.89 (d, *J* = 8.8 Hz, 1H), 7.89 (d, *J* = 2.0 Hz, 1H), 7.84–7.78 (m, 1H), 7.54–7.48 (m, 3H), 7.46–7.38 (m, 2H), 2.63–2.52 (m, 1H), 2.24–2.13 (m, 1H), 1.19 (s, 3H). ^13^C NMR (100 MHz, CDCl_3_; δ, ppm): 201.1, 152.1, 142.7, 139.1, 137.0, 136.3, 132.7, 130.1, 129.4, 129.2, 126.7, 124.4, 118.7, 109.6, 50.2, 40.5 (q, *J* = 27.8 Hz, CH_2_CF_3_), 25.1. ^19^F NMR (376 MHz, CDCl_3_; δ, ppm): −61.25 (s, 3F). IR (KBr, ν, cm^−1^): 2,205, 1,726, 1,588, 1,457, 1,419, 1,364, 1,264, 1,179, 1,142, 1,068, 836, 703. HRMS (ESI, *m*/*z*): calcd for C_20_H_13_ClF_3_NONa [M + Na]^+^ 398.0530, found 398.0491.

####  (*E*)-2-[5-Chloro-2-methyl-3-oxo-2-(2,2,2-trifluoroethyl)-2,3-dihydro-1*H*-inden-1-ylidene]-2-(4-fluorophenyl)acetonitrile (3l)

Light yellow solid, 48 mg, 61% yield; mp 195.2–197.2°C. ^1^H NMR (400 MHz, CDCl_3_; δ, ppm): 8.88 (d, *J* = 8.8 Hz, 1H), 7.89 (d, *J* = 2.0 Hz, 1H), 7.84–7.78 (m, 1H), 7.45–7.38 (m, 2H), 7.25–7.18 (m, 2H), 2.68–2.55 (m, 1H), 2.20–2.08 (m, 1H), 1.20 (s, 3H). ^13^C NMR (100 MHz, CDCl_3_; δ, ppm): 200.9, 163.4 (d, ^1^*J* = 250.2 Hz, CF), 152.8, 142.4, 139.4, 137.0, 136.4, 131.5 (d, ^2^*J* = 8.4 Hz, CF), 128.6 (d, ^3^*J* = 3.6 Hz, CF), 126.7, 124.5, 118.6, 116.7, 116.4, 108.5, 50.1, 40.4 (q, *J* = 27.8 Hz, CH_2_CF_3_), 25.1. ^19^F NMR (376 MHz, CDCl_3_; δ, ppm): −61.27(s, 3F), −109.49 (s, 1F). IR (KBr, ν, cm^−1^): 2,209, 1,727, 1,588, 1,507, 1,426, 1,361, 1,263, 1,139, 1,064, 835. HRMS (ESI, *m*/*z*): calcd for C_20_H_12_ClF_4_NONa [M + Na]^+^ 416.0436, found 416.0421.

#### (*E*)-2-[5-Chloro-2-methyl-3-oxo-2-(2,2,2-trifluoroethyl)-2,3-dihydro-1*H*-inden-1-ylidene]-2-(*p*-tolyl)acetonitrile (3m, Major)

Light yellow solid, 48 mg, 62% yield; mp 121.7–123.6°C. ^1^H NMR (400 MHz, CDCl_3_; δ, ppm): 8.88 (d, *J* = 8.8 Hz, 1H), 7.88 (d, *J* = 2.0 Hz, 1H), 7.81–7.78 (m, 1H), 7.30 (s, 4H), 2.61–2.52 (m, 1H), 2.43 (s, 3H), 2.26–2.18 (m, 1H), 1.20 (s, 3H). ^13^C NMR (100 MHz, CDCl_3_; δ, ppm): 201.3, 152.0, 142.8, 140.2, 139.0, 136.3, 135.4, 130.8, 129.9, 129.3, 126.7, 124.3, 118.8, 109.7, 50.2, 40.4 (q, *J* = 27.6 Hz, CH_2_CF_3_), 25.2, 21.4. ^19^F NMR (376 MHz, CDCl_3_; δ, ppm): −61.25 (s, 3F). IR (KBr, ν, cm^−1^): 2,205, 1,732, 1,589, 1,508, 1,457, 1,361, 1,263, 1,178, 1,144, 1,070, 942, 833. HRMS (ESI, *m*/*z*): calcd for C_21_H_15_ClF_3_NONa [M + Na]^+^ 412.0686, found 412.0657.

####  (*E*)-2-[5-Chloro-2-methyl-3-oxo-2-(2,2,2-trifluoroethyl)-2,3-dihydro-1*H*-inden-1-ylidene]-2-(4-ethylphenyl)acetonitrile (3n, Major)

Light yellow solid, 48 mg, 59% yield; mp 117.8–120.4°C. ^1^H NMR (400 MHz, CDCl_3_; δ, ppm): 8.88 (d, *J* = 8.8 Hz, 1H), 7.88 (d, *J* = 2.0 Hz, 1H), 7.82–7.78 (m, 1H), 7.32 (s, 4H), 2.75–2.68 (m, 2H), 2.61–2.53 (m, 1H), 2.27–2.18 (m, 1H), 1.30 (t, *J* = 7.6 Hz, 3H), 1.20 (s, 3H). ^13^C NMR (100 MHz, CDCl_3_; δ, ppm): 201.3, 152.0, 146.5, 142.8, 139.0, 136.9, 136.3, 129.6, 129.3, 128.7, 126.7, 124.3, 118.9, 109.8, 50.2, 40.4 (q, *J* = 27.76 Hz, CH_2_CF_3_), 28.7, 25.1, 15.3. ^19^F NMR (376 MHz, CDCl_3_; δ, ppm): −61.24 (s, 3F). IR (KBr, ν, cm^−1^): 2,203, 1,732, 1,587, 1,507, 1,457, 1,362, 1,254, 1,179, 1,145, 1,070, 942, 833. HRMS (ESI, *m*/*z*): calcd for C_22_H_17_ClF_3_NONa [M + Na]^+^ 426.0843, found 426.0824.

#### (*E*)-2-[5-Chloro-2-methyl-3-oxo-2-(2,2,2-trifluoroethyl)-2,3-dihydro-1*H*-inden-1-ylidene]-2-(4-methoxyphenyl)acetonitrile (3o, Major)

Light yellow solid, 56 mg, 69% yield; mp 118.4–120.8°C. ^1^H NMR (400 MHz, CDCl_3_; δ, ppm): 8.87 (d, *J* = 8.8 Hz, 1H), 7.87 (d, *J* = 2.0 Hz, 1H), 7.83–7.79 (m, 1H), 7.36–7.33 (m, 2H), 7.02–6.98 (m, 2H), 3.87 (s, 3H), 2.62–2.54 (m, 1H), 2.27–2.18 (m, 1H), 1.20 (s, 3H). ^13^C NMR (100 MHz, CDCl_3_; δ, ppm): 201.3, 160.7, 152.1, 142.8, 138.9, 136.9, 136.3, 130.8, 126.7, 125.5, 124.3, 118.9, 114.6, 109.5, 55.4, 50.2, 40.3 (q, *J* = 27.8 Hz, CH_2_CF_3_), 25.2. ^19^F NMR (376 MHz, CDCl_3_; δ, ppm): −61.26 (s, 3F). IR (KBr, ν, cm^−1^): 2,203, 1,732, 1,602, 1,508, 1,457, 1,362, 1,255, 1,177, 1,144, 1,069, 833. HRMS (ESI, *m*/*z*): calcd for C_21_H_15_ClF_3_NO_2_Na [M + Na]^+^ 428.0636, found 428.0623.

####  (*E*)-2-[2,5-Dimethyl-3-oxo-2-(2,2,2-trifluoroethyl)-2,3-dihydro-1*H*-inden-1-ylidene]-2-(*p*-tolyl)acetonitrile (3p)

Light yellow solid, 48 mg, 65% yield; mp 174.5–176.6°C. ^1^H NMR (400 MHz, CDCl_3_; δ, ppm): 8.83 (d, *J* = 8.8 Hz, 1H), 7.41–7.37 (m, 1H), 7.31 (d, *J* = 2.4 Hz, 1H), 7.29 (s, 3H), 7.26 (s, 1H), 3.94 (s, 3H), 2.60–2.50 (m, 1H), 2.42 (s, 3H), 2.27–2.16 (m, 1H), 1.19 (s, 3H). ^13^C NMR (100 MHz, CDCl_3_; δ, ppm): 202.4, 163.0, 152.7, 139.8, 138.0, 137.7, 130.2, 129.8, 129.6, 126.8, 125.2, 119.5, 106.5, 105.6, 56.0, 50.3, 40.3 (q, *J* = 27.6 Hz, CH_2_CF_3_), 25.2, 21.4. ^19^F NMR (376 MHz, CDCl_3_; δ, ppm): −61.38 (s, 3F). IR (KBr, ν, cm^−1^): 2,201, 1,725, 1,594, 1,486, 1,362, 1,296, 1,231, 1,146, 1,069, 832. HRMS (ESI, *m*/*z*): calcd for C_22_H_18_F_3_NONa [M + Na]^+^ 392.1233, found 392.1257.

####  (*E*)-2-(4-Chlorophenyl)-2-[5-methoxy-2-methyl-3-oxo-2-(2,2,2-trifluoroethyl)-2,3-dihydro-1*H*-inden-1-ylidene]acetonitrile (3q)

Light yellow solid, 52 mg, 64% yield; mp 130.5–131.2°C. ^1^H NMR (400 MHz, CDCl_3_; δ, ppm): 8.83 (d, *J* = 8.8 Hz, 1H), 7.48 (d, *J* = 8.4 Hz, 2H), 7.40 (d, *J* = 6.4 Hz, 1H), 7.39–7.35 (m, 2H), 7.32 (d, *J* = 2.4 Hz, 1H), 3.94 (s, 3H), 2.68–2.56 (m, 1H), 2.20–2.08 (m, 1H), 1.19 (s, 3H). ^13^C NMR (100 MHz, CDCl_3_; δ, ppm): 201.9, 163.3, 153.5, 137.8, 137.5, 136.1, 131.6, 131.2, 129.5, 126.0 (q, *J* = 160.5 Hz, CF_3_), 119.0, 105.8, 105.0, 56.0, 50.3, 40.3 (q, *J* = 27.7 Hz, CH_2_CF_3_), 25.2. ^19^F NMR (376 MHz, CDCl_3_; δ, ppm): −61.38 (s, 3F). IR (KBr, ν, cm^−1^): 2,202, 1,727, 1,595, 1,488, 1,364, 1,295, 1,143, 1,019, 845. HRMS (ESI, *m*/*z*): calcd for C_21_H_15_ClF_3_NO_2_Na [M + Na]^+^ 428.0636, found 428.0616.

#### (*E*)-2-[6-Chloro-2-methyl-3-oxo-2-(2,2,2-trifluoroethyl)-2,3-dihydro-1*H*-inden-1-ylidene]-2-phenylacetonitrile (3r)

Light yellow solid, 53 mg, 71% yield; mp 161.6–163.8°C. ^1^H NMR (400 MHz, CDCl_3_; δ, ppm): 8.94 (d, *J* = 1.2 Hz, 1H), 7.88 (d, *J* = 8.0 Hz, 1H), 7.69–7.64 (m, 1H), 7.54–7.50 (m, 3H), 7.45–7.39 (m, 2H), 2.62–2.54 (m, 1H), 2.22–2.13 (m, 1H), 1.19 (s, 3H). ^13^C NMR (100 MHz, CDCl_3_; δ, ppm): 201.0, 151.9, 145.7, 143.3, 133.8, 133.0, 132.6, 130.1, 129.4, 129.3, 125.6, 125.5, 118.4, 110.5, 50.0, 40.4 (q, *J* = 27.7 Hz, CH_2_CF_3_), 25.2. ^19^F NMR (376 MHz, CDCl_3_; δ, ppm): −61.25 (s, 3F). IR (KBr, ν, cm^−1^): 2,202, 1,724, 1,589, 1,489, 1,361, 1,271, 1,139, 1,072, 835, 712. HRMS (ESI, *m*/*z*): calcd for C_20_H_13_ClF_3_NONa [M + Na]^+^ 398.0530, found 398.0556.

####  (*E*)-2-[6-Chloro-2-methyl-3-oxo-2-(2,2,2-trifluoroethyl)-2,3-dihydro-1*H*-inden-1-ylidene]-2-(*p*-tolyl)acetonitrile (3s, Major)

Light yellow solid, 48 mg, 62% yield; mp 121.5–123.1°C. ^1^H NMR (400 MHz, CDCl_3_; δ, ppm): 8.92 (s, 1H), 7.86 (d, *J* = 8.0 Hz, 1H), 7.65 (d, *J* = 8.4 Hz, 1H), 7.36 (d, *J* = 7.6 Hz, 1H), 7.30 (s, 3H), 2.61–2.52 (m, 1H), 2.43 (s, 3H), 2.26–2.17 (m, 1H), 1.19 (s, 3H). ^13^C NMR (100 MHz, CDCl_3_; δ, ppm): 201.1, 151.7, 145.8, 143.2, 140.3, 133.8, 132.9, 130.8, 129.9, 126.8, 125.6, 118.5, 110.7, 50.1, 40.4 (q, *J* = 27.7 Hz, CH_2_CF_3_), 25.2, 21.4. ^19^F NMR (376 MHz, CDCl_3_; δ, ppm): −61.24 (s, 3F). IR (KBr, ν, cm^−1^): 2,205, 1,731, 1,588, 1,509, 1,456, 1,362, 1,255, 1,145, 1,072, 825. HRMS (ESI, *m*/*z*): calcd for C_21_H_15_ClF_3_NONa [M + Na]^+^ 412.0686, found 412.0686.

#### (*E*)-2-[2,6-Dimethyl-3-oxo-2-(2,2,2-trifluoroethyl)-2,3-dihydro-1*H*-inden-1-ylidene]-2-phenylacetonitrile (3t)

Light yellow solid, 41 mg, 58% yield; mp 147.9–150.4°C. ^1^H NMR (400 MHz, CDCl_3_; δ, ppm): 8.74 (s, 1H), 7.83 (d, *J* = 8.0 Hz, 1H), 7.53–7.48 (m, 4H), 7.45–7.41 (m, 2H), 2.62–2.54 (m, 4H), 2.21–2.11 (m, 1H), 1.17 (s, 3H). ^13^C NMR (100 MHz, CDCl_3_; δ, ppm): 201.7, 153.3, 147.9, 144.8, 133.7, 133.5, 133.1, 129.8, 129.6, 129.2, 125.5 (q, *J* = 112.6 Hz, CF_3_), 119.0, 108.7, 50.0, 40.3 (q, *J* = 27.6 Hz, CH_2_CF_3_), 25.2, 22.6. ^19^F NMR (376 MHz, CDCl_3_; δ, ppm): −61.33 (s, 3F). IR (KBr, ν, cm^−1^): 2,202, 1,716, 1,613, 1,489, 1,455, 1,360, 1,253, 1,136, 1,072, 831, 767. HRMS (ESI, *m*/*z*): calcd for C_21_H_16_F_3_NONa [M + Na]^+^ 378.1076, found 378.1054.

#### (*E*)-2-(4-Chlorophenyl)-2-[2,6-dimethyl-3-oxo-2-(2,2,2-trifluoroethyl)-2,3-dihydro-1*H*-inden-1-ylidene]acetonitrile (3u, Major)

Light yellow solid, 66 mg, 85% yield; mp 108.1–110.8°C. ^1^H NMR (400 MHz, CDCl_3_; δ, ppm): 8.72 (s, 1H), 7.84 (d, *J* = 8.0 Hz, 1H), 7.52–7.48 (m, 3H), 7.40 (d, *J* = 6.8 Hz, 2H), 2.66–2.58 (m, 4H), 2.17–2.08 (m, 1H), 1.18 (s, 3H). ^13^C NMR (100 MHz, CDCl_3_; δ, ppm): 201.3, 154.0, 148.0, 144.6, 136.2, 134.0, 133.8, 132.3, 131.0, 129.5, 126.9, 125.0 (q, *J* = 106.5 Hz, CF_3_), 124.6, 118.6, 107.4, 50.0, 40.4 (q, *J* = 27.6 Hz, CH_2_CF_3_), 25.2, 22.6. ^19^F NMR (376 MHz, CDCl_3_; δ, ppm): −61.33 (s, 3F). IR (KBr, ν, cm^−1^): 2,208, 1,727, 1,595, 1,489, 1,360, 1,253, 1,180, 1,142, 1,071, 832. HRMS (ESI, *m*/*z*): calcd for C_21_H_15_ClF_3_NONa [M + Na]^+^ 412.0686, found 412.0637.

####  (*E*)-2-(4-Bromophenyl)-2-[2,6-dimethyl-3-oxo-2-(2,2,2-trifluoroethyl)-2,3-dihydro-1*H*-inden-1-ylidene]acetonitrile (3v, Major)

Light yellow solid, 39 mg, 45% yield; mp 136.5–138.6°C. ^1^H NMR (400 MHz, CDCl_3_; δ, ppm): 8.71 (s, 1H), 7.83 (d, *J* = 8.0 Hz, 1H), 7.65 (d, *J* = 8.4 Hz, 2H), 7.52 (d, *J* = 8.0 Hz, 1H), 7.31 (d, *J* = 8.4 Hz, 2H), 2.66–2.61 (m, 1H), 2.58 (s, 3H), 2.17–2.09 (m, 1H), 1.18 (s, 3H). ^13^C NMR (100 MHz, CDCl_3_; δ, ppm): 201.3, 154.0, 145.0, 144.6, 134.0, 133.8, 133.2, 132.5, 131.2, 126.9, 125.5 (q, *J* = 105.5 Hz, CF_3_), 124.6, 118.5, 107.4, 50.0, 40.4 (q, *J* = 27.7 Hz, CH_2_CF_3_), 25.2, 22.6. ^19^F NMR (376 MHz, CDCl_3_; δ, ppm): −61.31 (s, 3F). IR (KBr, ν, cm^−1^): 2,206, 1,731, 1,593, 1,456, 1,362, 1,255, 1,141, 1,070, 1,011, 831. HRMS (ESI, *m*/*z*): calcd for C_21_H_15_BrF_3_NONa [M + Na]^+^ 456.0181, found 456.0137.

####  (*E*)-2-[2,6-Dimethyl-3-oxo-2-(2,2,2-trifluoroethyl)-2,3-dihydro-1*H*-inden-1-ylidene]-2-(*p*-tolyl)acetonitrile (3w, Major)

Light yellow solid, 54 mg, 73% yield; mp 127.8–129.9°C. ^1^H NMR (400 MHz, CDCl_3_; δ, ppm): 8.73 (s, 1H), 7.82 (d, *J* = 8.0 Hz, 1H), 7.49 (d, *J* = 8.0 Hz, 1H), 7.30 (s, 4H), 2.61–2.53 (m, 4H), 2.43 (s, 3H), 2.24–2.17 (m, 1H), 1.18 (s, 3H). ^13^C NMR (100 MHz, CDCl_3_; δ, ppm): 201.9, 153.2, 147.8, 144.9, 140.0, 133.6, 133.3, 130.5, 129.8, 129.4, 127.1, 125.4 (q, *J* = 114.4 Hz, CF_3_), 119.1, 108.9, 50.0, 40.3 (q, *J* = 27.5 Hz, CH_2_CF_3_), 25.2, 22.6, 21.4. ^19^F NMR (376 MHz, CDCl_3_; δ, ppm): −61.32 (s, 3F). IR (KBr, ν, cm^−1^): 2,204, 1,719, 1,609, 1,590, 1,510, 1,456, 1,361, 1,254, 1,144, 1,071, 830. HRMS (ESI, *m*/*z*): calcd for C_22_H_18_F_3_NONa [M + Na]^+^ 392.1233, found 392.1223.

####  (*E*)-2-[2,6-Dimethyl-3-oxo-2-(2,2,2-trifluoroethyl)-2,3-dihydro-1*H*-inden-1-ylidene]-2-(4-methoxyphenyl)acetonitrile (3x, Major)

Light yellow solid, 63 mg, 82% yield; mp 120.8–122.4°C. ^1^H NMR (400 MHz, CDCl_3_; δ, ppm): 8.72 (s, 1H), 7.82 (d, *J* = 8.0 Hz, 1H), 7.49 (d, *J* = 8.0 Hz, 1H), 7.36 (d, *J* = 8.4 Hz, 2H), 7.00 (d, *J* = 8.8 Hz, 2H), 3.87 (s, 3H), 2.62–2.55 (m, 4H), 2.24–2.19 (m, 1H), 1.19 (s, 3H). ^13^C NMR (100 MHz, CDCl_3_; δ, ppm): 201.9, 160.5, 153.3, 147.8, 144.9, 133.6, 133.3, 130.9, 127.0, 125.5 (q, *J* = 114.2 Hz, CF_3_), 125.1, 119.2, 114.5, 108.6, 55.4, 50.1, 40.2 (q, *J* = 27.7 Hz, CH_2_CF_3_), 25.2, 22.6. ^19^F NMR (376 MHz, CDCl_3_; δ, ppm): −61.32 (s, 3F). IR (KBr, ν, cm^−1^): 2,205, 1,724, 1,605, 1,507, 1,457, 1,362, 1,257, 1,141, 1,070, 1,026, 832. HRMS (ESI, *m*/*z*): calcd for C_22_H_18_F_3_NO_2_Na [M + Na]^+^ 408.1182, found 408.1182.

#### 3-[2-Amino-1-(4-chlorophenyl)ethyl]-6-methoxy-2-methyl-2-(2,2,2-trifluoroethyl)-2,3-dihydro-1*H*-inden-1-ol (4)

White oil, 11 mg, 51% yield. ^1^H NMR (400 MHz, CDCl_3_; δ, ppm): 8.39 (d, *J* = 8.4 Hz, 1H), 7.47–7.41 (m, 2H), 7.35–7.30 (m, 2H), 6.99 (s, 2H), 4.78 (s, 1H), 3.89 (s, 3H), 3.70–3.66 (m, 1H), 3.25–3.21 (m, 1H), 2.37–2.23 (m, 2H), 1.95–1.91 (m, 1H), 1.78 (s, 2H), 1.70–1.66 (m, 1H), 1.07 (s, 3H). ^13^C NMR (100 MHz, *d*_6_-DMSO; δ, ppm): 162.8, 160.4, 151.7, 134.3, 133.5, 130.7 (q, *J* = 154.3 Hz, CF_3_), 128.2, 126.1, 120.2, 115.7, 109.0, 102.2, 79.4, 67.5, 56.0, 53.7, 25.6, 24.2. ^19^F NMR (376 MHz, *d*_6_-DMSO; δ, ppm): −58.24 (s, 3F). HRMS (ESI, *m*/*z*): calcd for C_21_H_23_ClF_3_NNaO_2_ [M + Na]^+^ 436.8508, found 436.8517.

## Data Availability Statement

The datasets generated for this study are available on request to the corresponding author.

## Author Contributions

T-SZ, BJ, and P-JC designed the project. T-SZ performed the experiments. T-SZ, W-JH, S-JT, and P-JC analyzed the data. T-SZ, BJ, and GL wrote the manuscript.

## Conflict of Interest

The authors declare that the research was conducted in the absence of any commercial or financial relationships that could be construed as a potential conflict of interest.
