# Supplementary material for: Copper-Catalyzed Annulation–Cyanotrifluoromethylation of 1,6-Enynes Toward 1-Indanones via a Radical Process
Source: Front Chem. 2020 Apr 17;8:234. doi: 10.3389/fchem.2020.00234 (PMC7180230; doi:10.3389/fchem.2020.00234)
Supplement: Supplementary file 1 [file Data_Sheet_1.PDF]

Supporting Information

Copper-Catalyzed Annulation-Cyanotrifluoromethylation of 1,6-Enynes toward 1-indanones via a Radical Process

Tian-Shu Zhang[1], Wen-Juan Hao[2], Pei-Jun Cai[1]*, Guigen Li[3,4], Shu-Jiang Tu[2], Bo Jiang[2]*

[1]School of Chemical Engineering & Technology, China University of Mining and Technology, Xuzhou, 221116, P. R. China.

[2]School of Chemistry & Materials Science, Jiangsu Key Laboratory of Green Synthetic Chemistry for Functional Materials, Jiangsu Normal University, Xuzhou 221116, P. R. China

[3]Institute of Chemistry & BioMedical Sciences, Collaborative Innovation Center of Chemistry for Life Sciences, Nanjing University, Nanjing 210093, P. R. China

[4]Department of Chemistry and Biochemistry, Texas Tech University, Lubbock, Texas 79409-1061, United States.

Email: pjcai@cumt.edu.cn(P.-J. Cai); jiangchem@jsnu.edu.cn (B. Jiang)

Context

General Procedure for the Synthesis of Compounds **1a**-**1y**.. S2

Copies of ^{1}H NMR,^{13}C NMR and F^{19} NMR Spectra for Products **3a-3x**........................ S3-S74

Copies of ^{1}H NMR,^{13}C NMR and F^{19} NMR Spectra for Products **4**................................ S75-S77

General Procedure for the Synthesis of Compounds 1a-1y

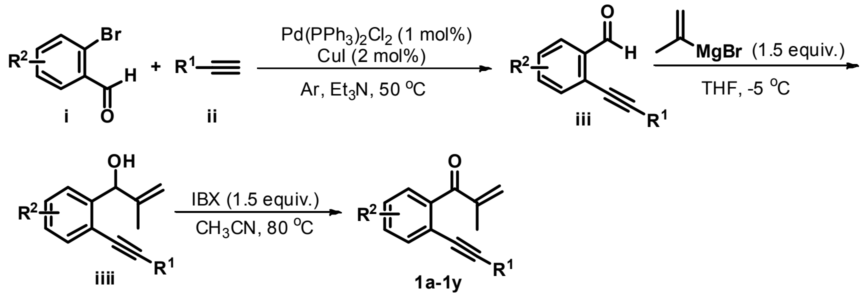

A mixture of $Pd(PPh_3)Cl_2$ (1 mol %), CuI (2 mol %), 2-bromobenzaldehyde **i** (1.0 equiv, 10 mmol), Et_3N (10 mL) and ethynylaryl **ii** (1.0 equiv, 10 mmol). Then, the reaction was stirred for 4 h at 50 °C. The reaction process was determined by TLC until the starting material consumed completely. The resulting mixture was extracted with 20 mL H_2O and 20 mL ethyl acetate for three times. The organic phase was concentrated to a bottle and was dried over $MgSO_4$. Then, the mixture was filtrated and the colature was evaporated on a rotary evaporator. The crude product was purified by chromatography on silica gel with petroleum ether/ethyl acetate (15:1) as the eluent to afford compound **iii.**

Then, **iii** was dissolved in 5mL THF and added to a flame-dried flask equipped with a magnetic stir bar. After that, 10 mL (1.0 mol/L) isopropenylmagnesium bromide solution was added to the flask by dropwise at -0 °C and stirred for 30 mins. The resulting mixture was extracted with 20 mL H_2O and 20 mL ethyl acetate for three times. The organic phase was concentrated to a bottle and was dried over $MgSO_4$. Then, the mixture was filtrated and the colature was evaporated on a rotary evaporator. The crude product was purified by chromatography on silica gel with petroleum ether/ethyl acetate (15:1) as the eluent to afford compound **iiii**.

iiii was dissolved in 10 mL CH_3CN, and IBX (1.5 equiv, 12.0 mmol) was loaded in the mixture, which was stirred at 80 °C for 2 h. The resulting mixture was extracted with 20 mL H_2O and 20 mL ethyl acetate for three times. The organic phase was concentrated to a bottle and was dried over $MgSO_4$. The organic phase was concentrated and evaporated on a rotary evaporator. The crude product was purified by chromatography on silica gel with petroleum ether/ethyl acetate (20:1) as the eluent to afford compound **1a-1y**.

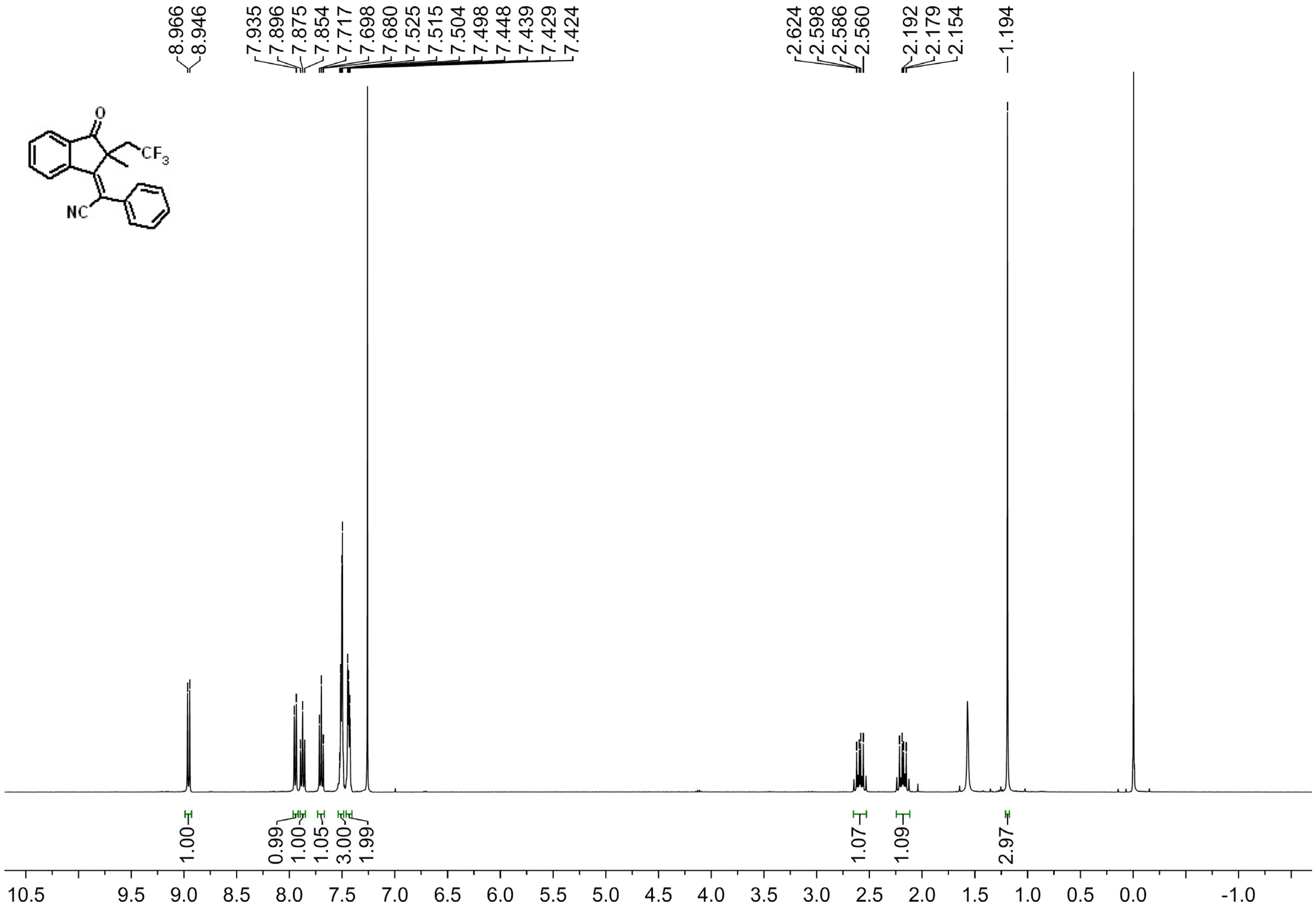

^{1}H NMR Spectrum of Compound **3a**

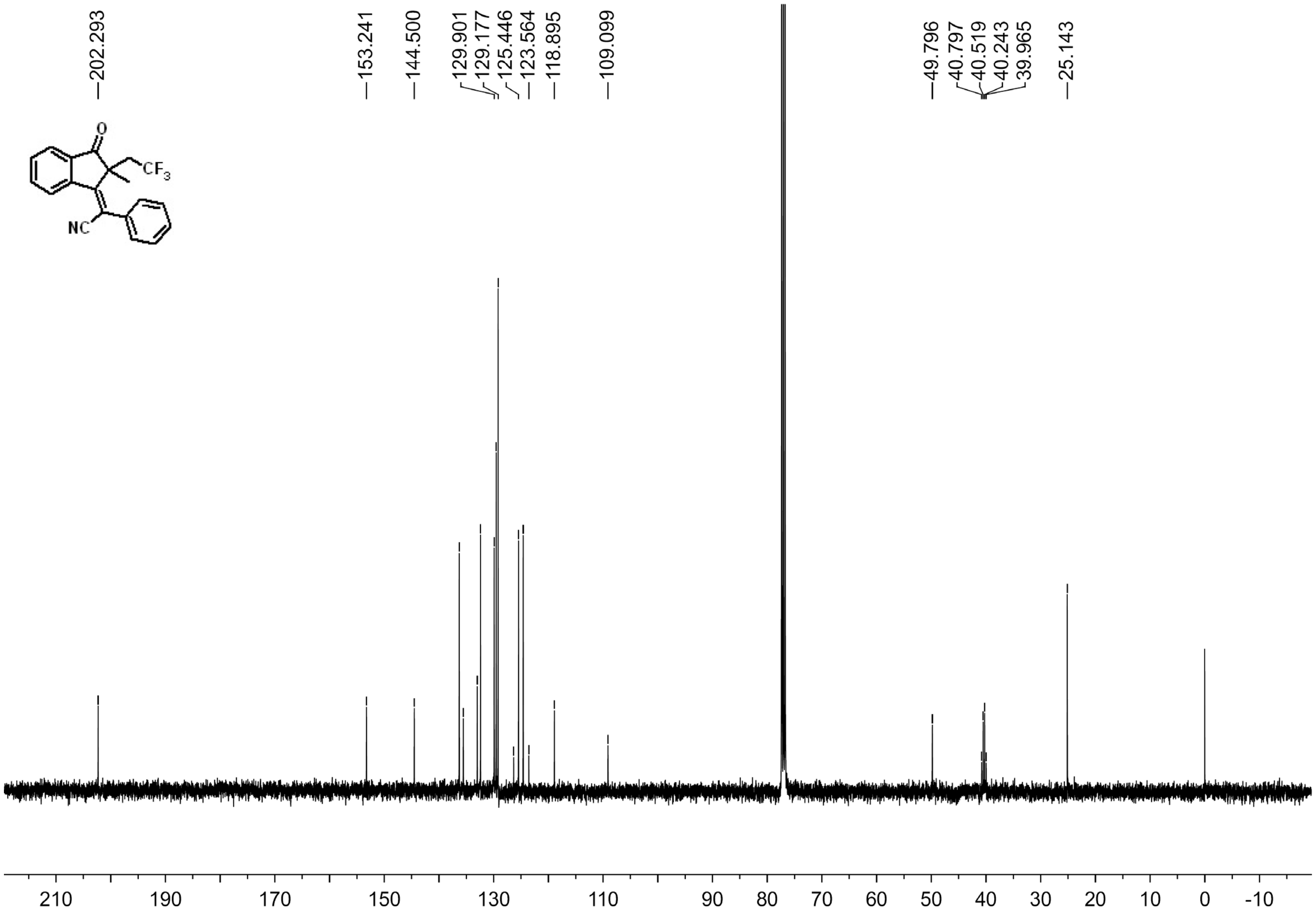

^{13}C NMR Spectrum of Compound **3a**

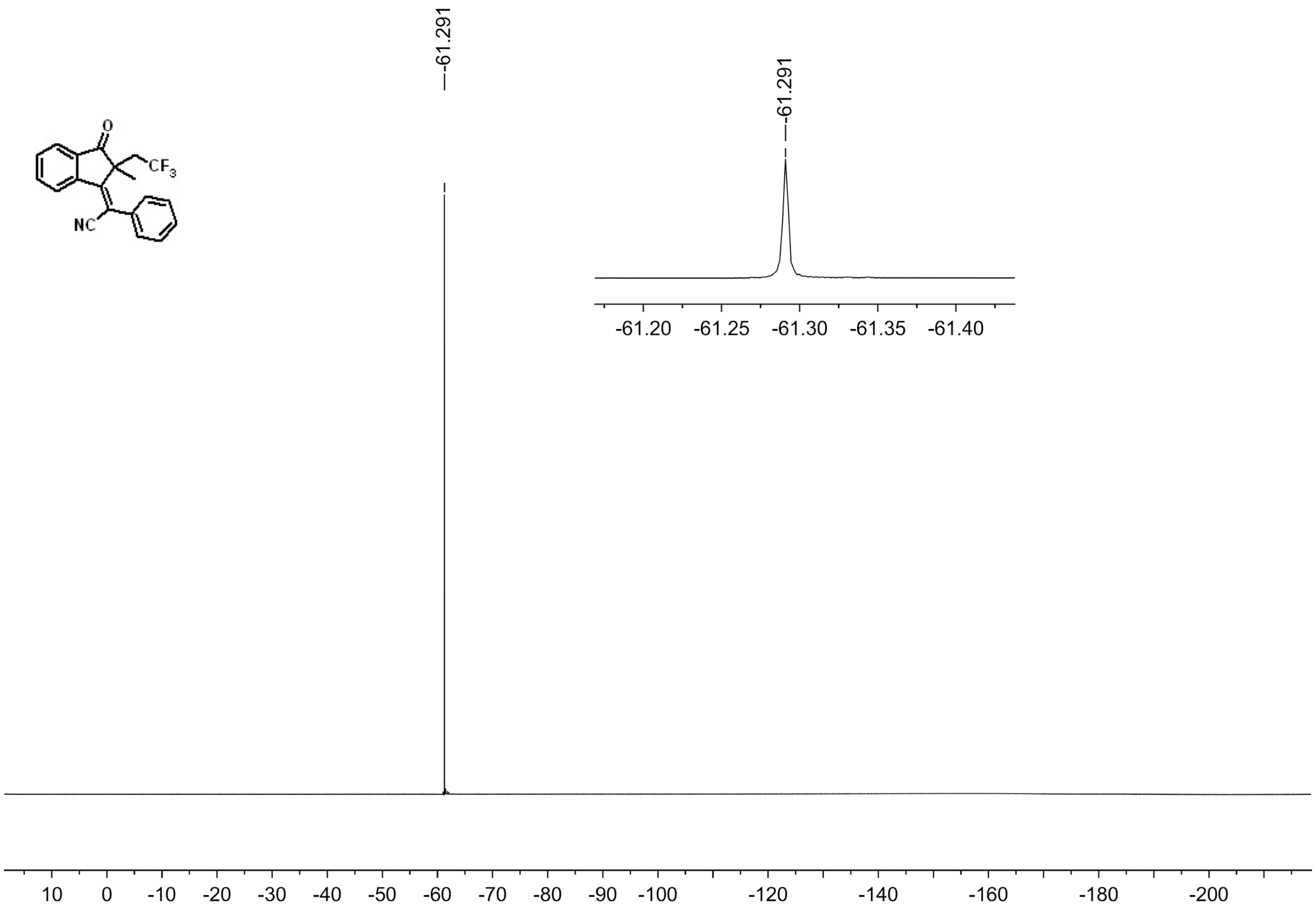

^{19}F NMR Spectrum of Compound **3a**

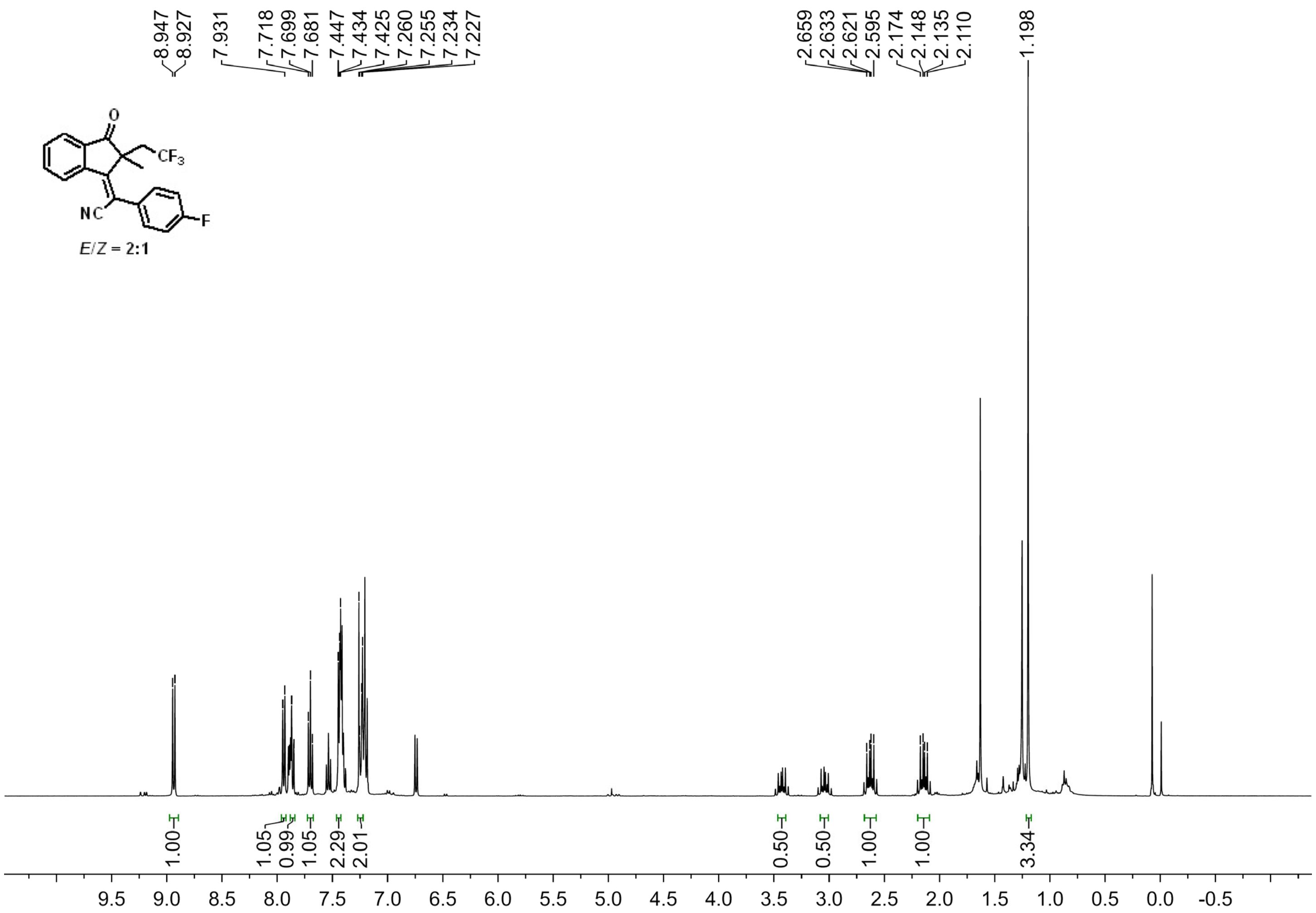

^{1}H NMR Spectrum of Compound **3b**

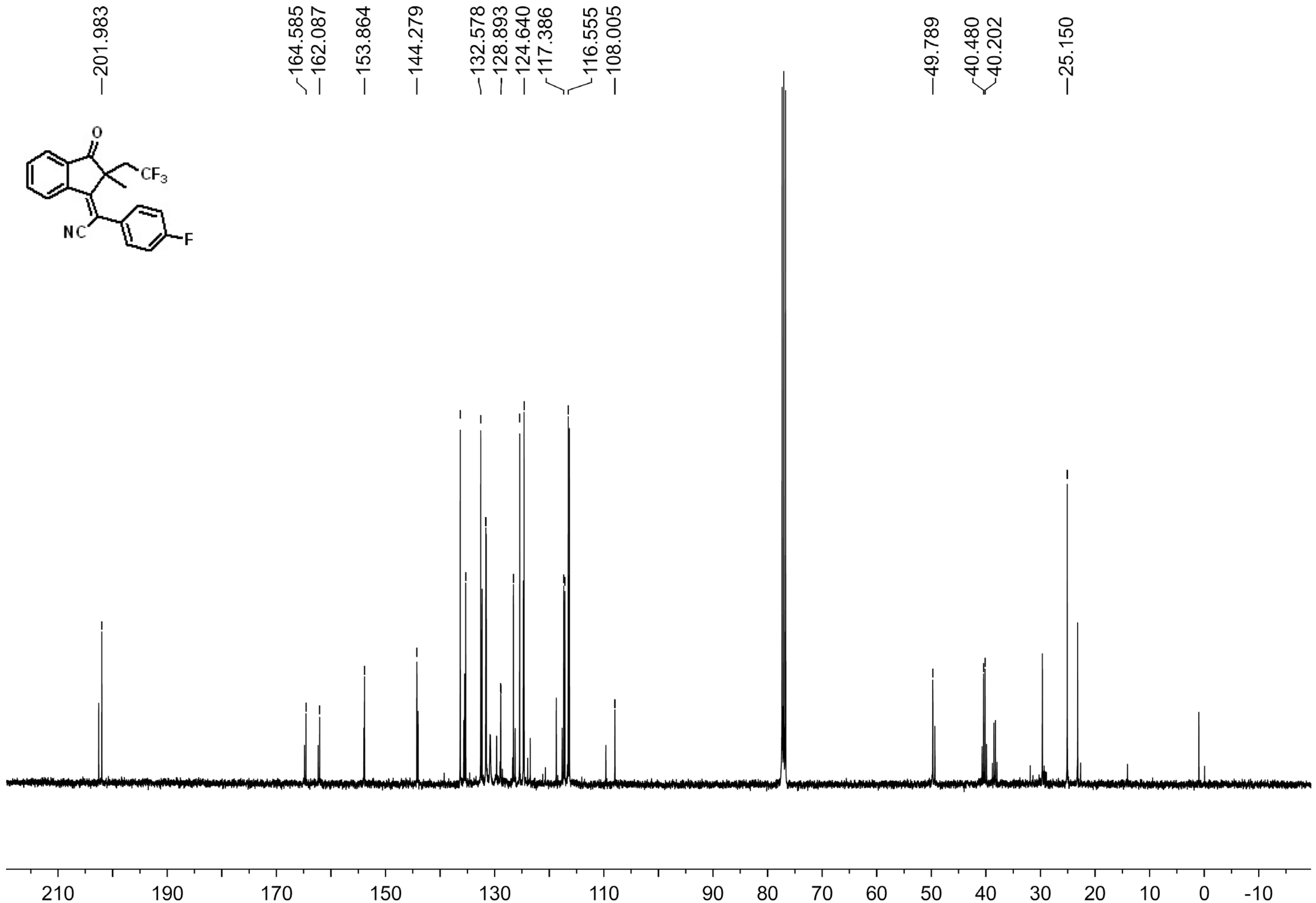

^{13}C NMR Spectrum of Compound **3b**

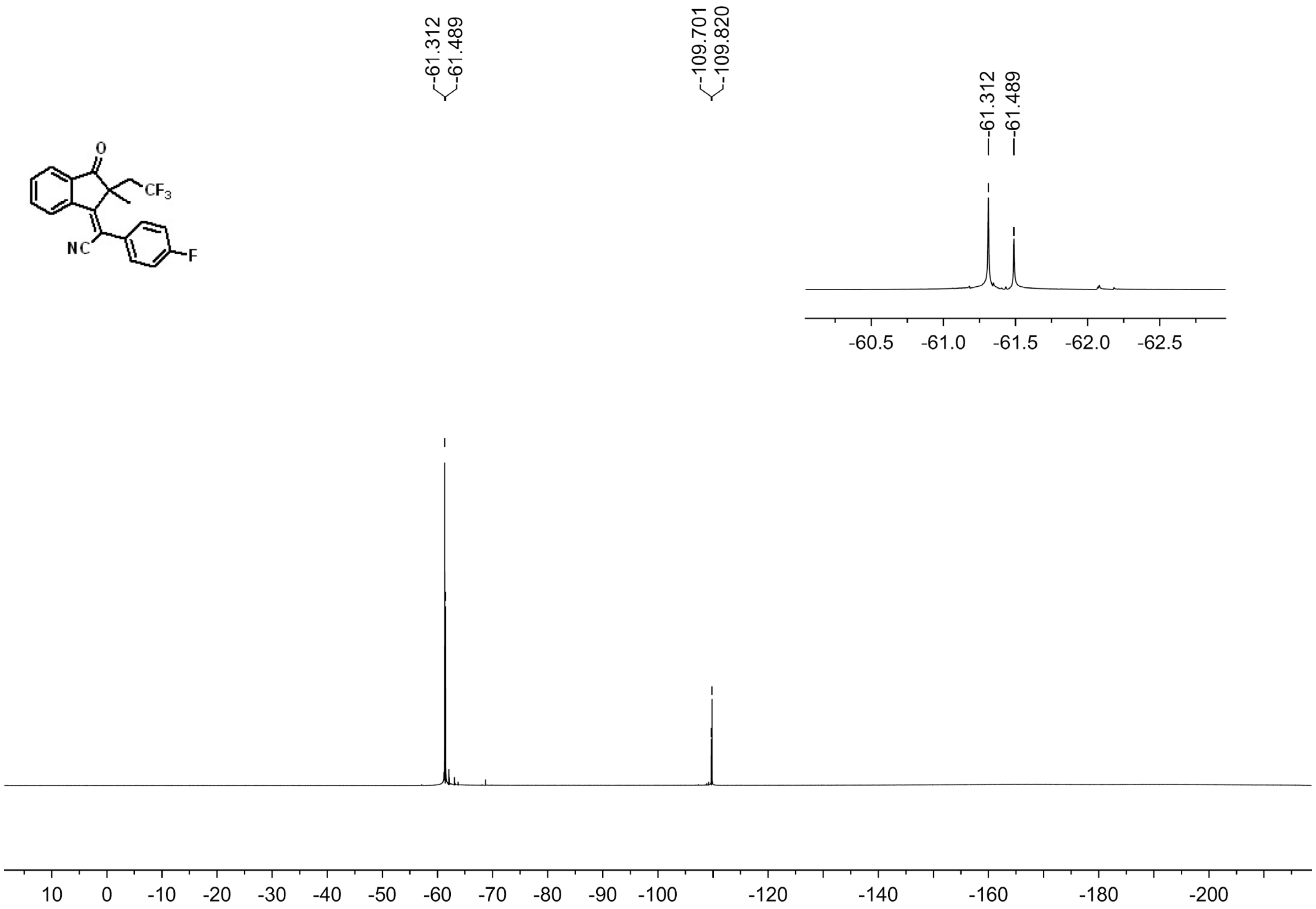

^{19}F NMR Spectrum of Compound **3b**

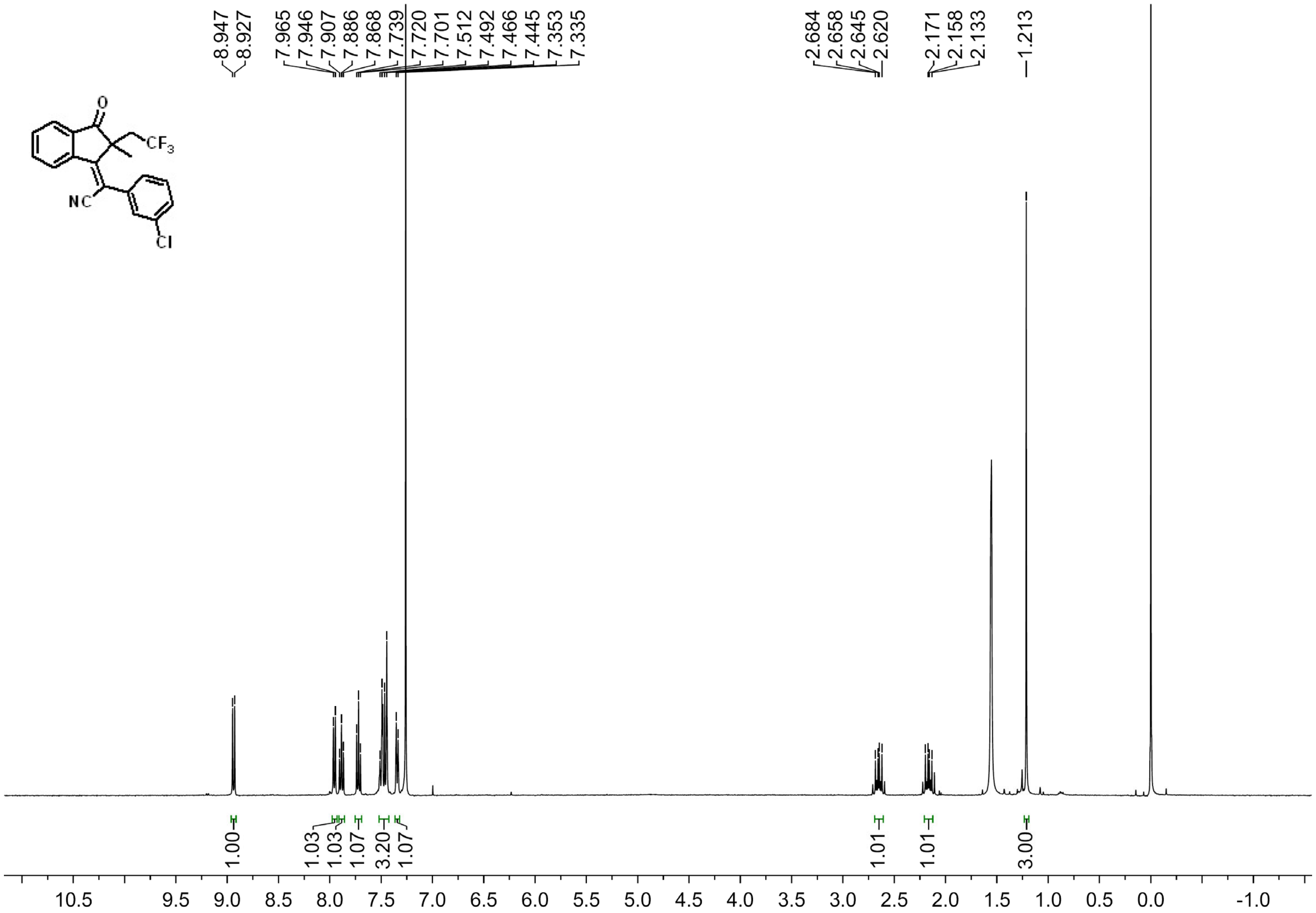

^{1}H NMR Spectrum of Compound **3c**

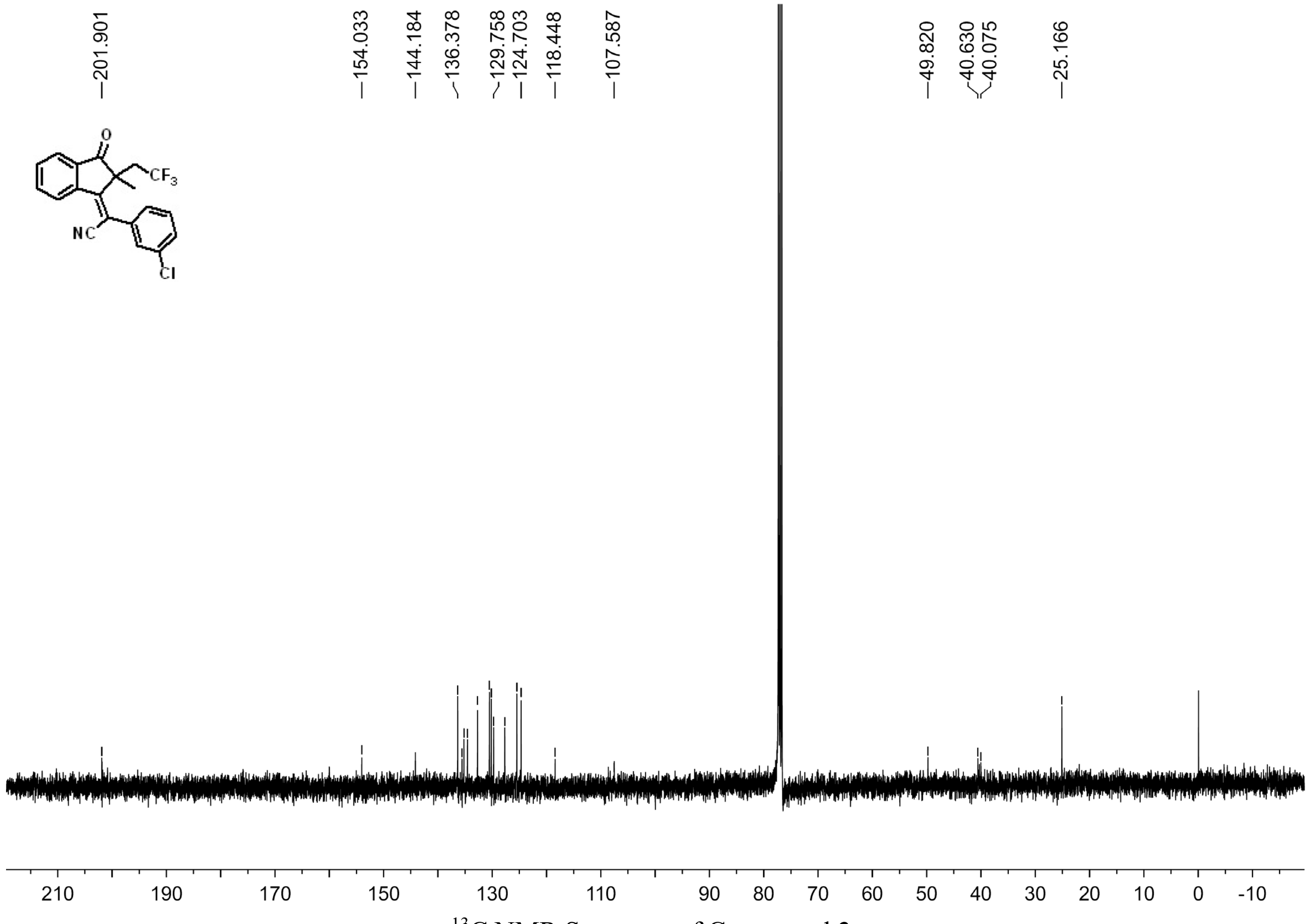

^{13}C NMR Spectrum of Compound **3c**

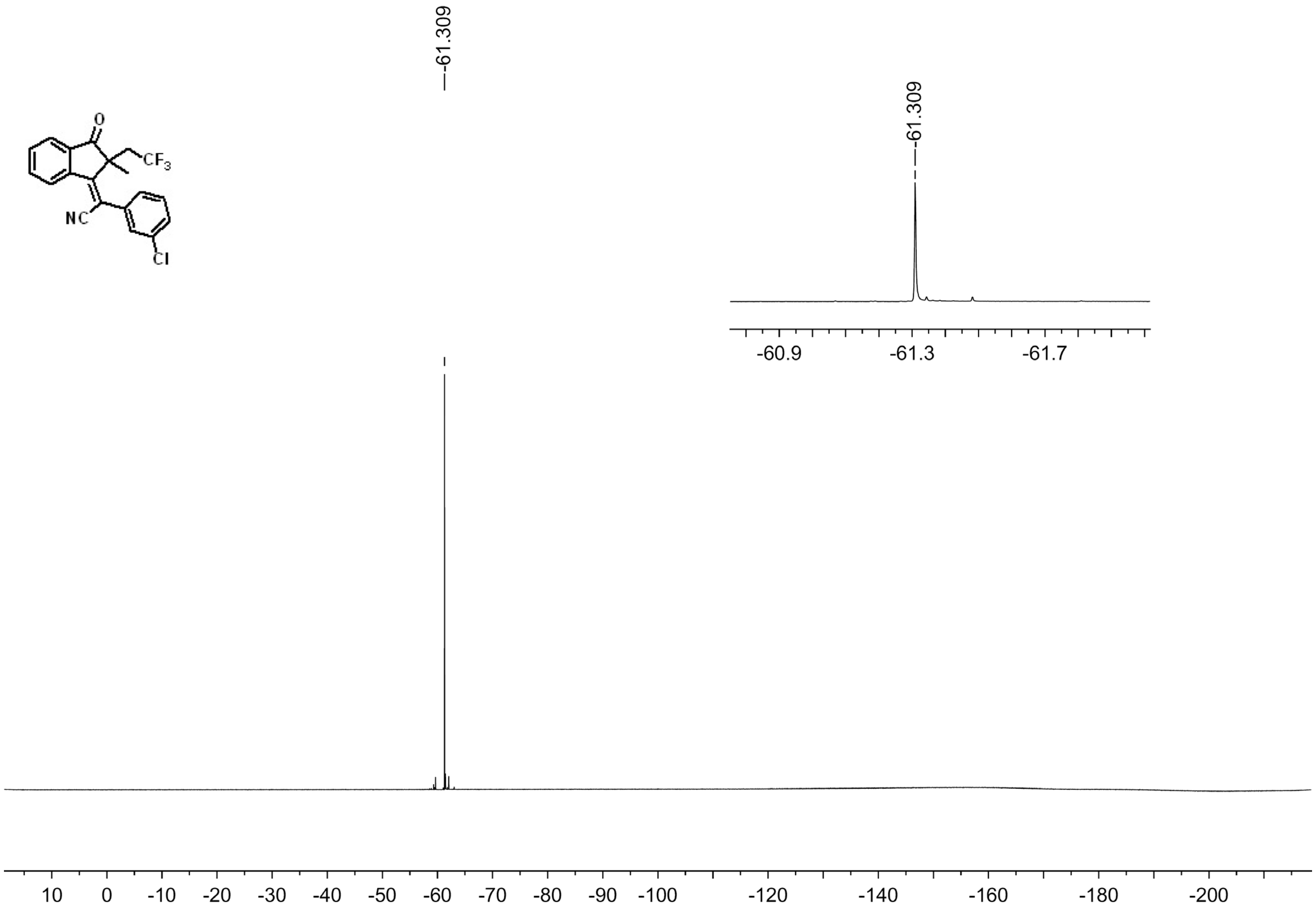

^{19}F NMR Spectrum of Compound **3c**

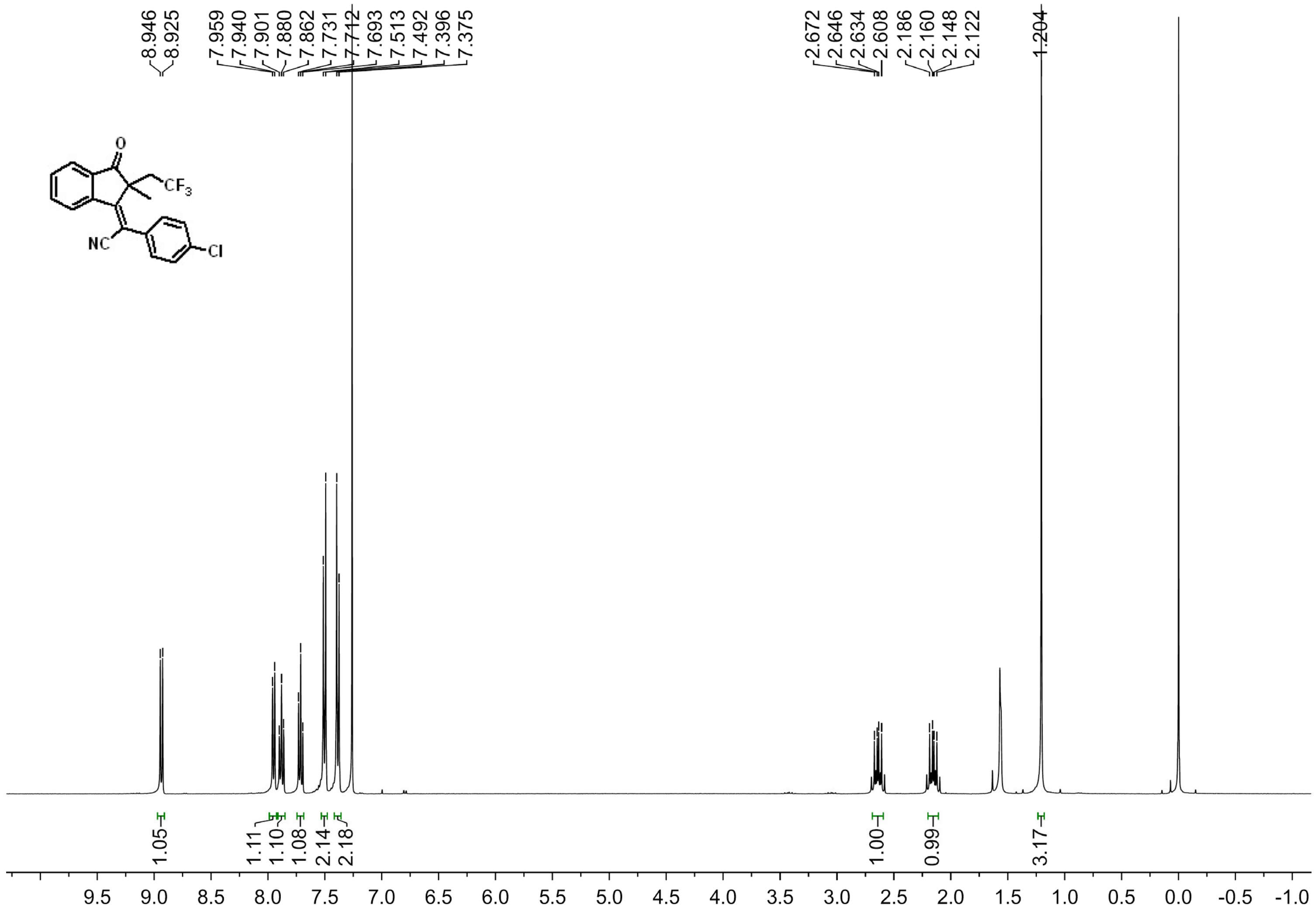

^{1}H NMR Spectrum of Compound **3d**

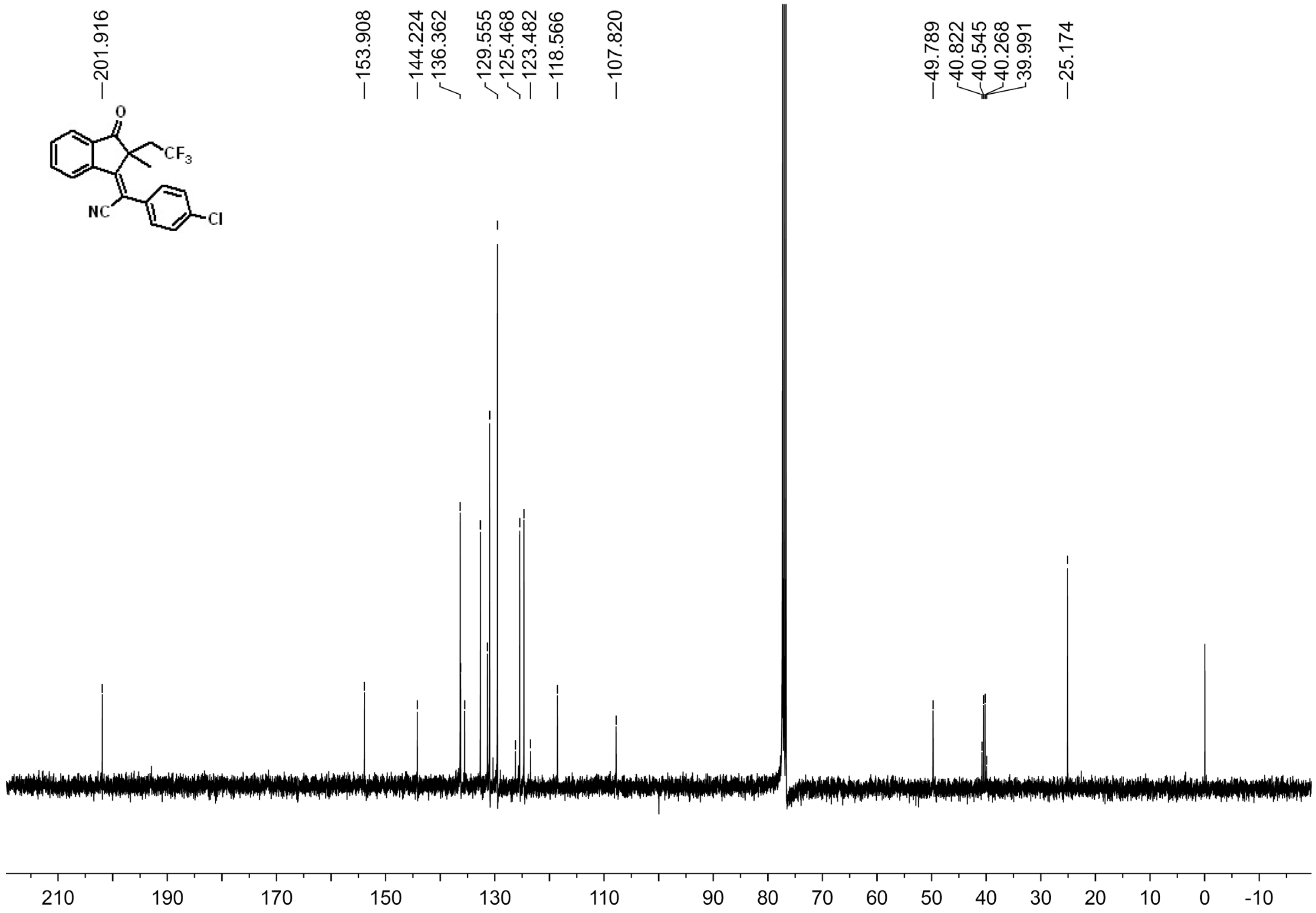

^{13}C NMR Spectrum of Compound **3d**

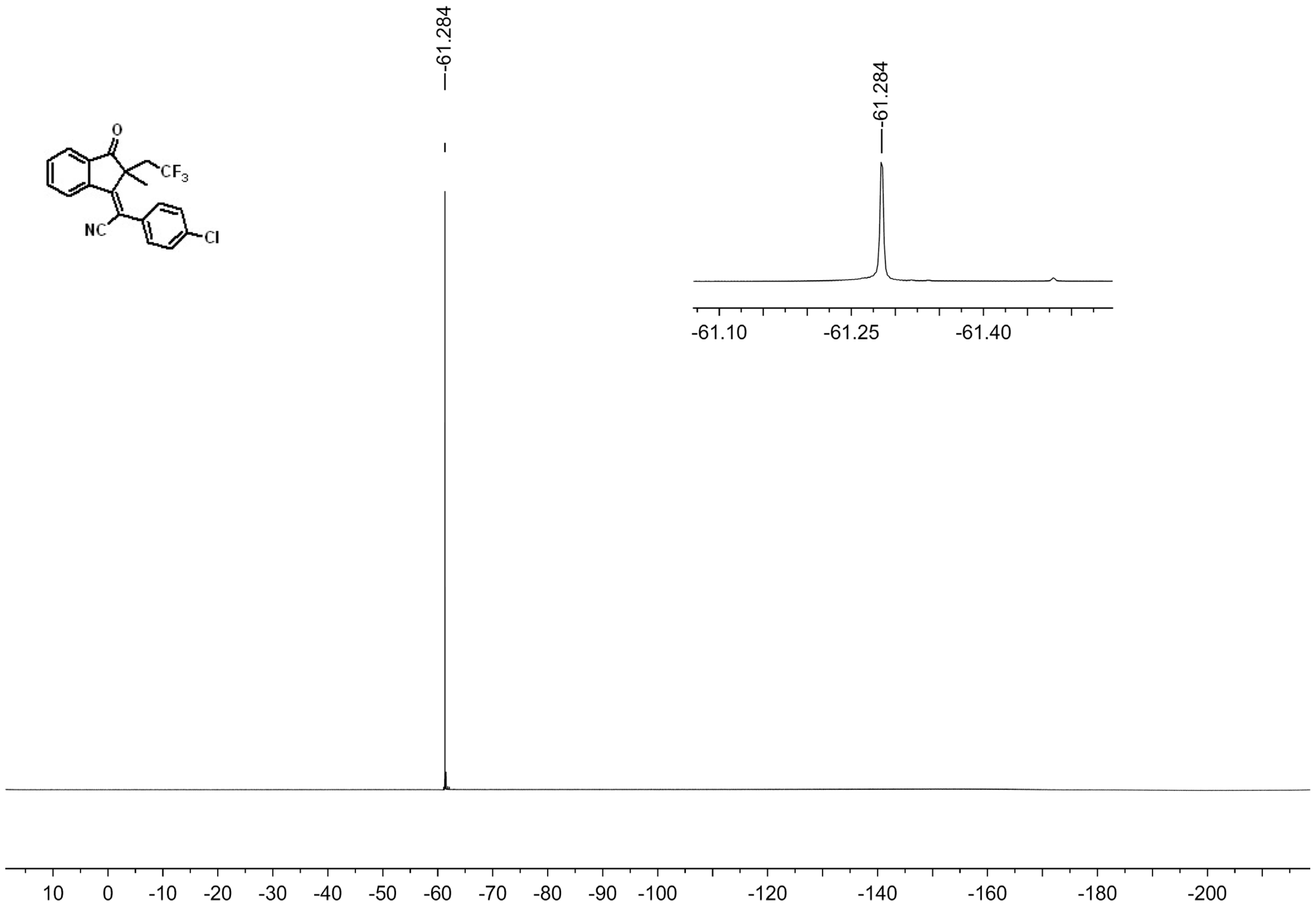

^{19}F NMR Spectrum of Compound **3d**

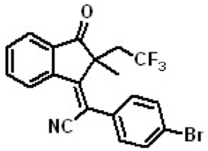

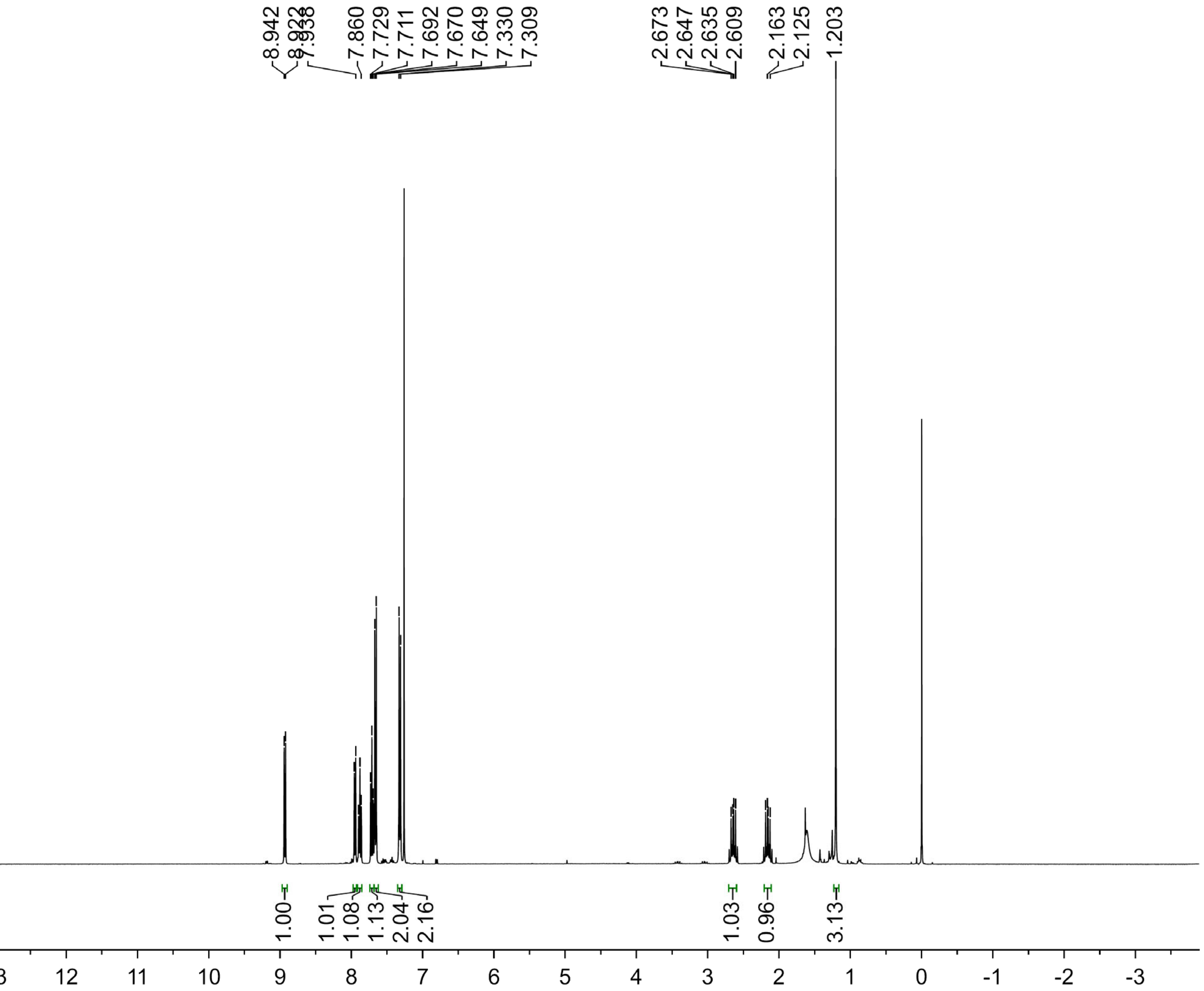

^{1}H NMR Spectrum of Compound **3e**

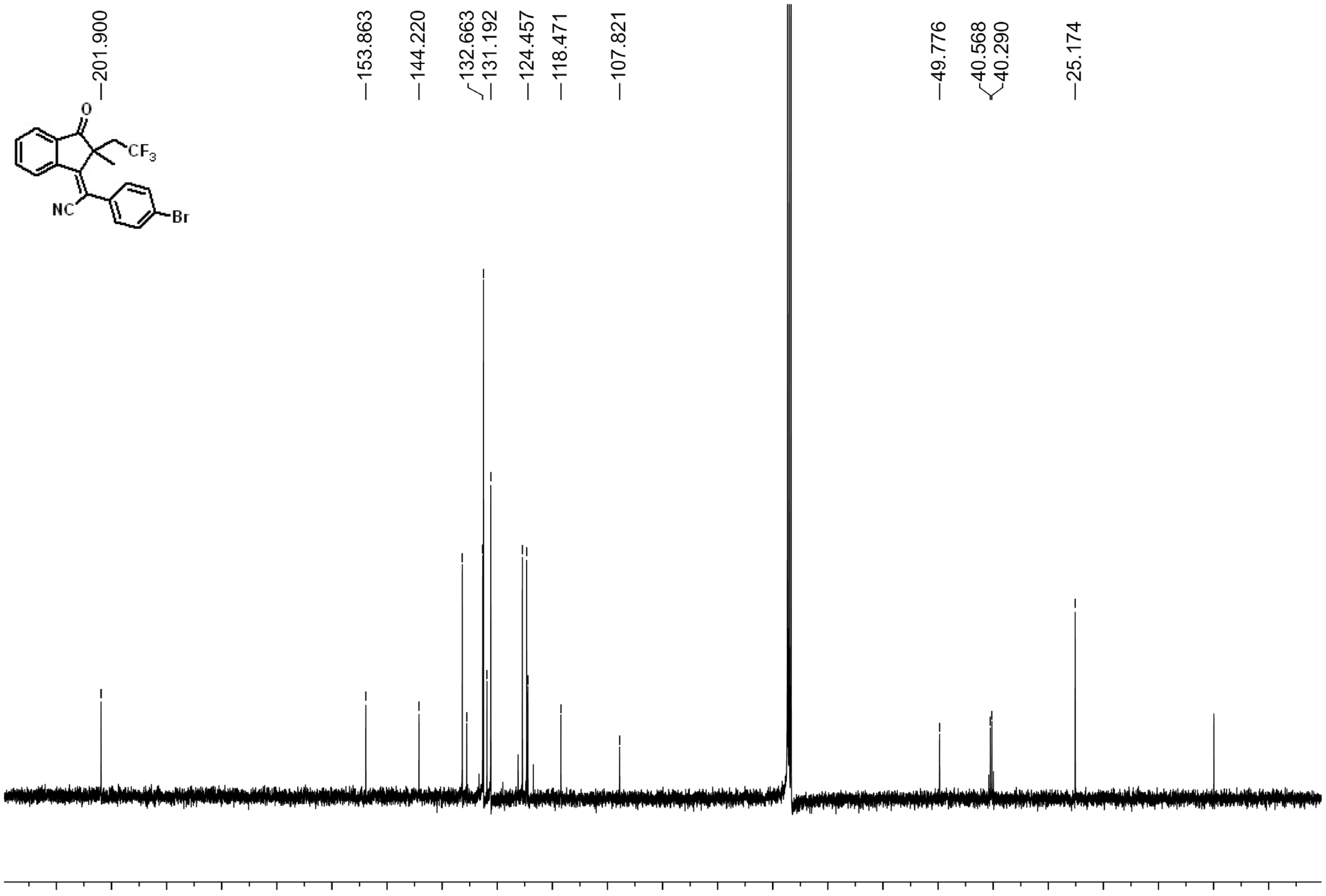

^{13}C NMR Spectrum of Compound **3e**

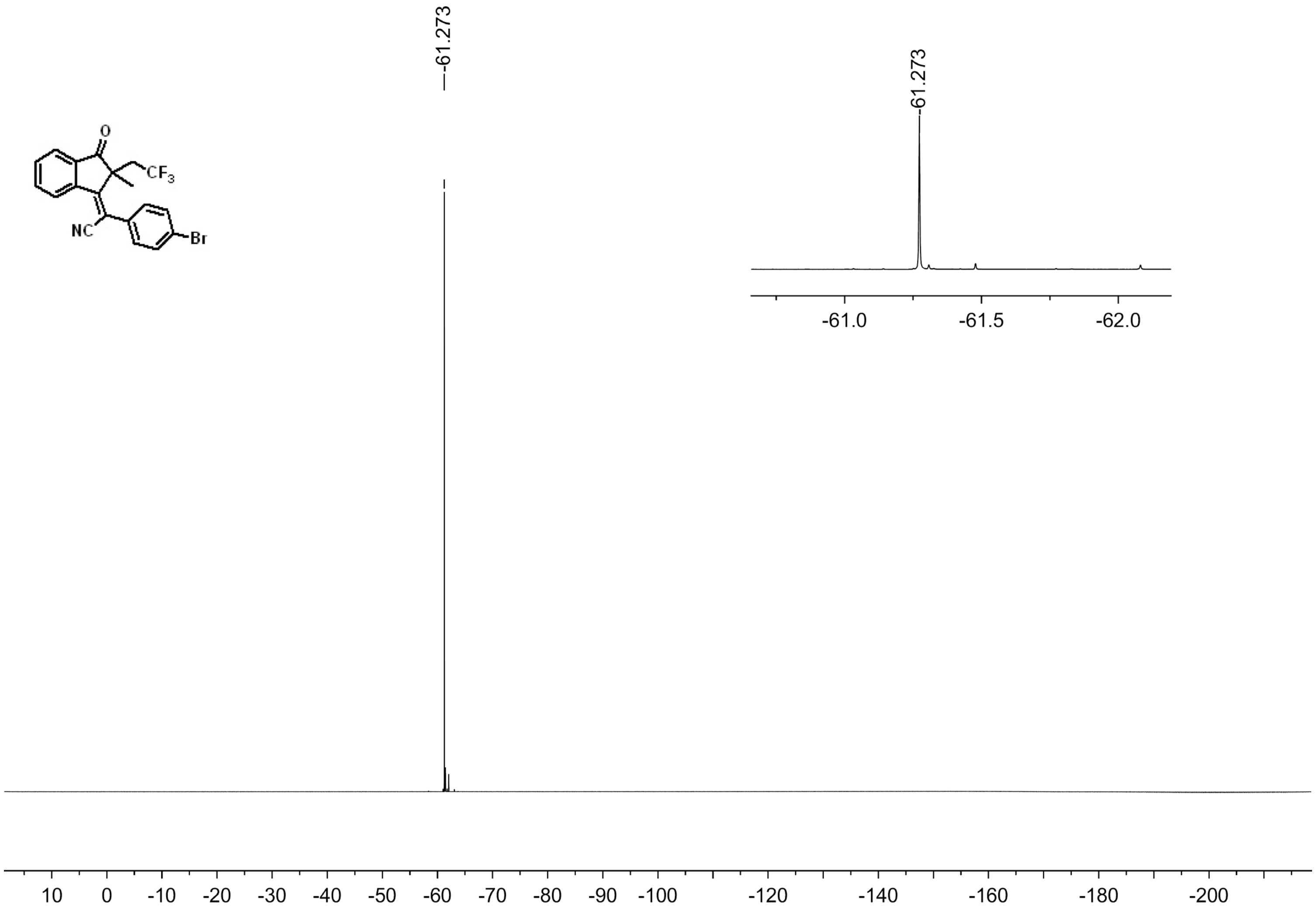

^{19}F NMR Spectrum of Compound **3e**

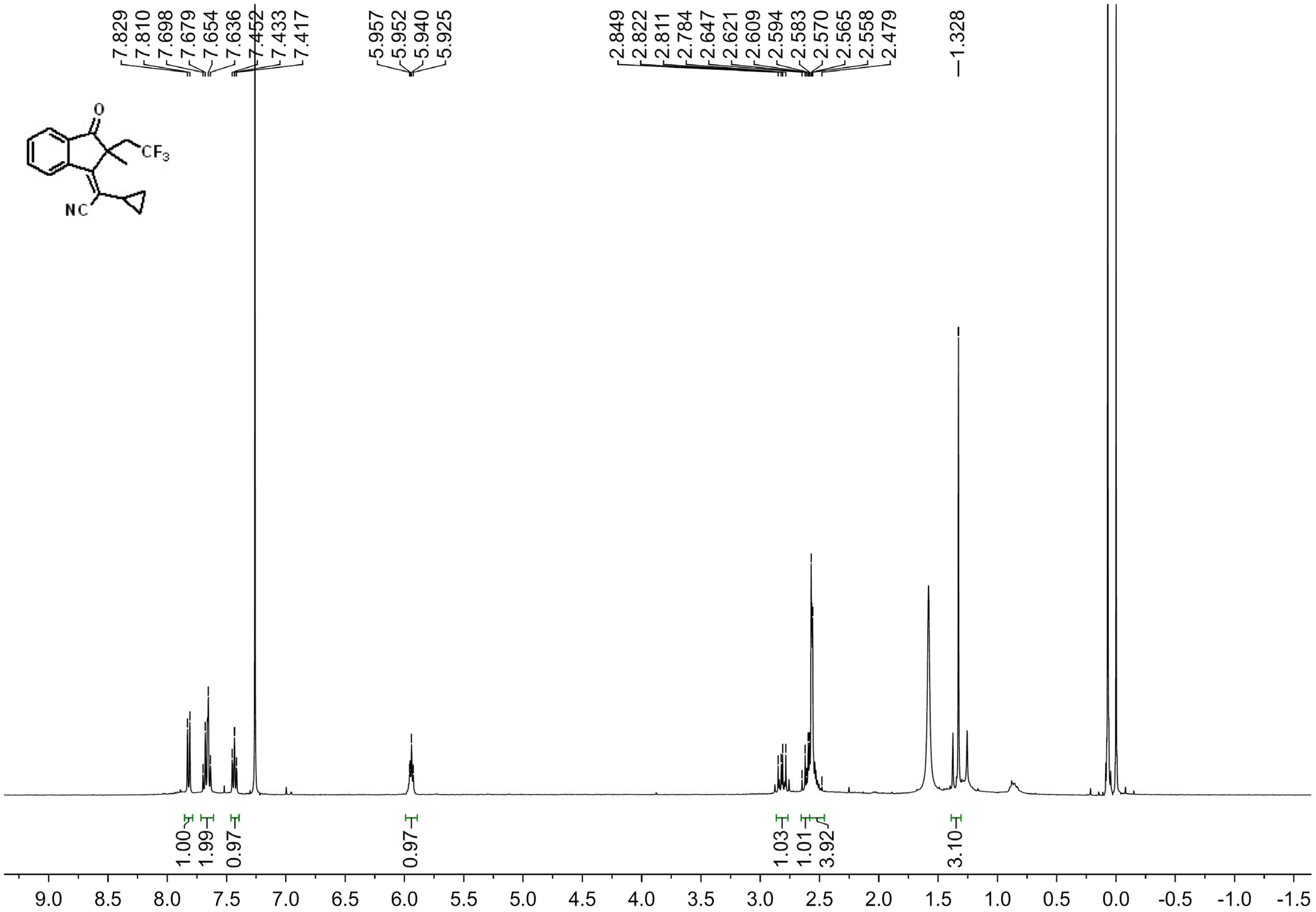

^{1}H NMR Spectrum of Compound **3f**

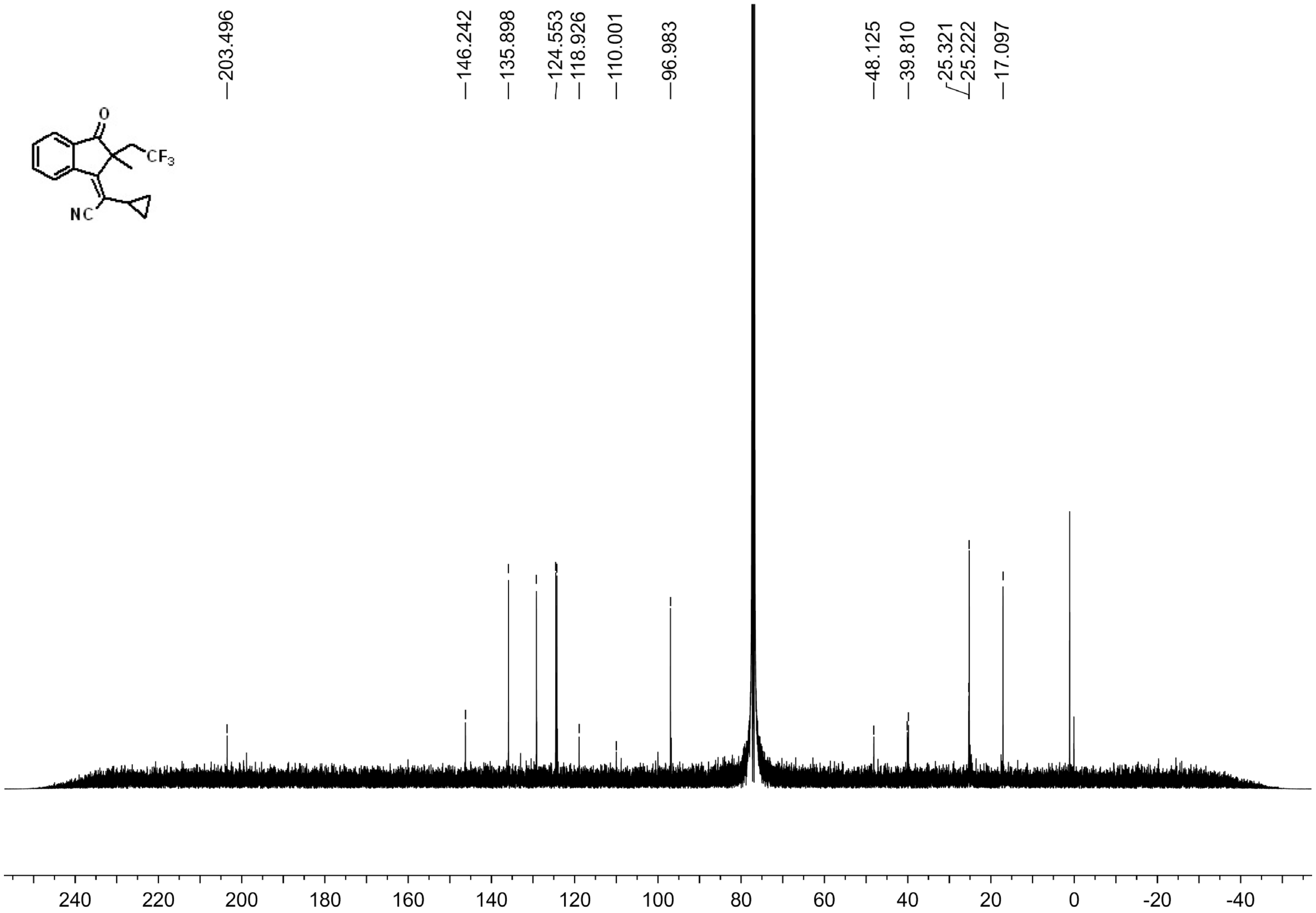

^{13}C NMR Spectrum of Compound **3f**

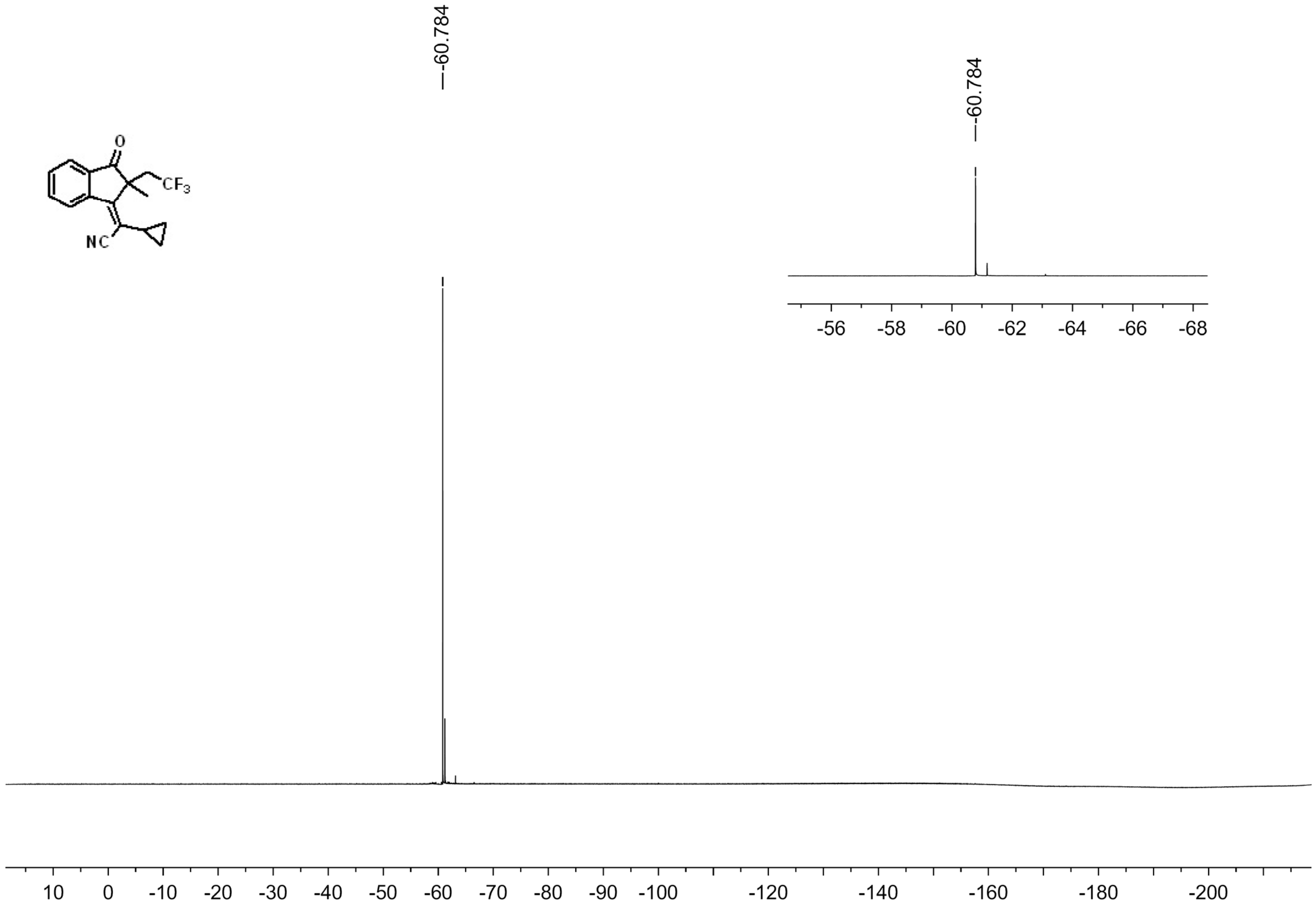

^{19}F NMR Spectrum of Compound **3f**

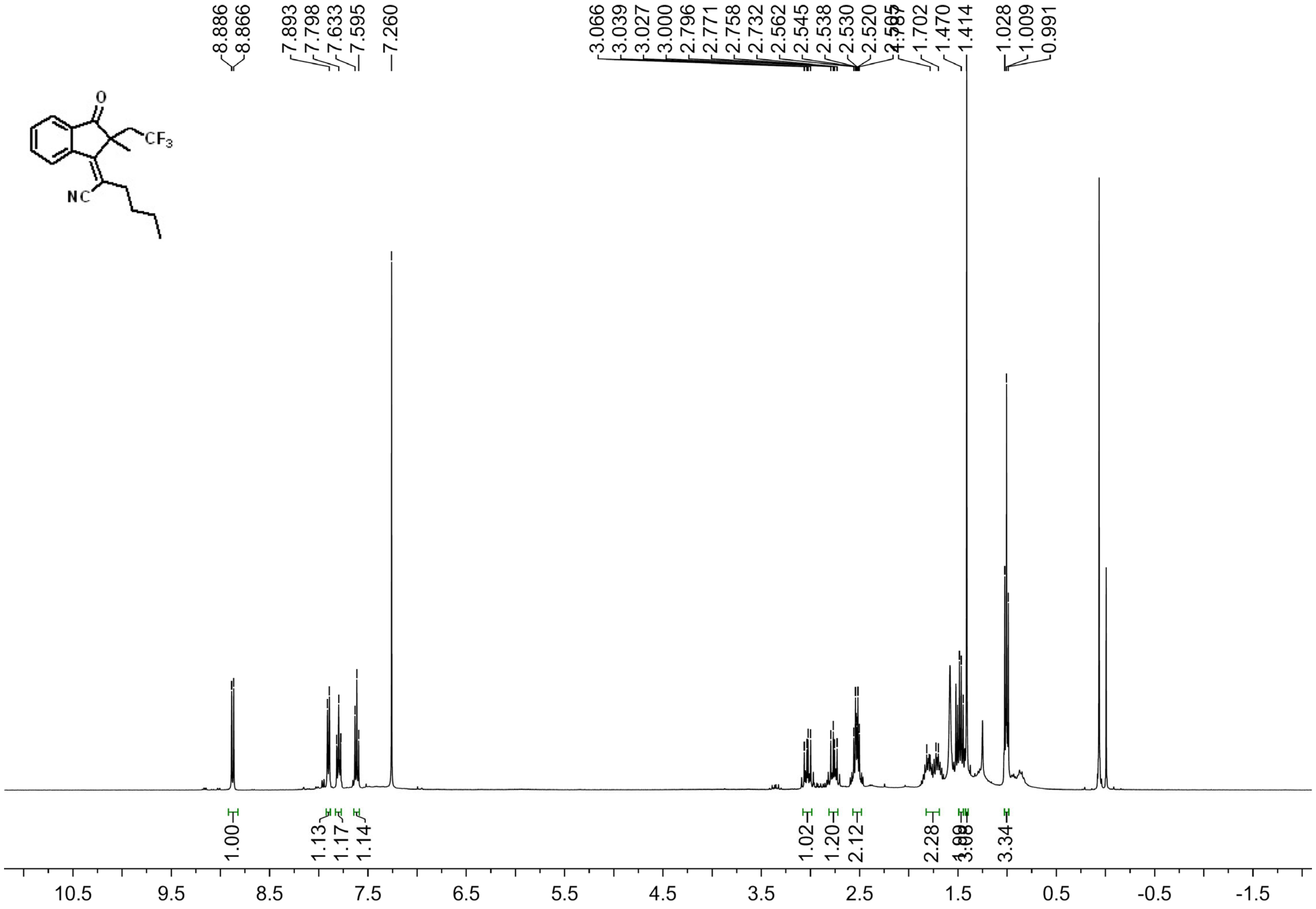

^{1}H NMR Spectrum of Compound **3g**

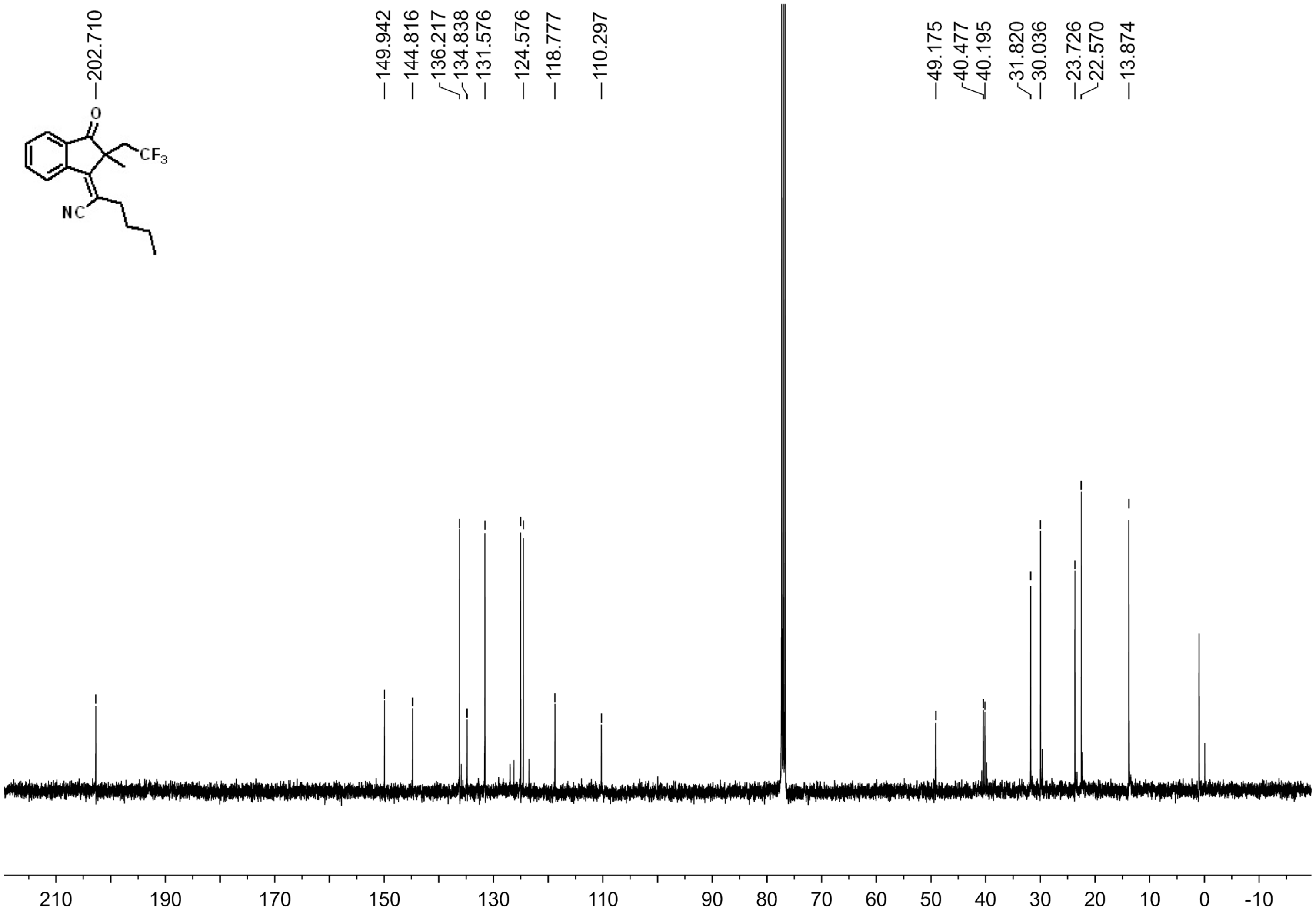

^{13}C NMR Spectrum of Compound 3**g**

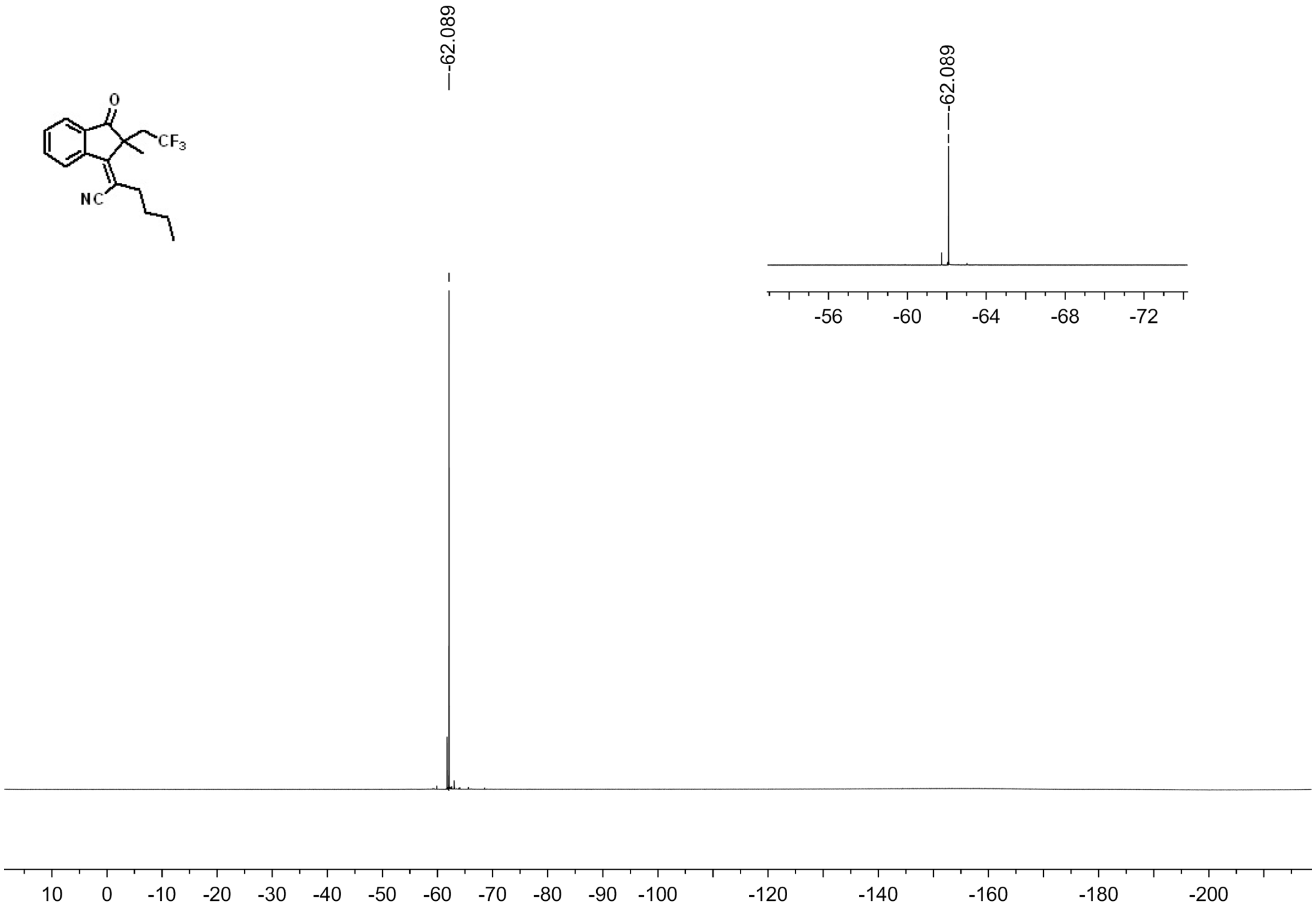

^{19}F NMR Spectrum of Compound **3g**

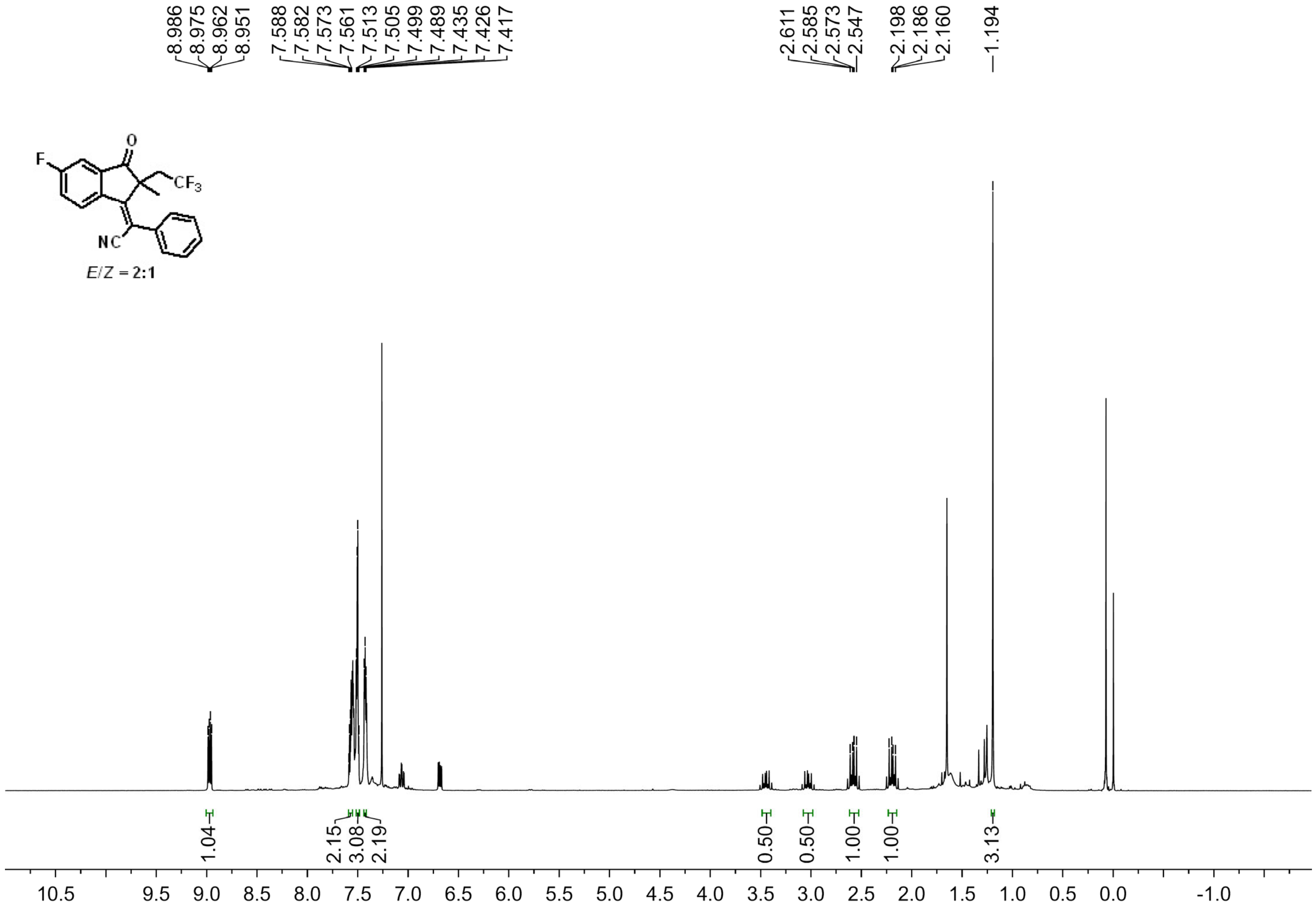

^{1}H NMR Spectrum of Compound **3h**

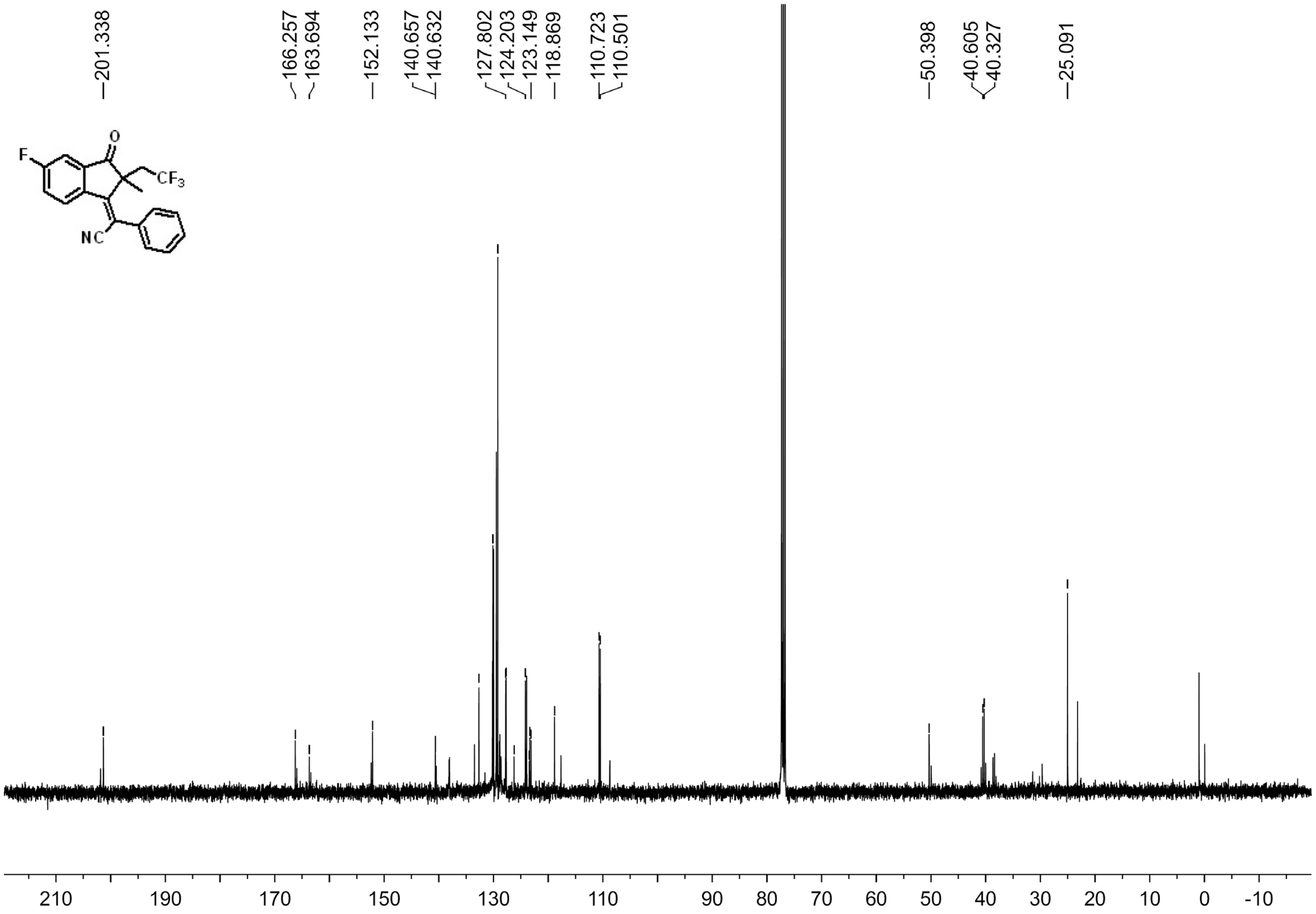

^{13}C NMR Spectrum of Compound **3h**

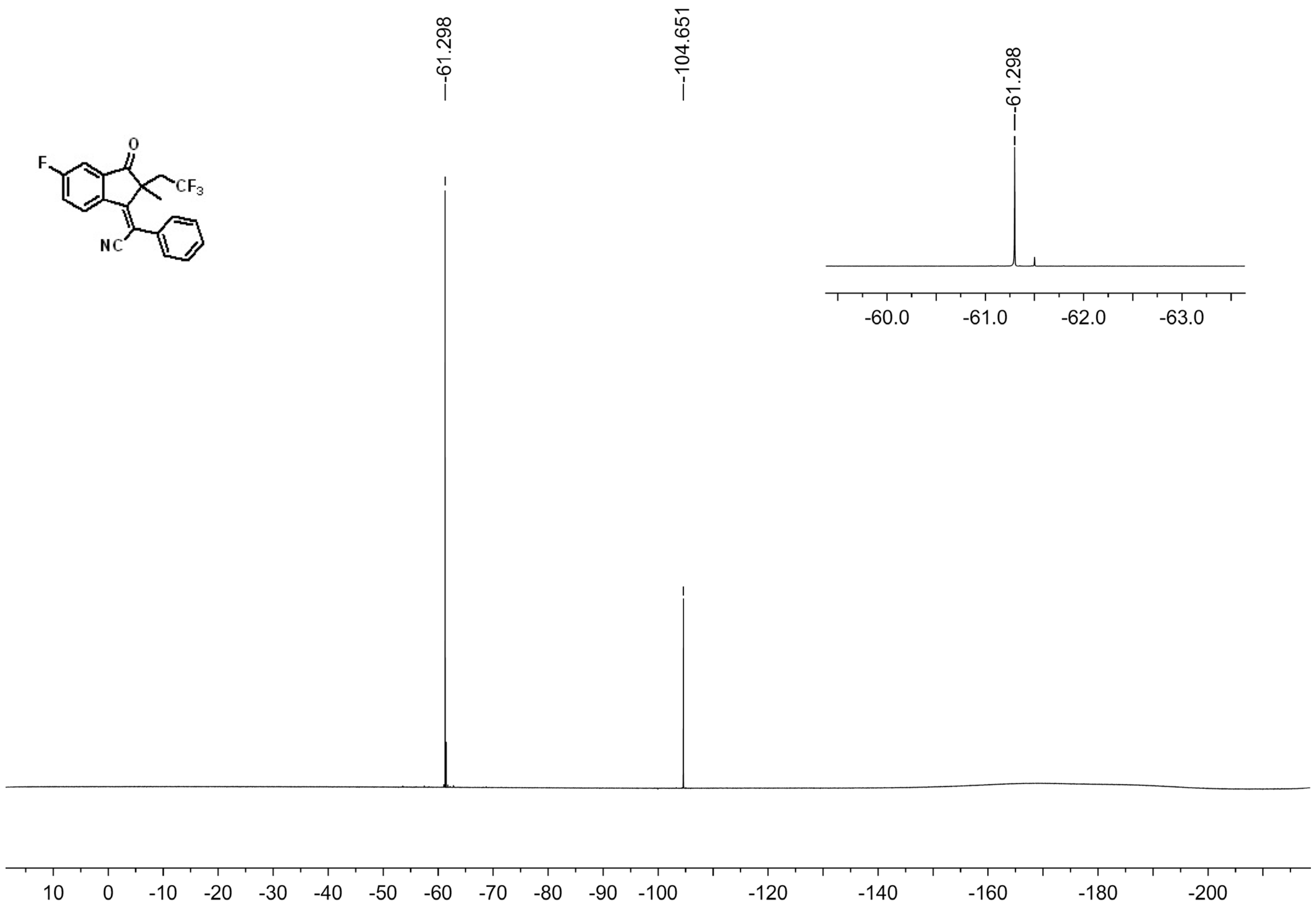

^{19}F NMR Spectrum of Compound **3h**

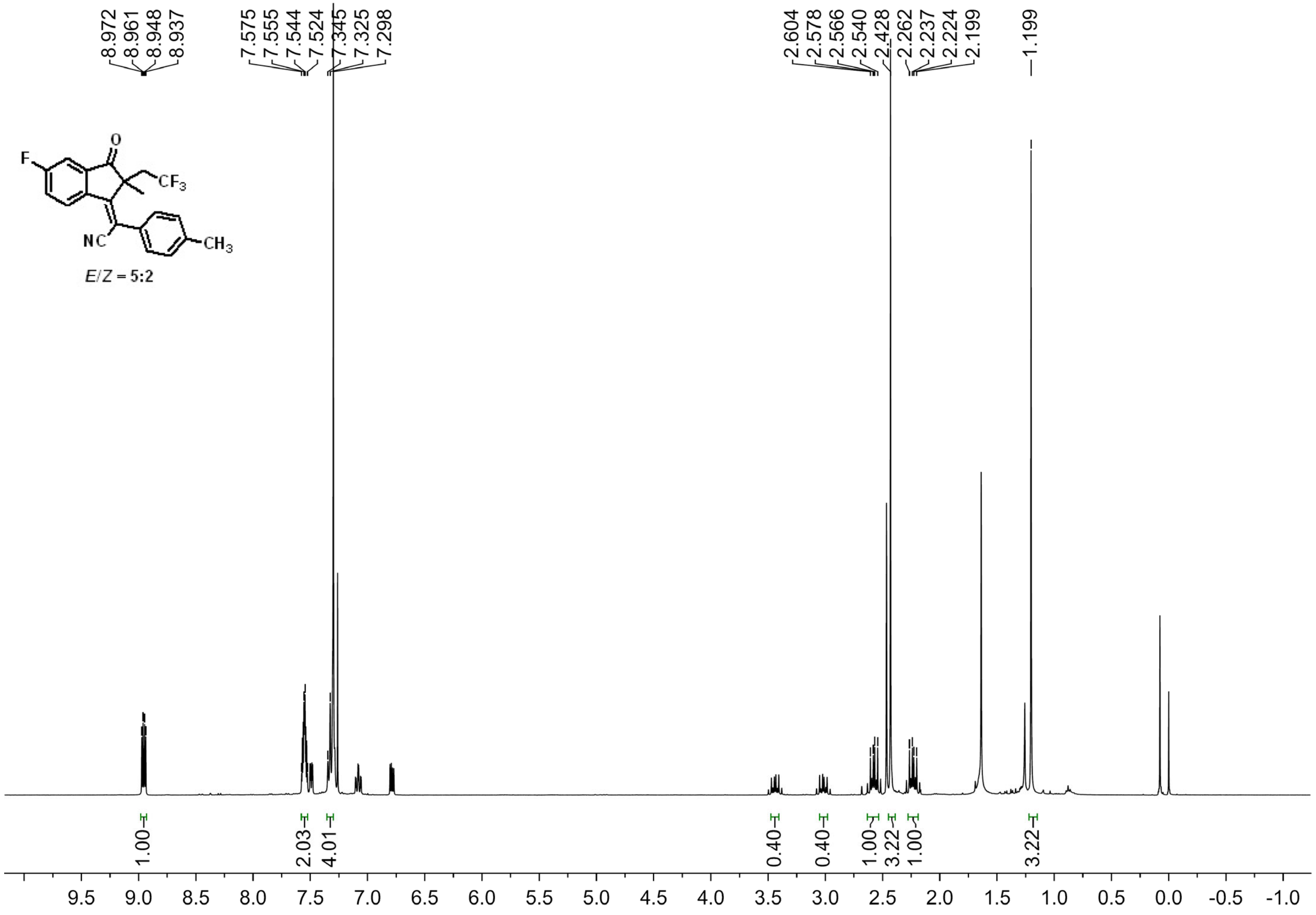

^{1}H NMR Spectrum of Compound **3i**

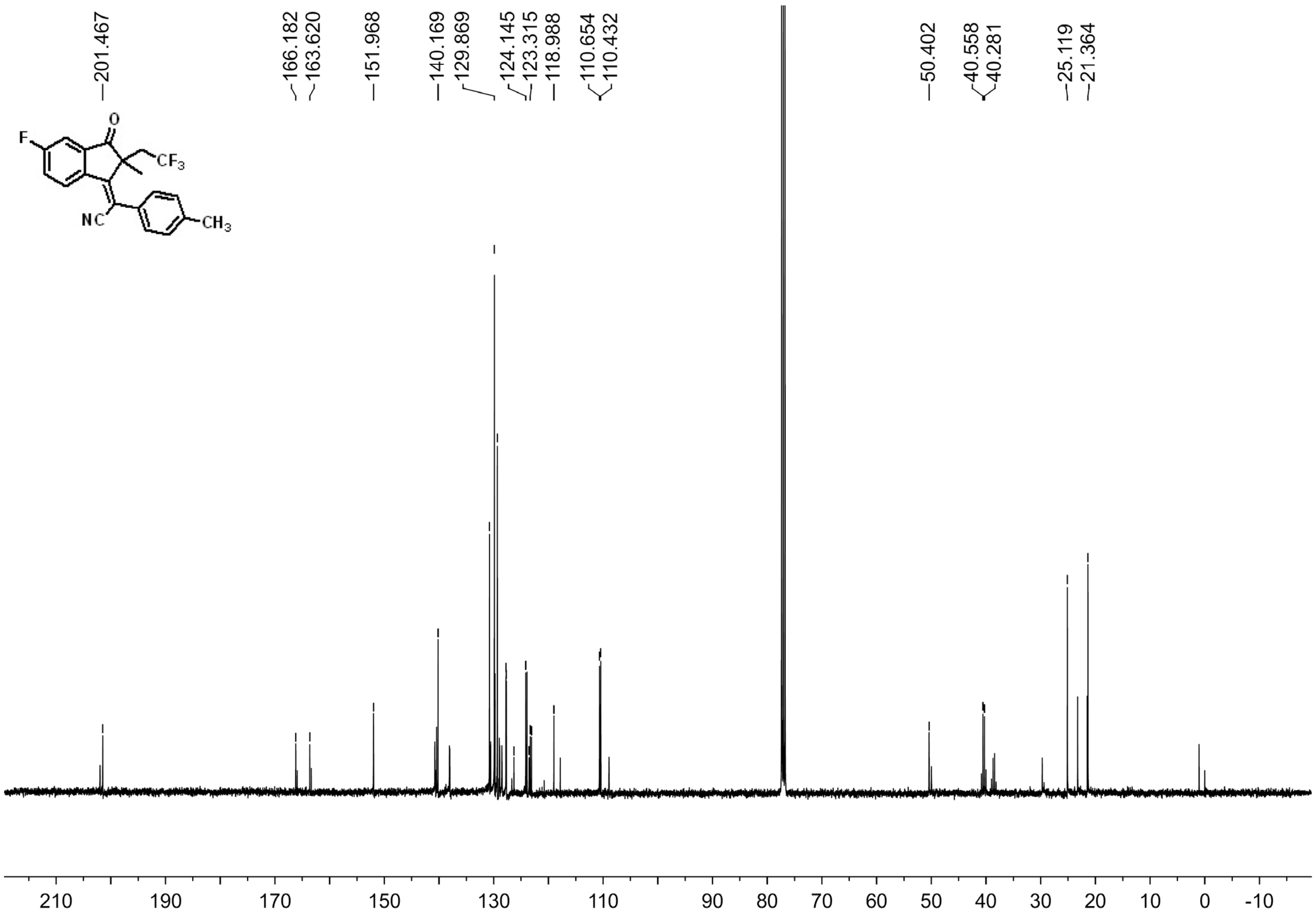

^{13}C NMR Spectrum of Compound **3i**

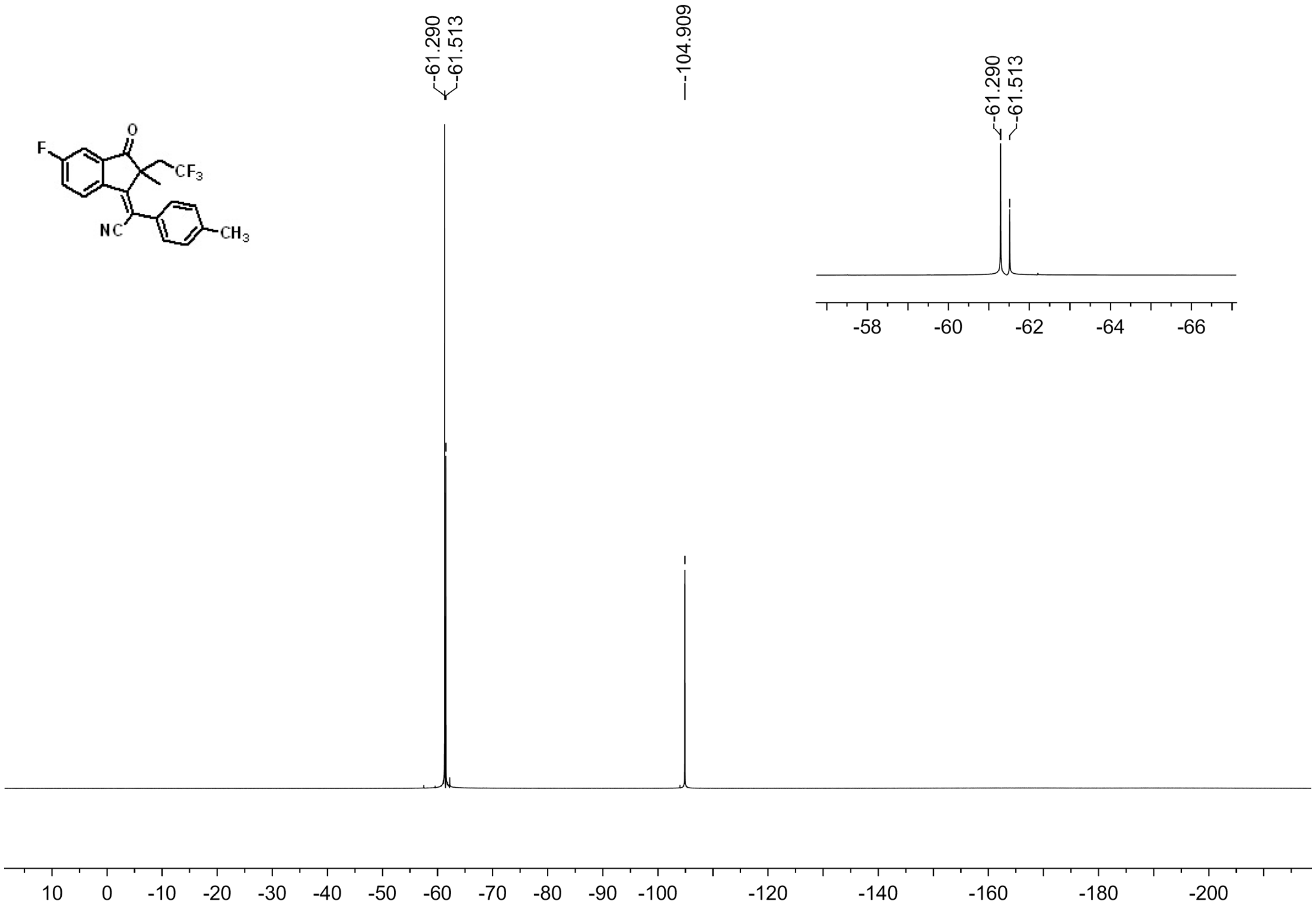

^{19}F NMR Spectrum of Compound **3i**

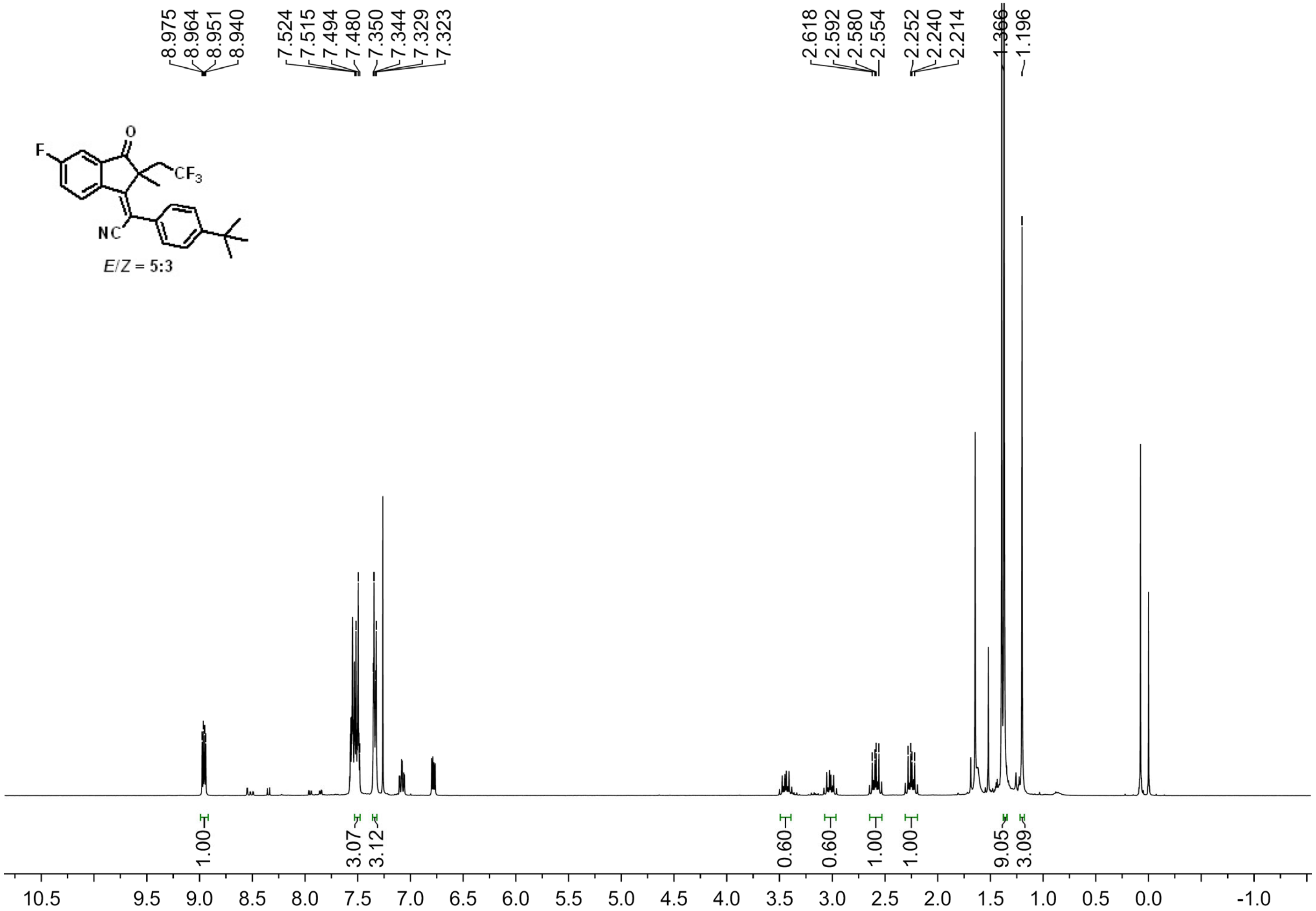

^{1}H NMR Spectrum of Compound **3j**

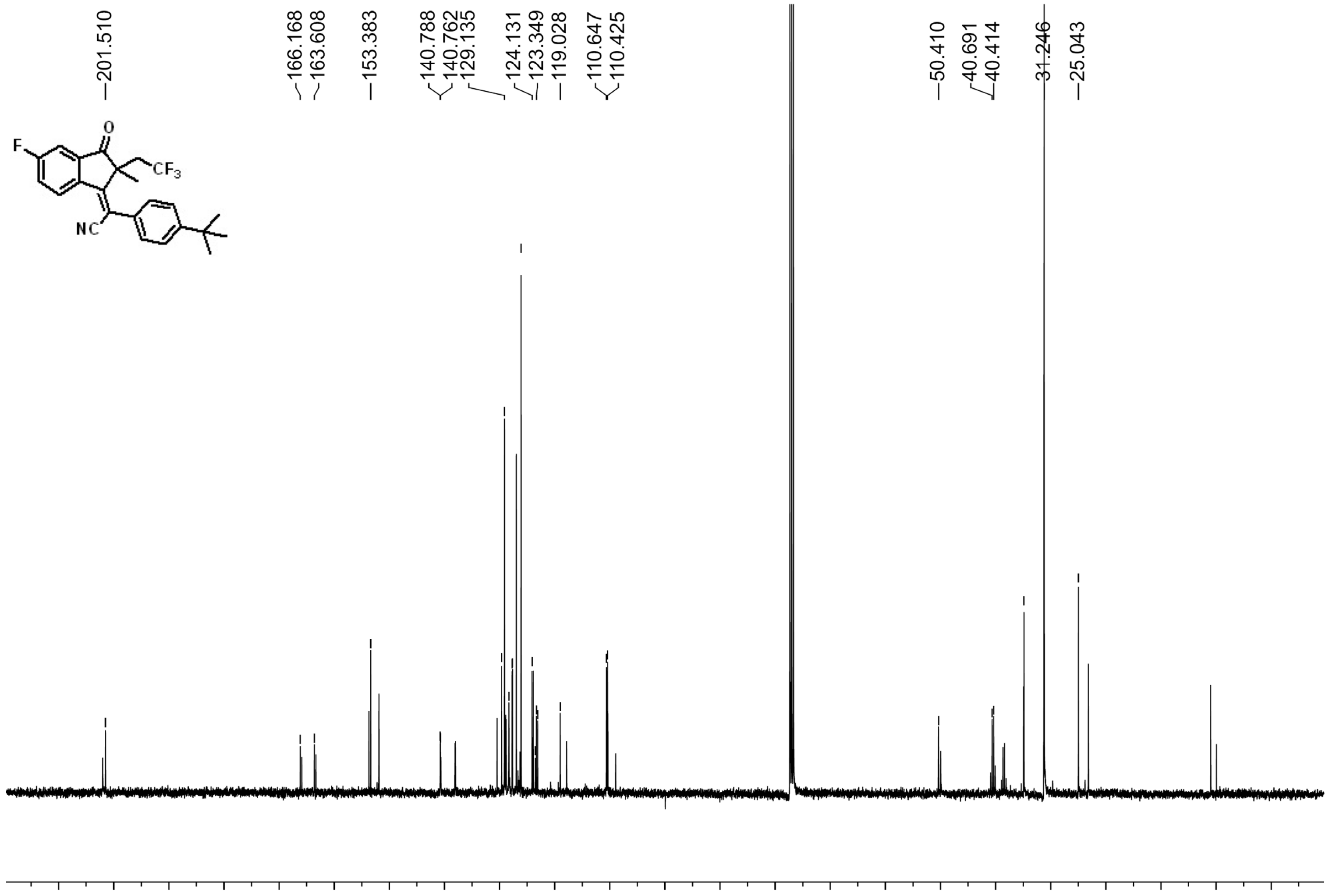

^{13}C NMR Spectrum of Compound **3j**

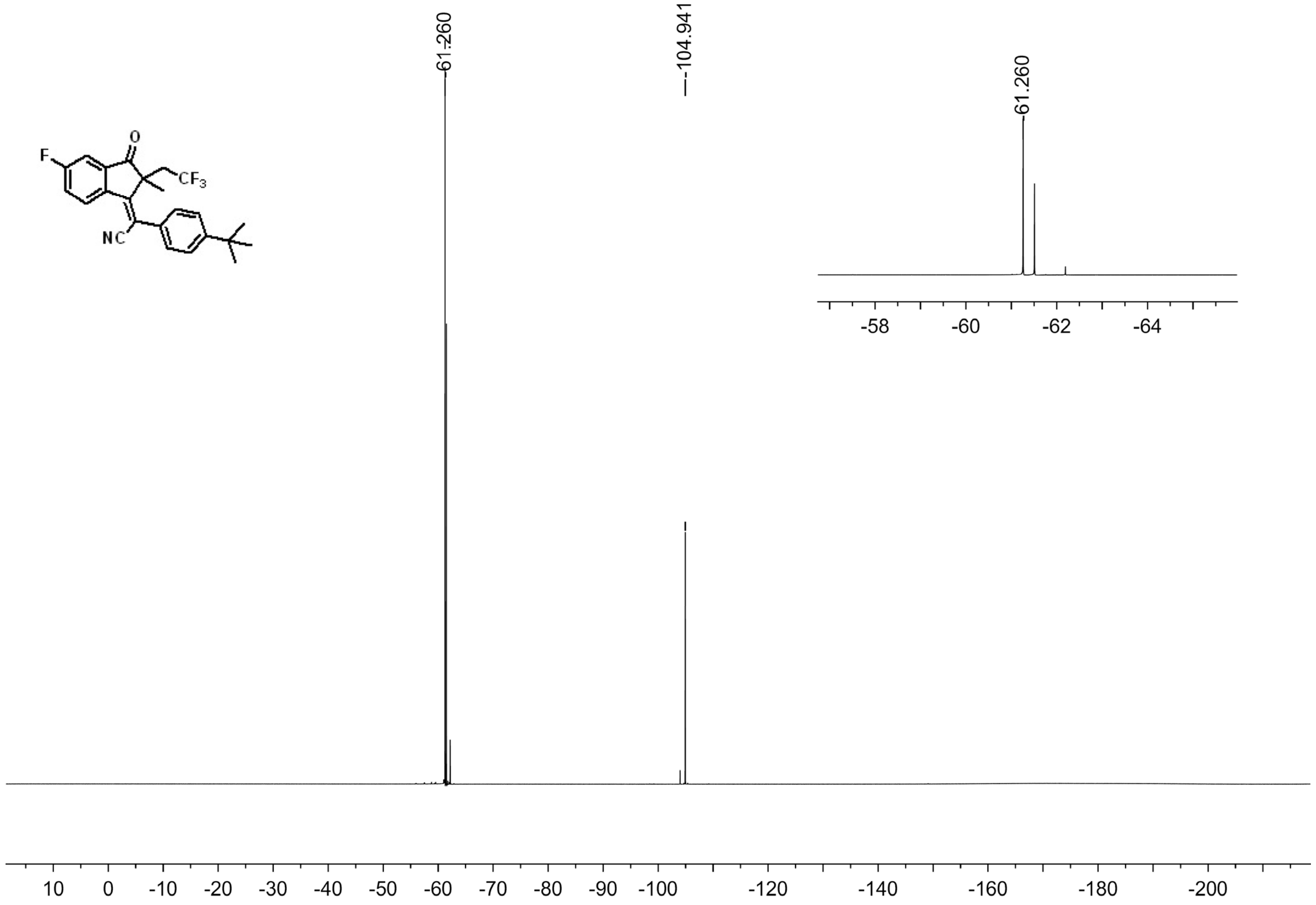

^{19}F NMR Spectrum of Compound **3j**

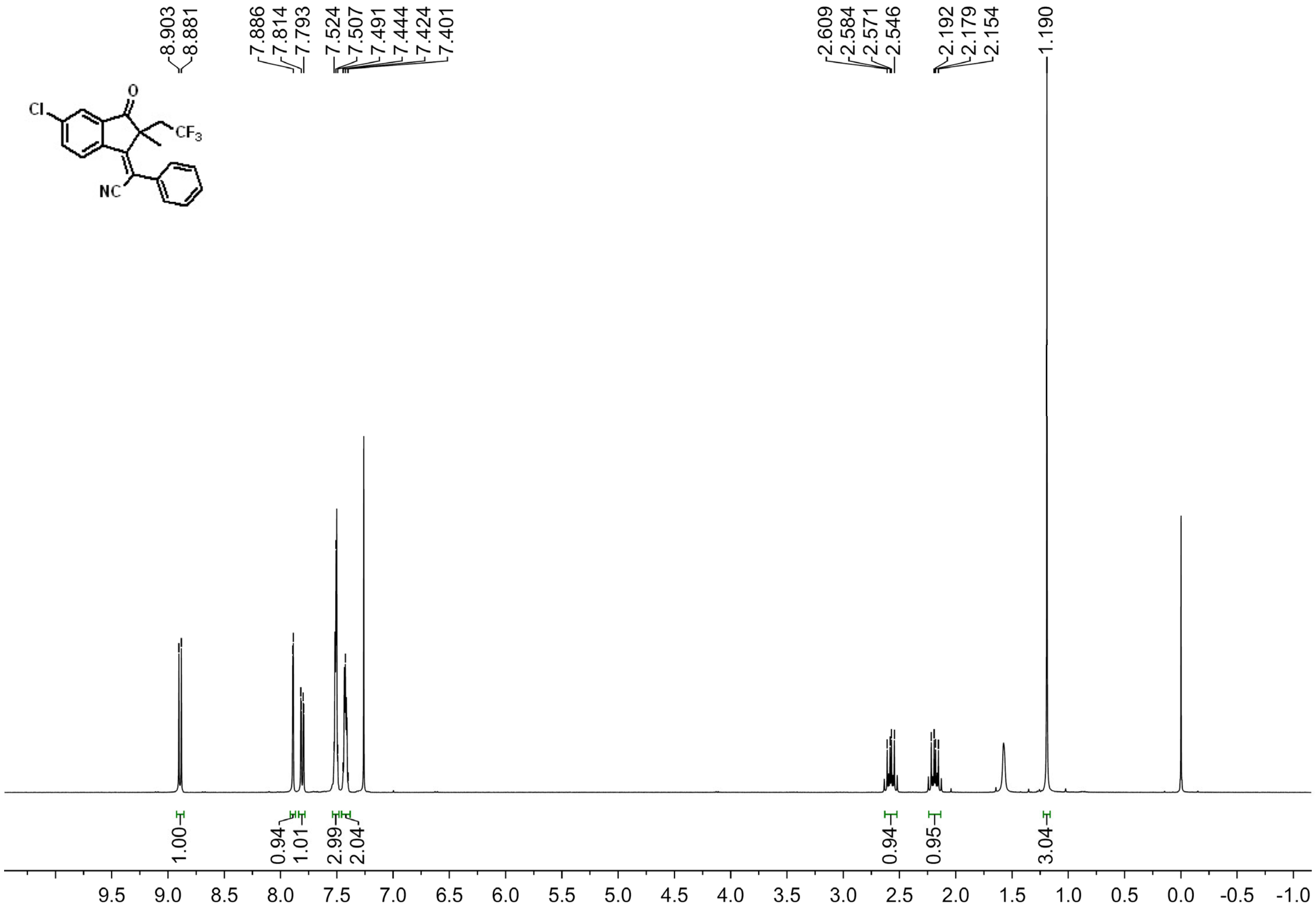

^{1}H NMR Spectrum of Compound **3k**

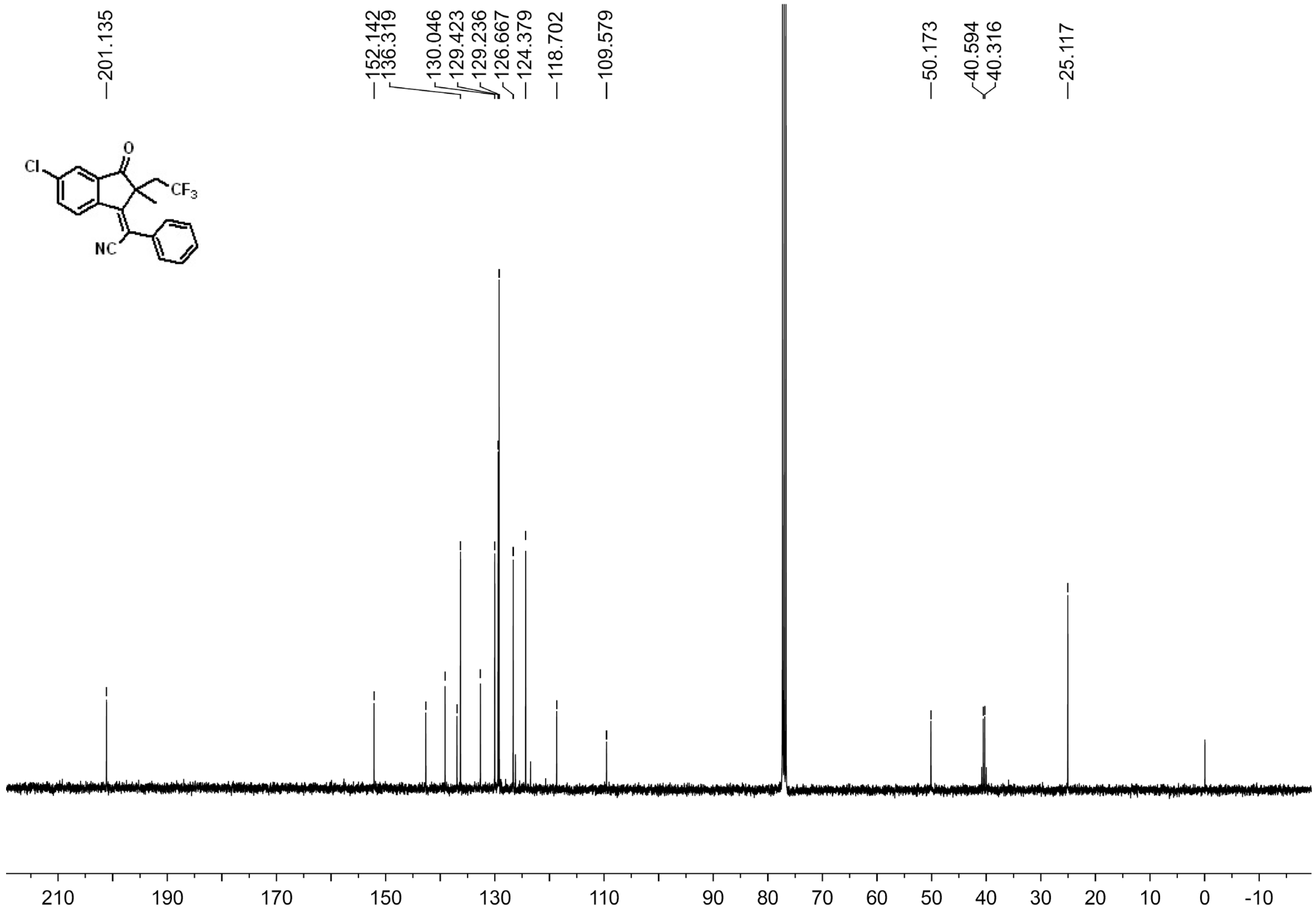

^{13}C NMR Spectrum of Compound **3k**

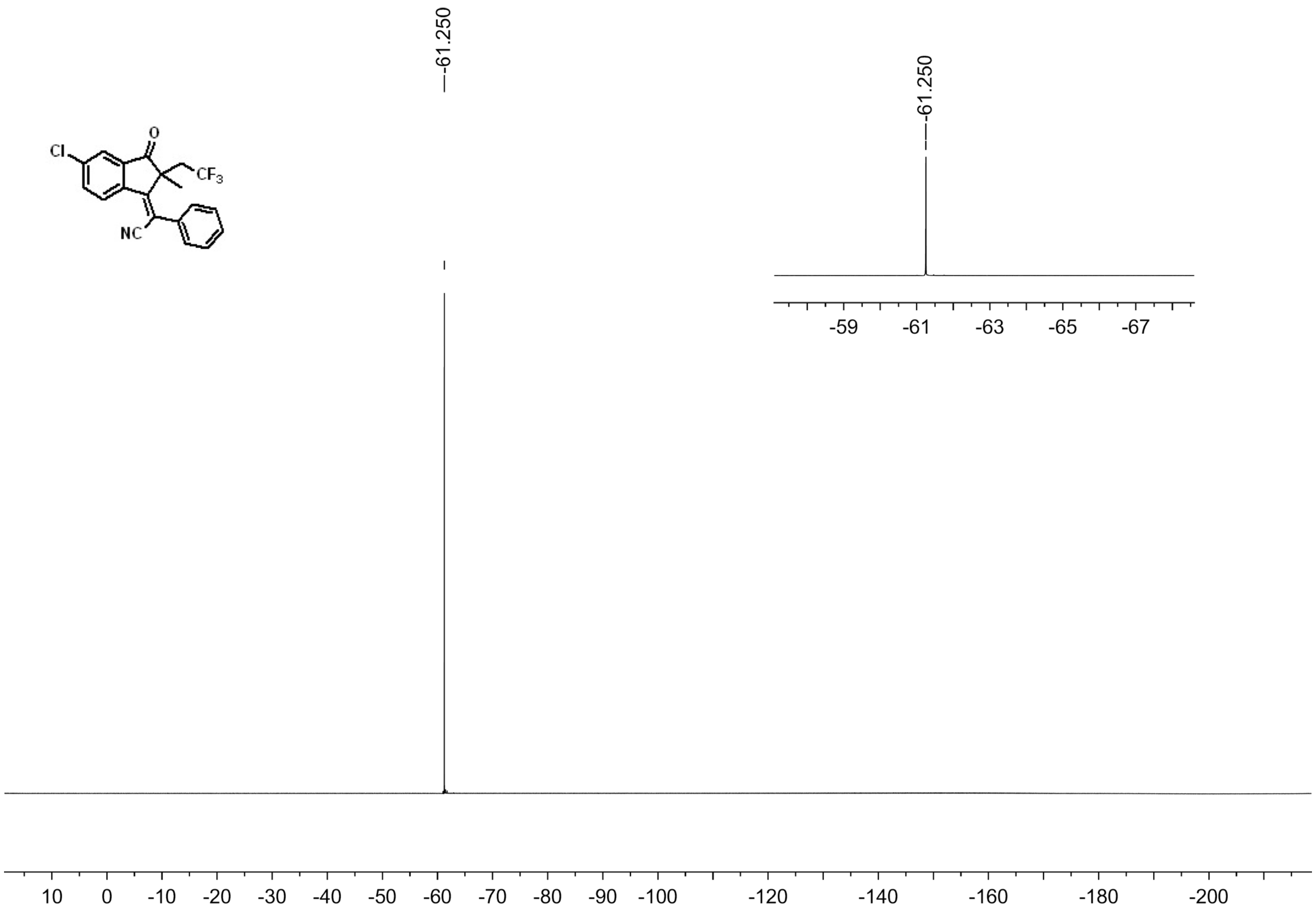

^{19}F NMR Spectrum of Compound **3k**

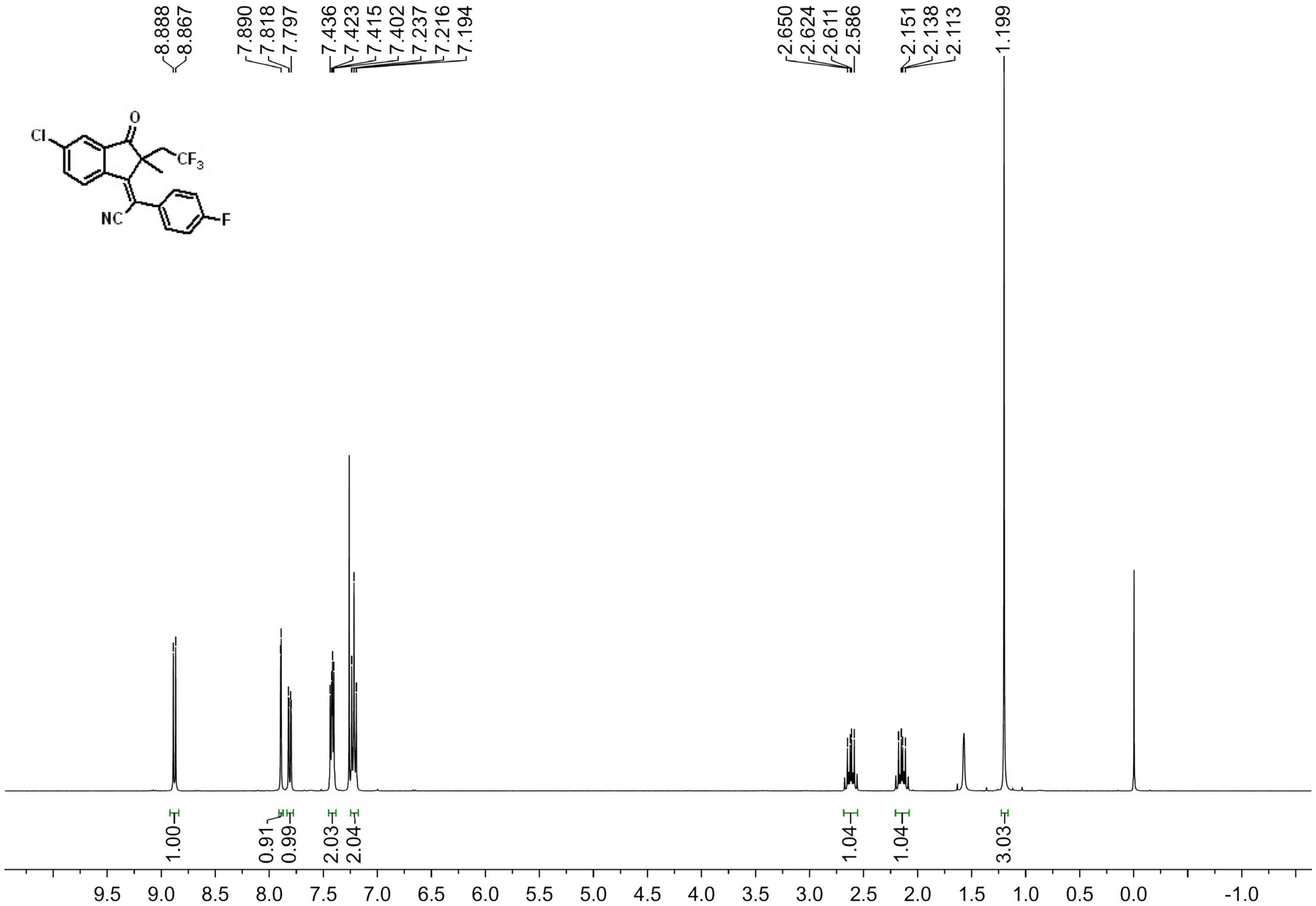

^{1}H NMR Spectrum of Compound **3l**

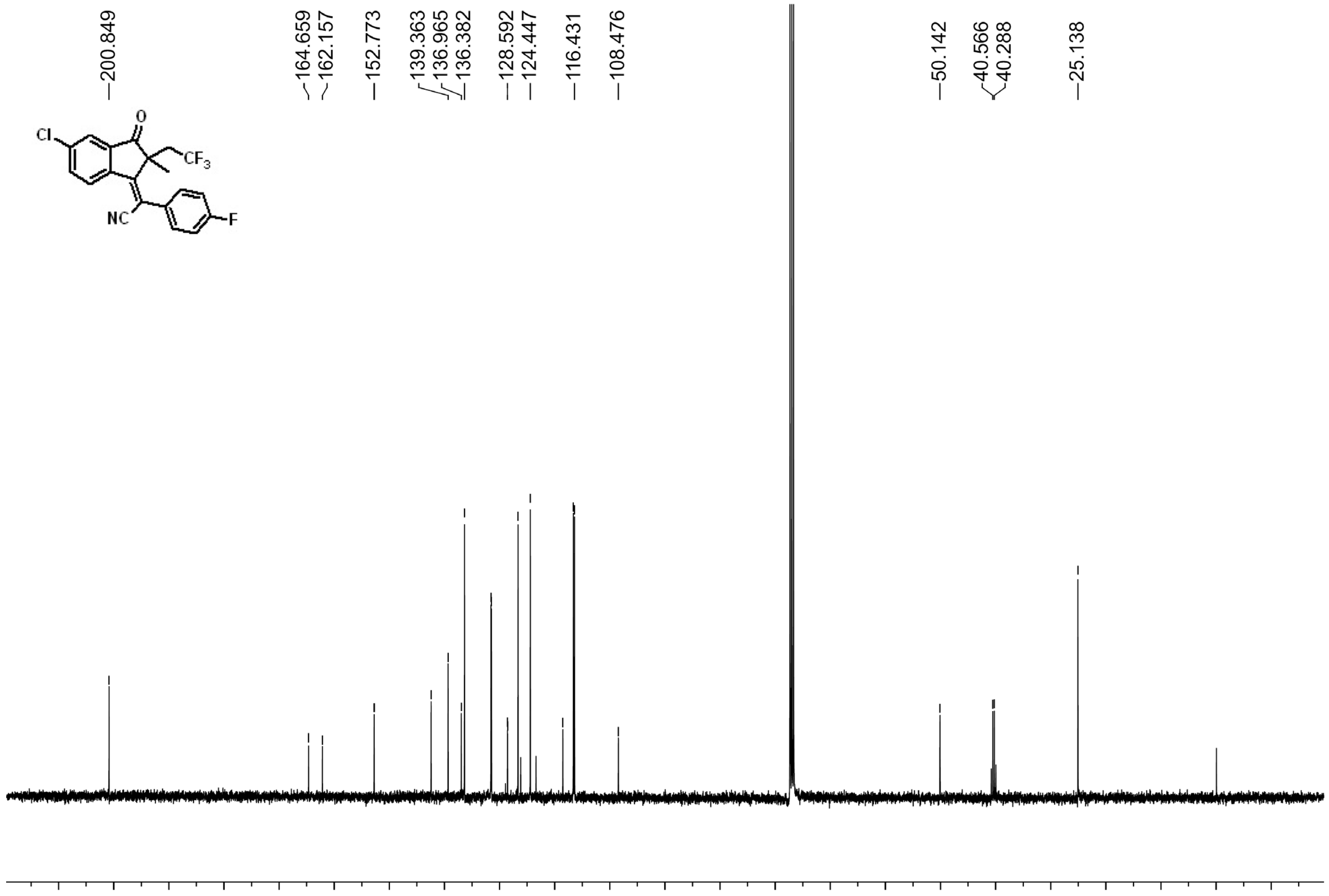

^{13}C NMR Spectrum of Compound **3l**

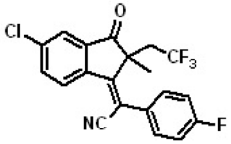

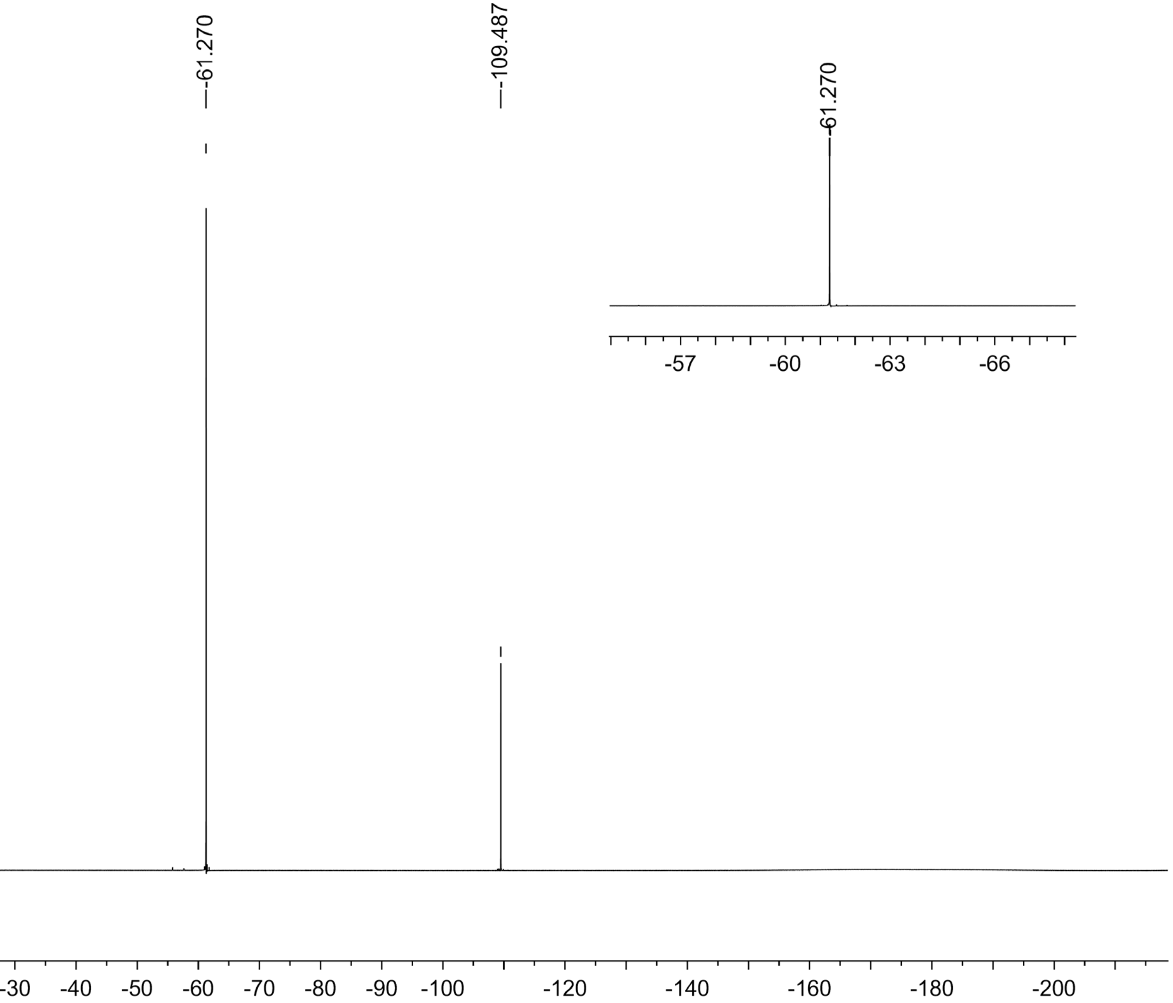

^{19}F NMR Spectrum of Compound **3l**

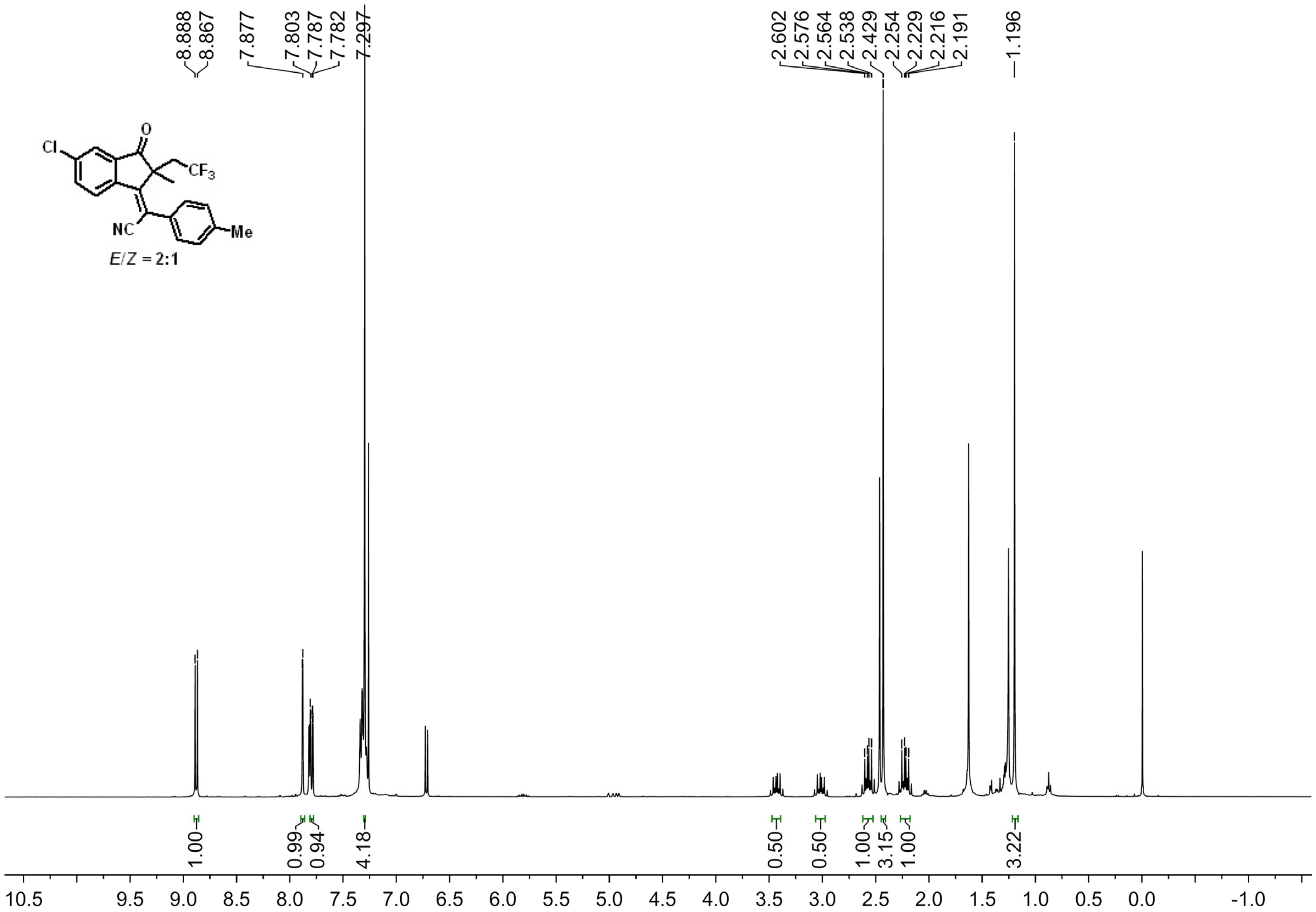

^{1}H NMR Spectrum of Compound **3m**

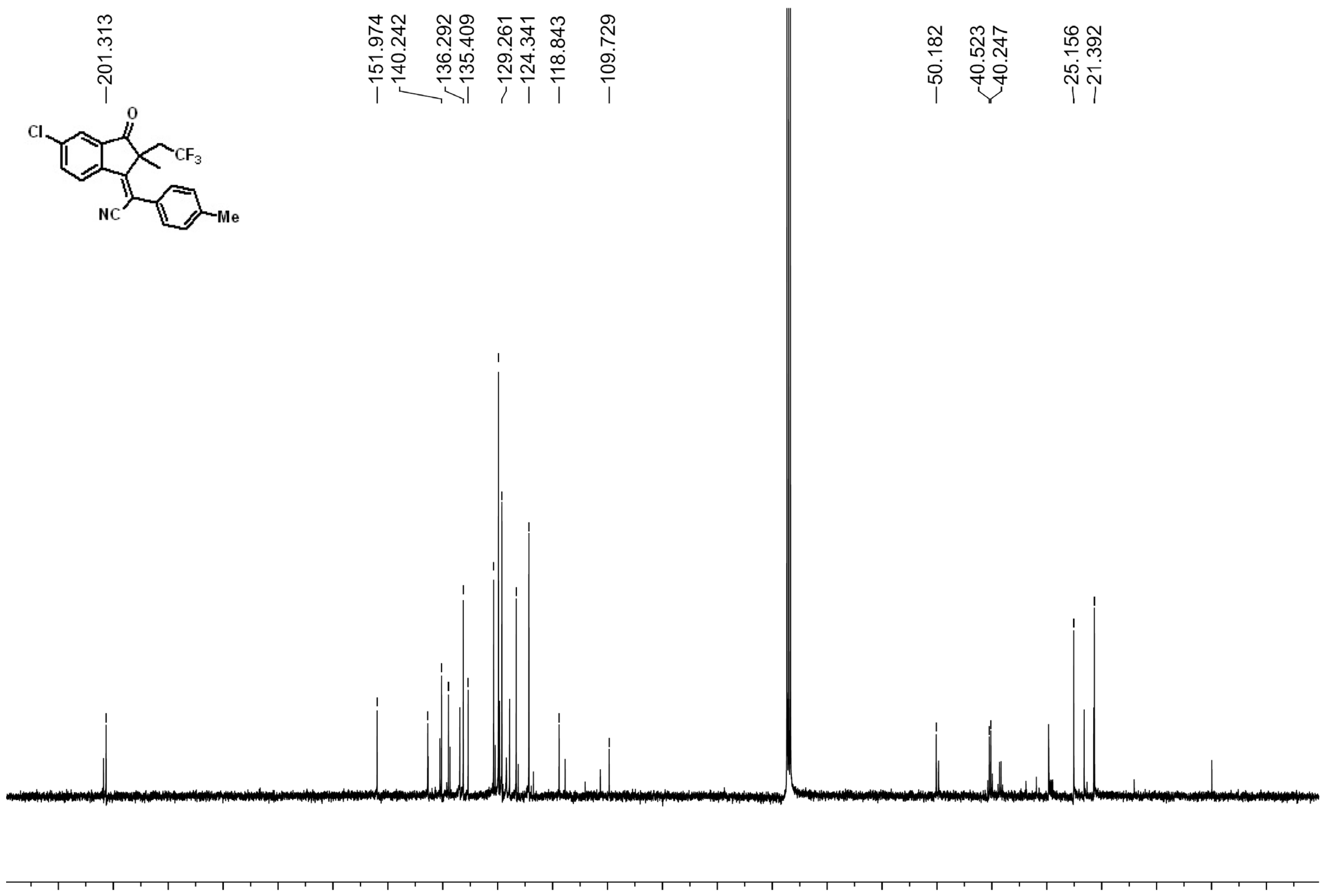

^{13}C NMR Spectrum of Compound **3m**

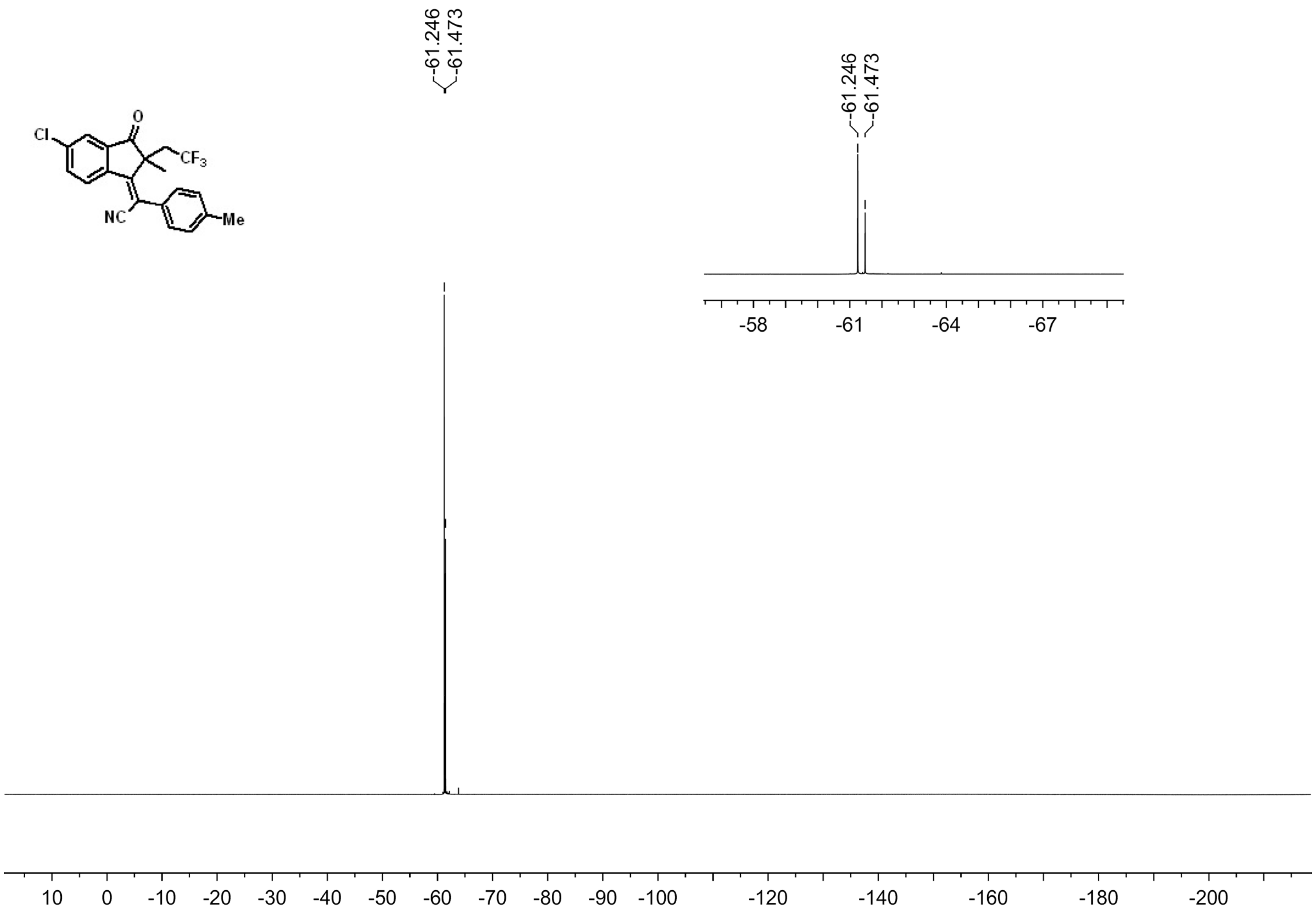

^{19}F NMR Spectrum of Compound **3m**

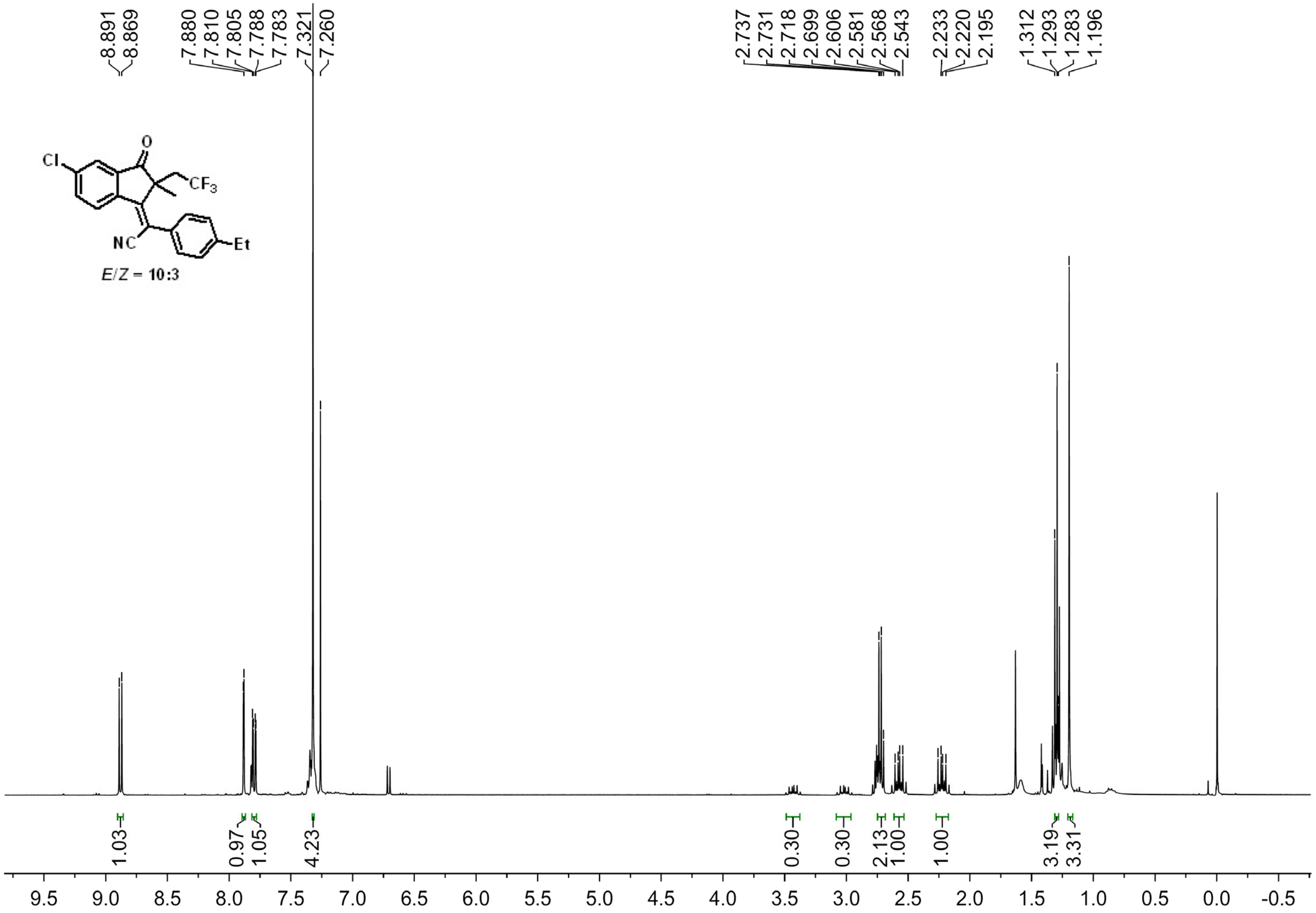

^{1}H NMR Spectrum of Compound **3n**

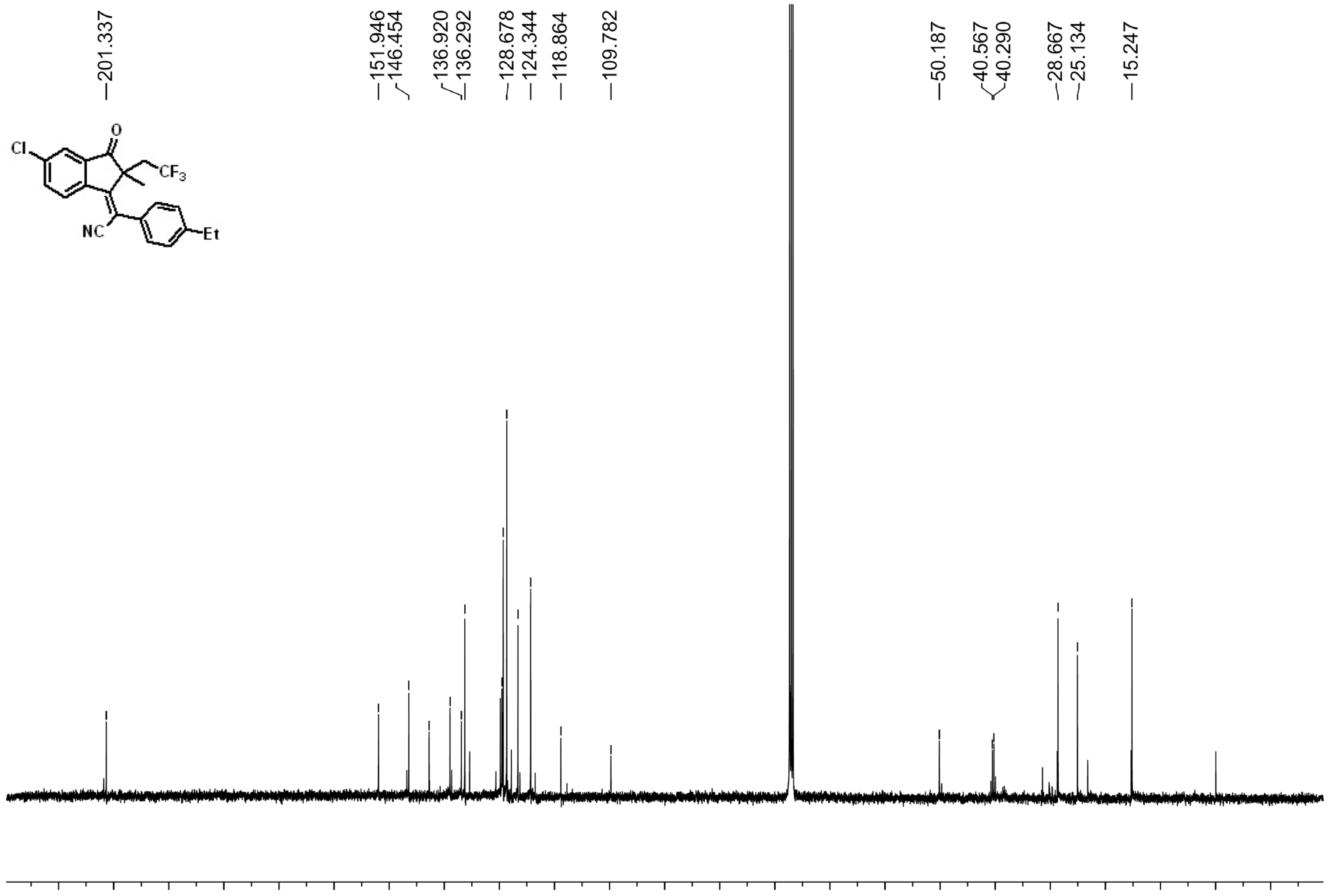

^{13}C NMR Spectrum of Compound **3n**

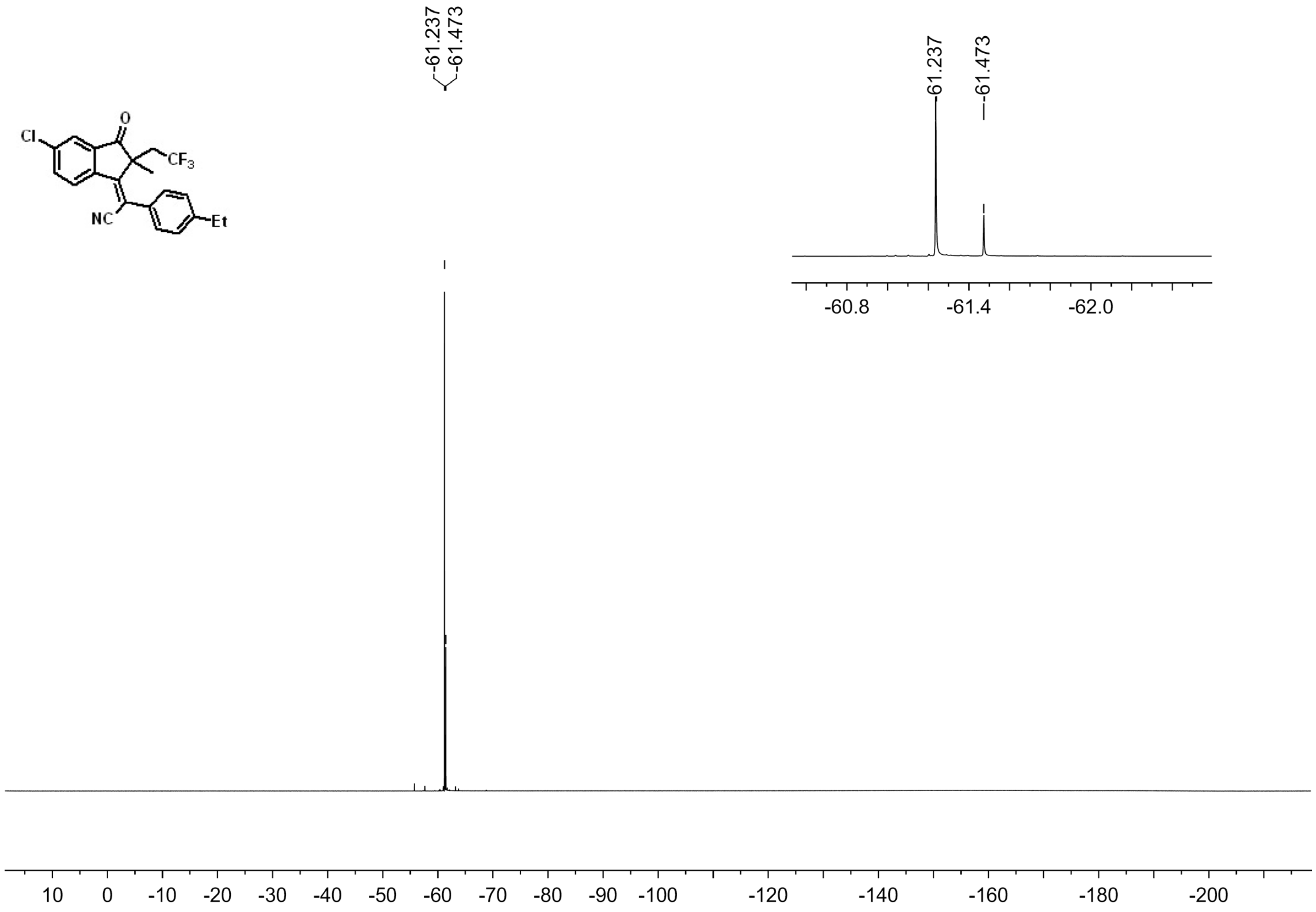

^{19}F NMR Spectrum of Compound **3n**

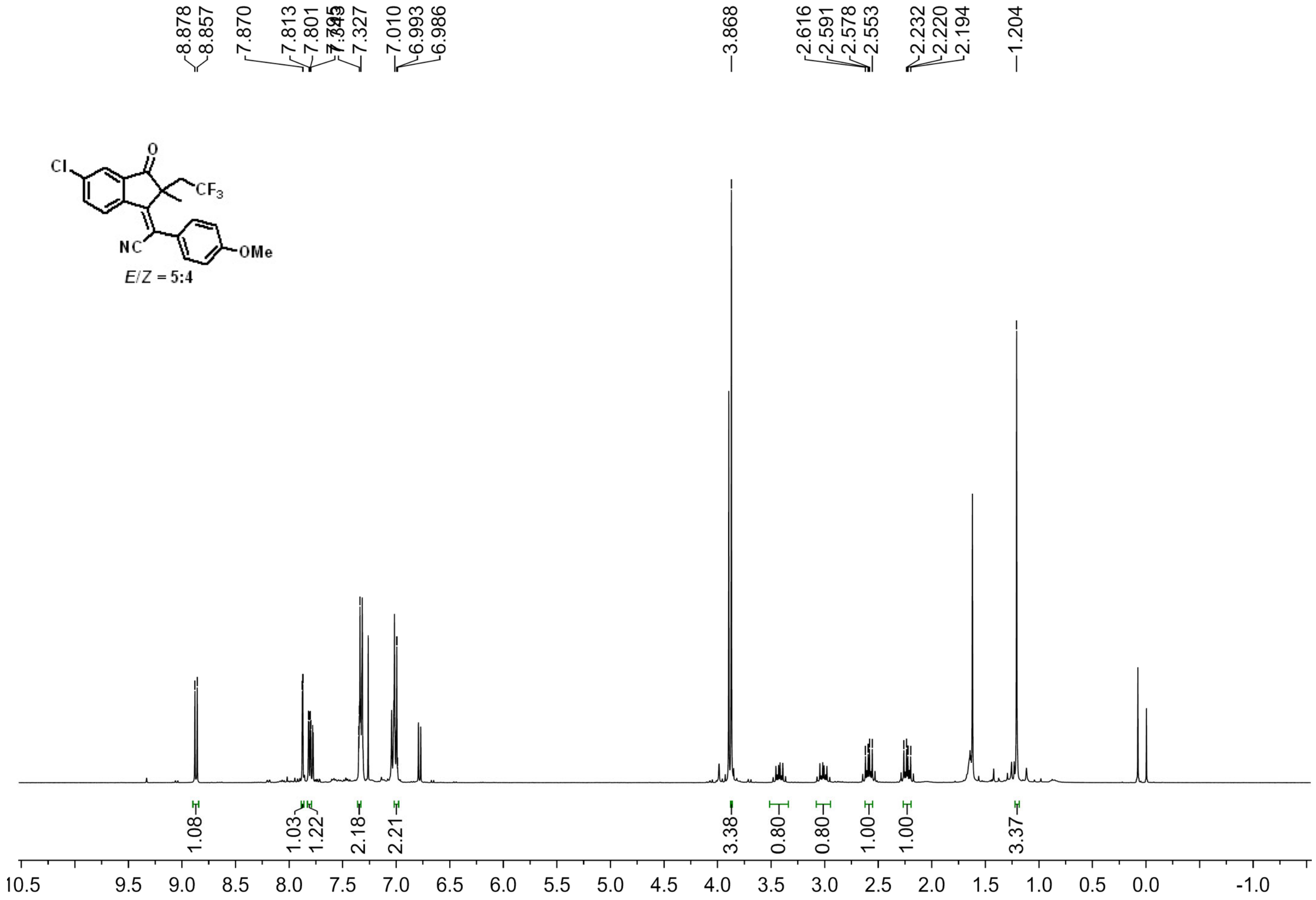

^{1}H NMR Spectrum of Compound **3o**

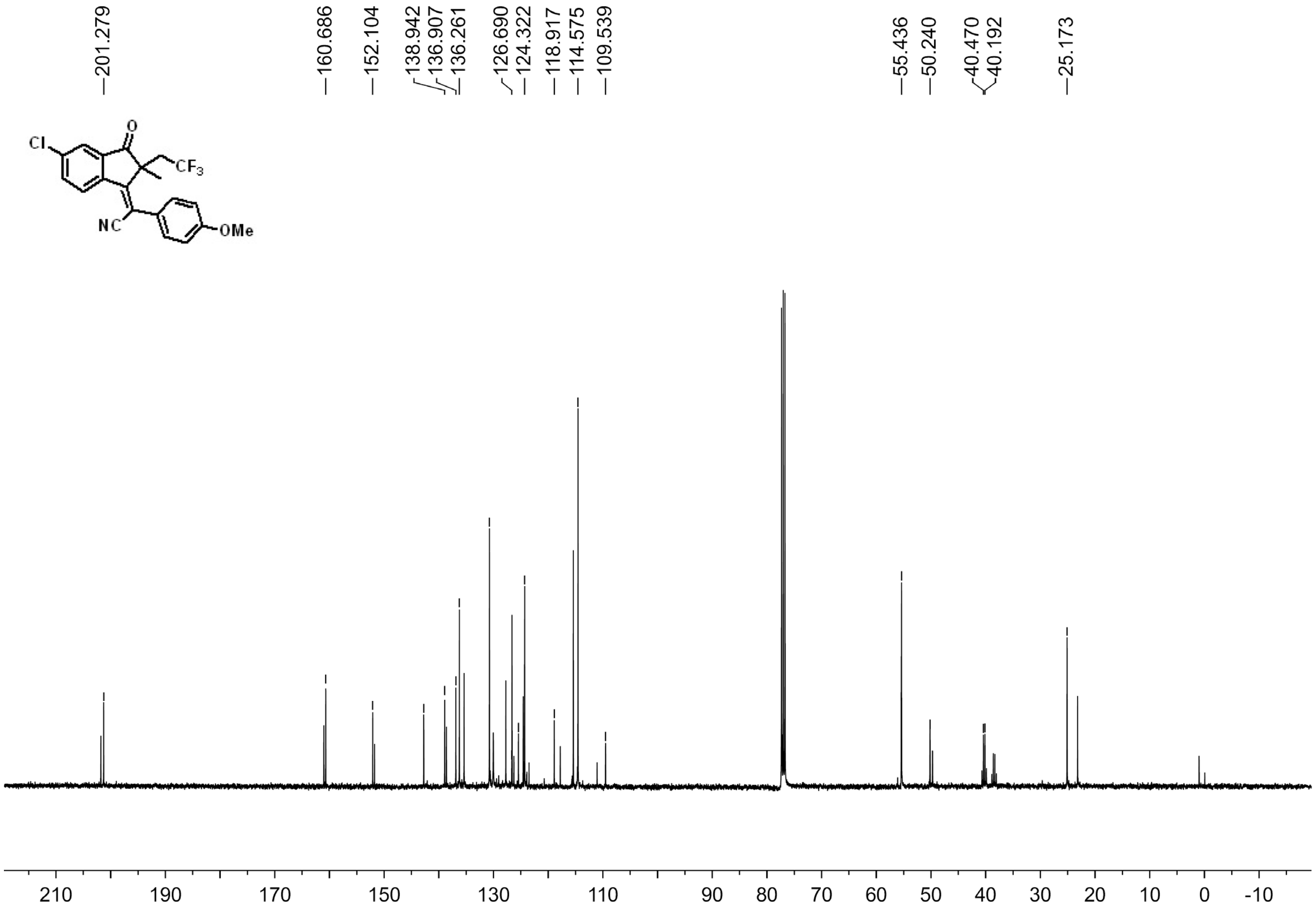

^{13}C NMR Spectrum of Compound **3o**

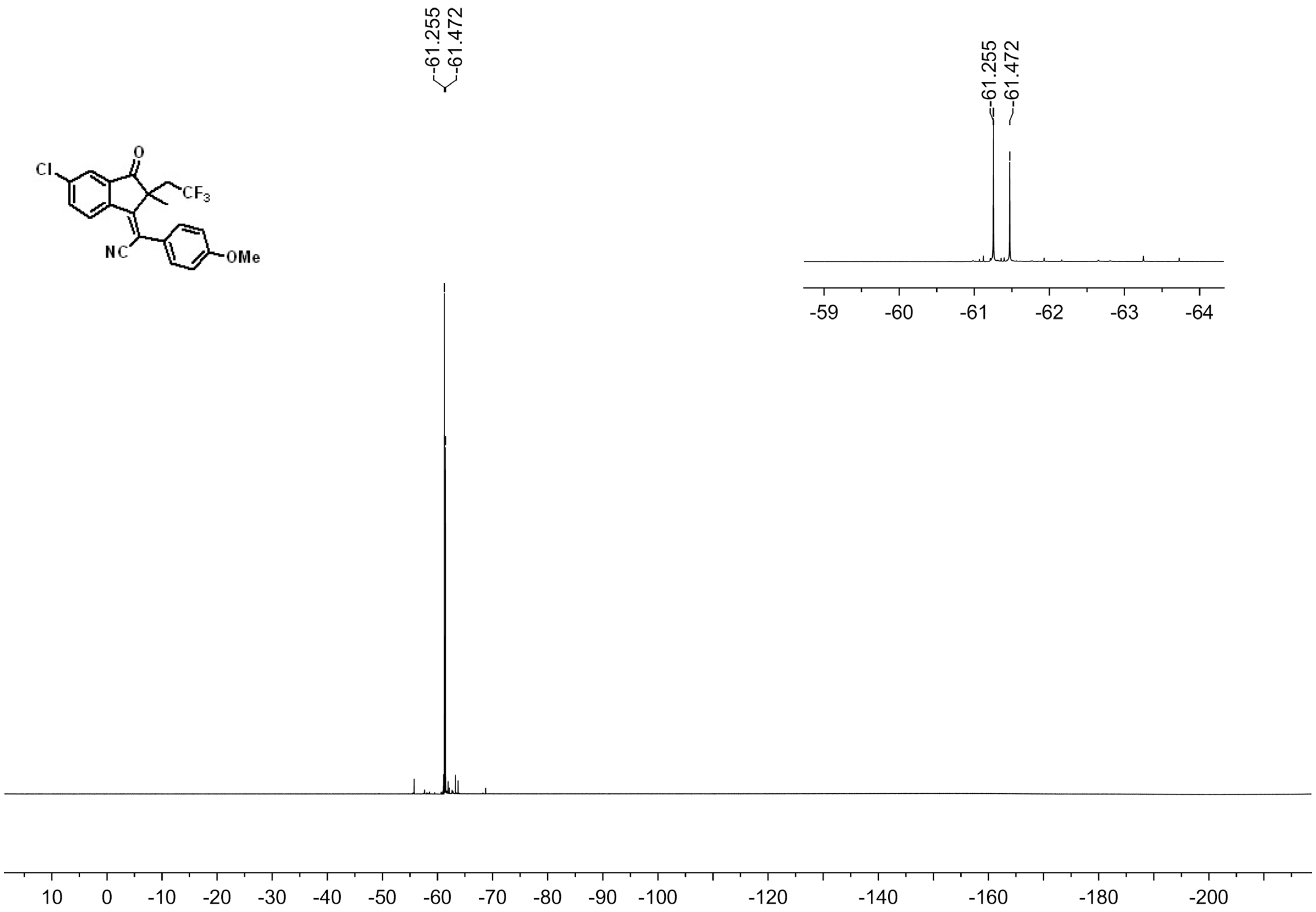

^{19}F NMR Spectrum of Compound **3o**

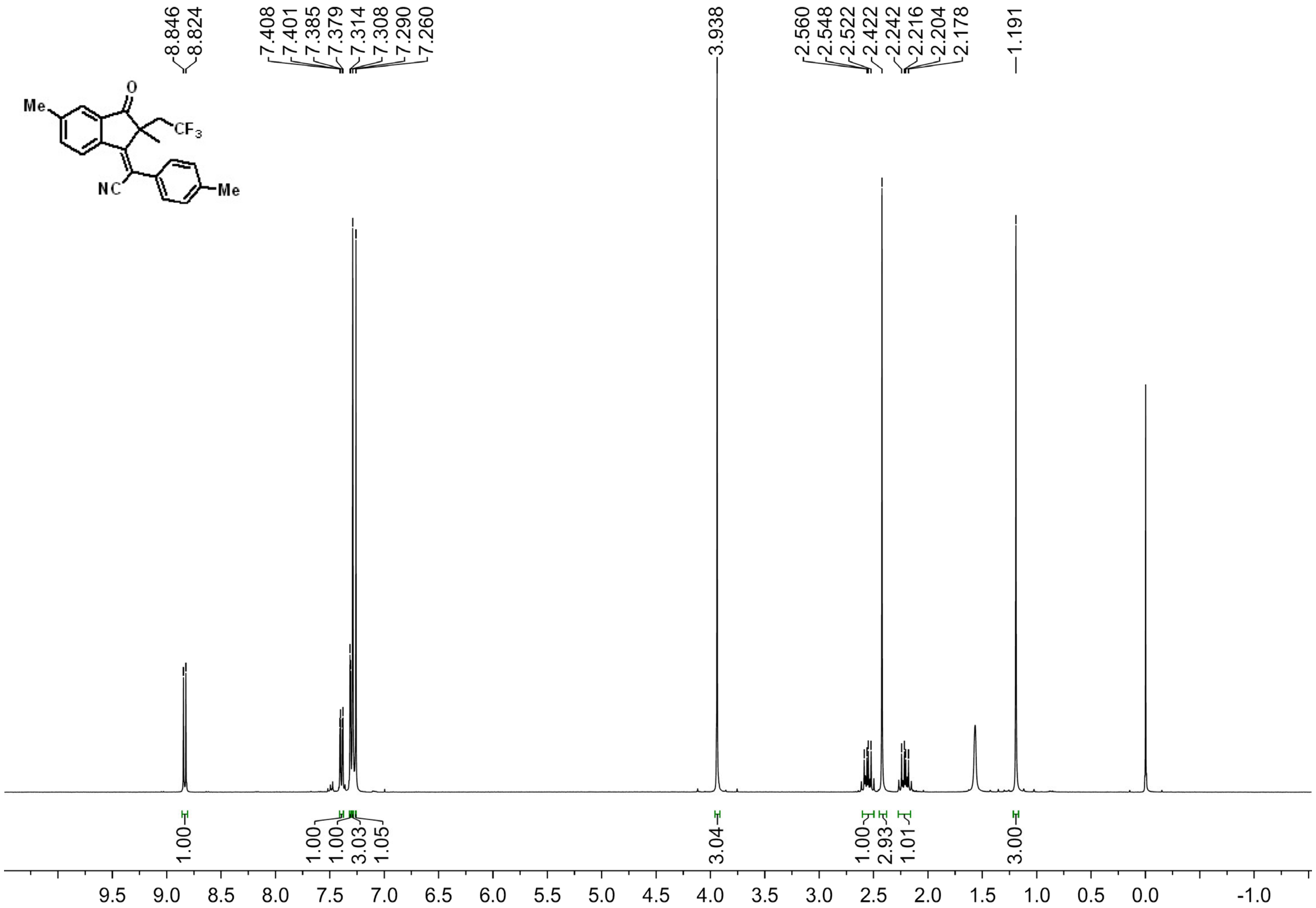

^{1}H NMR Spectrum of Compound **3p**

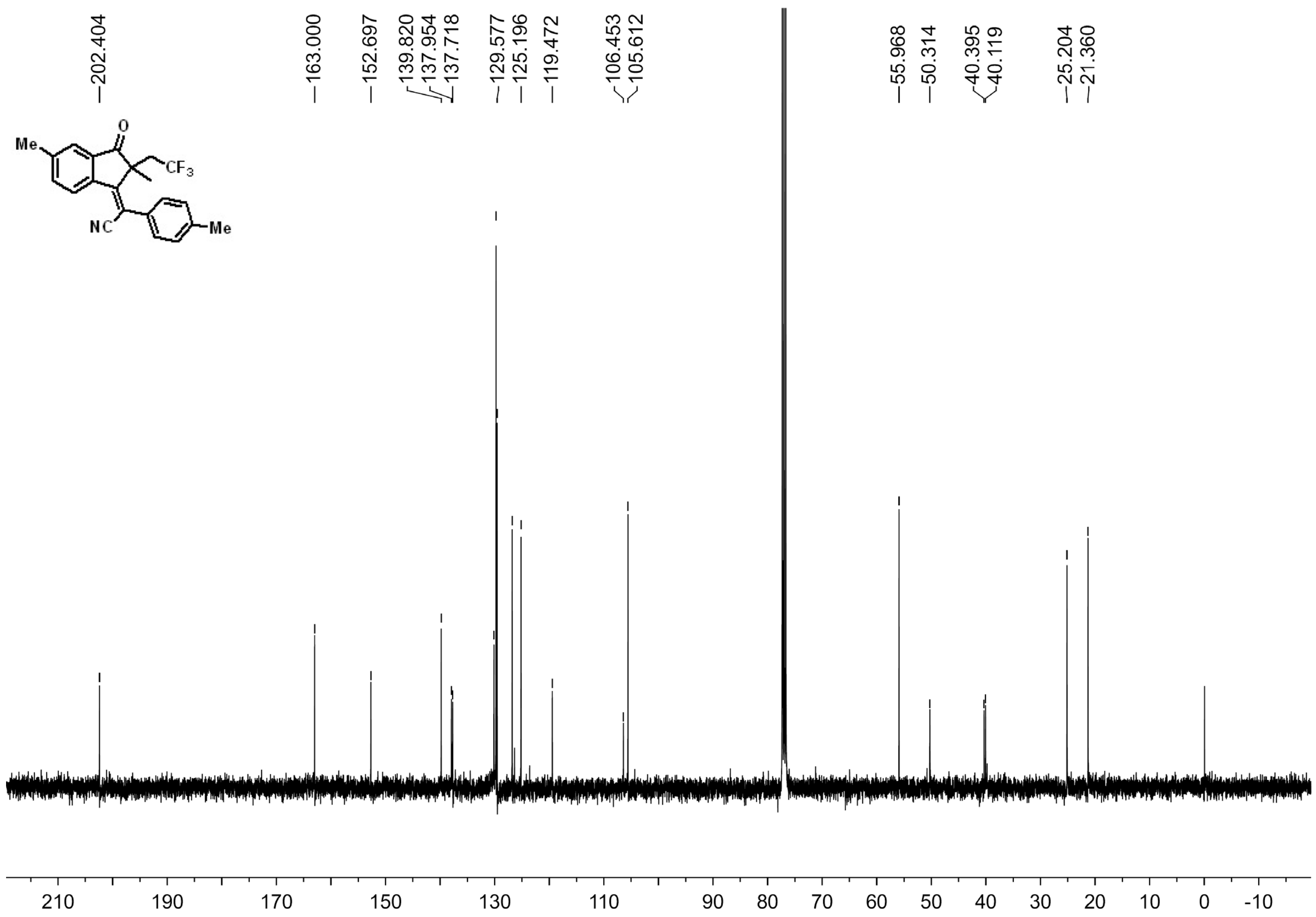

^{13}C NMR Spectrum of Compound **3p**

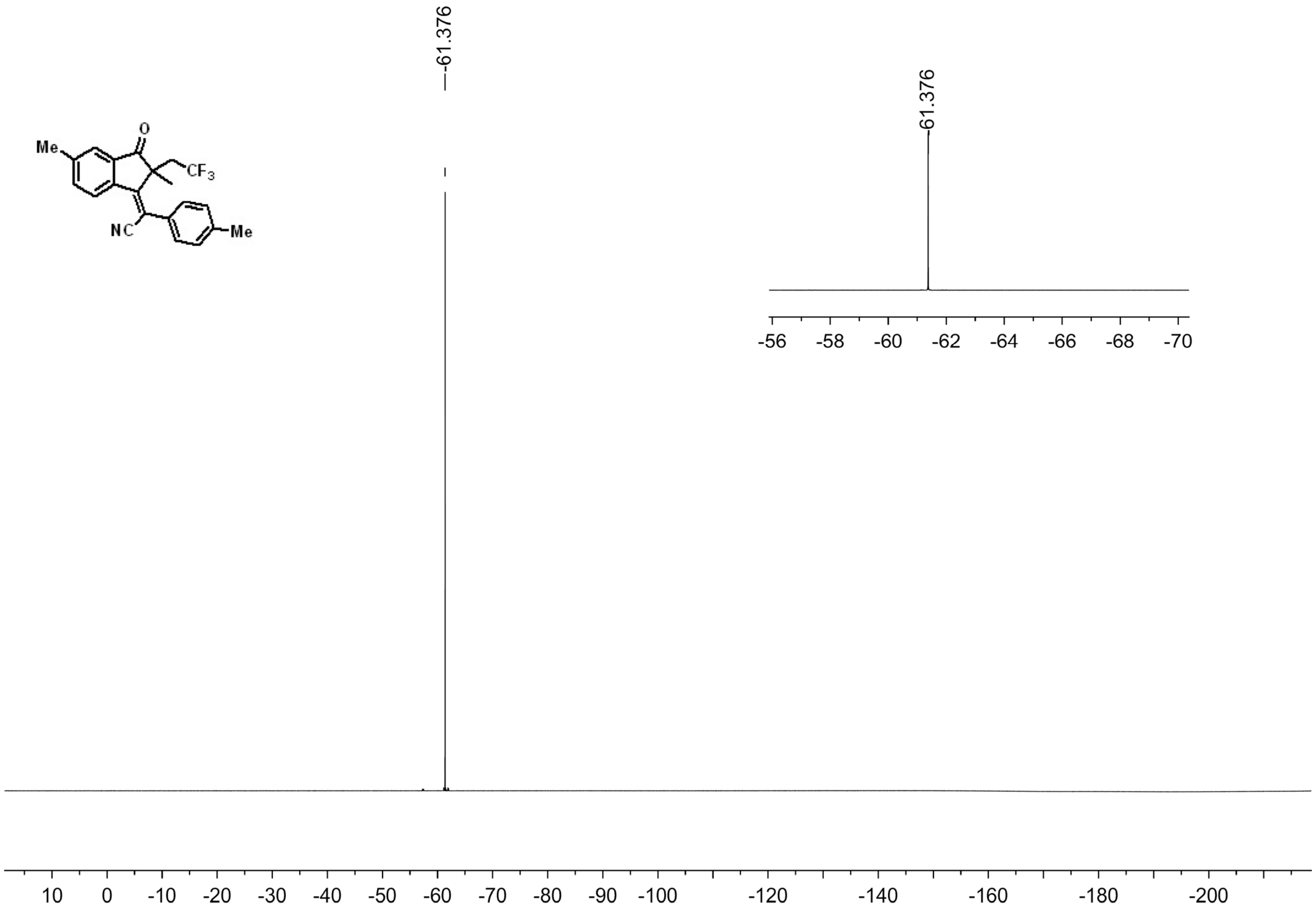

^{19}F NMR Spectrum of Compound **3p**

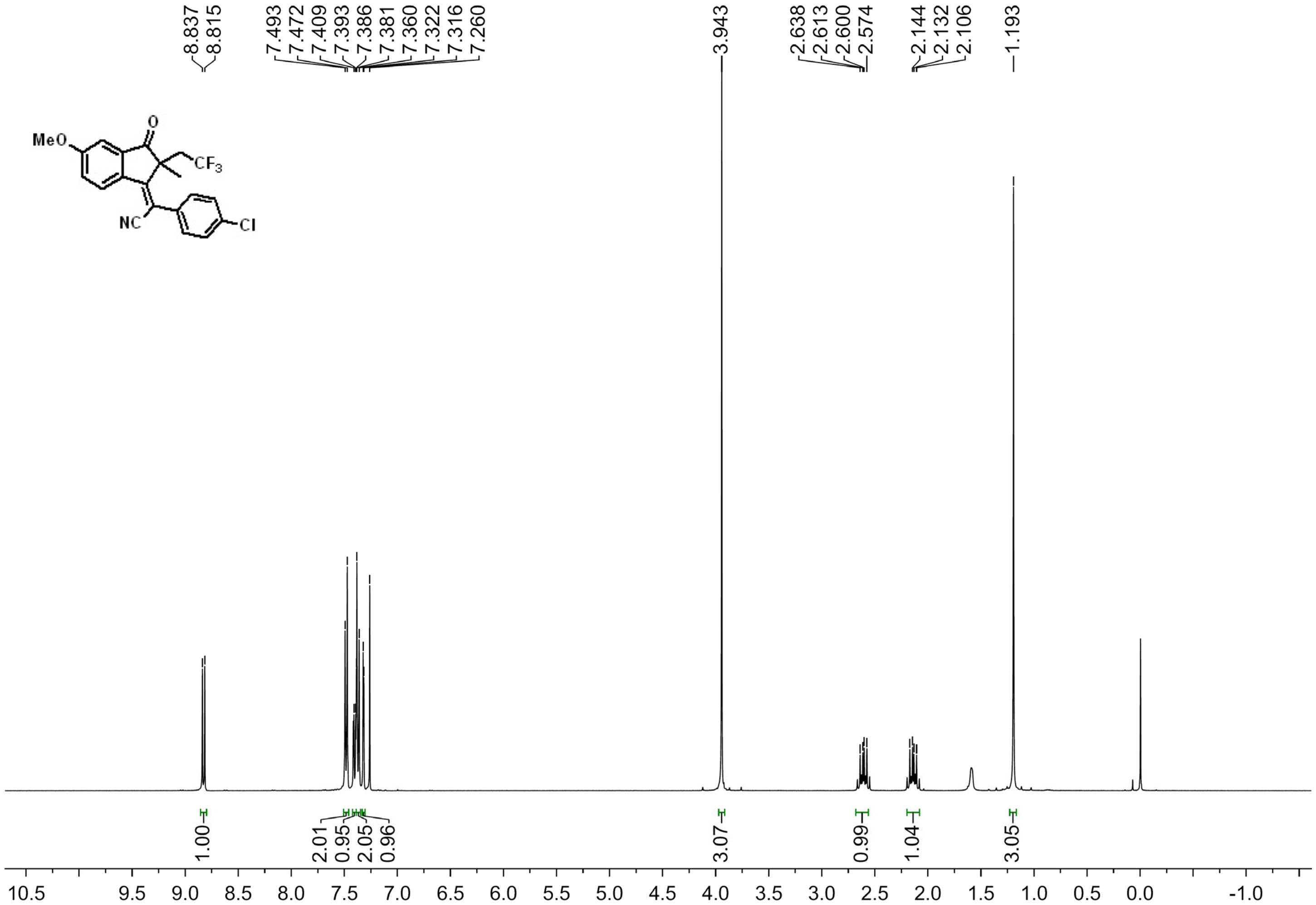

^{1}H NMR Spectrum of Compound **3q**

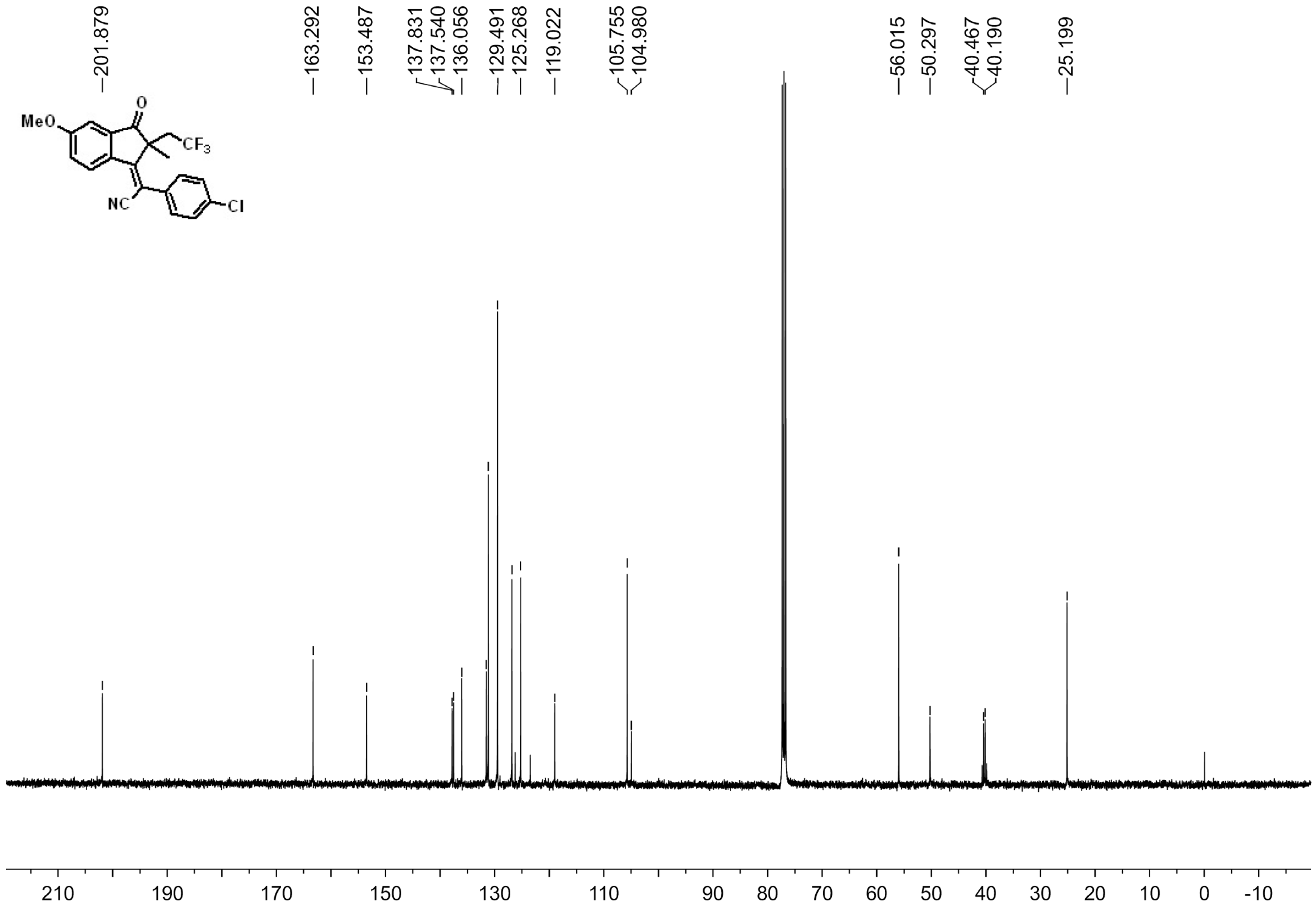

^{13}C NMR Spectrum of Compound **3q**

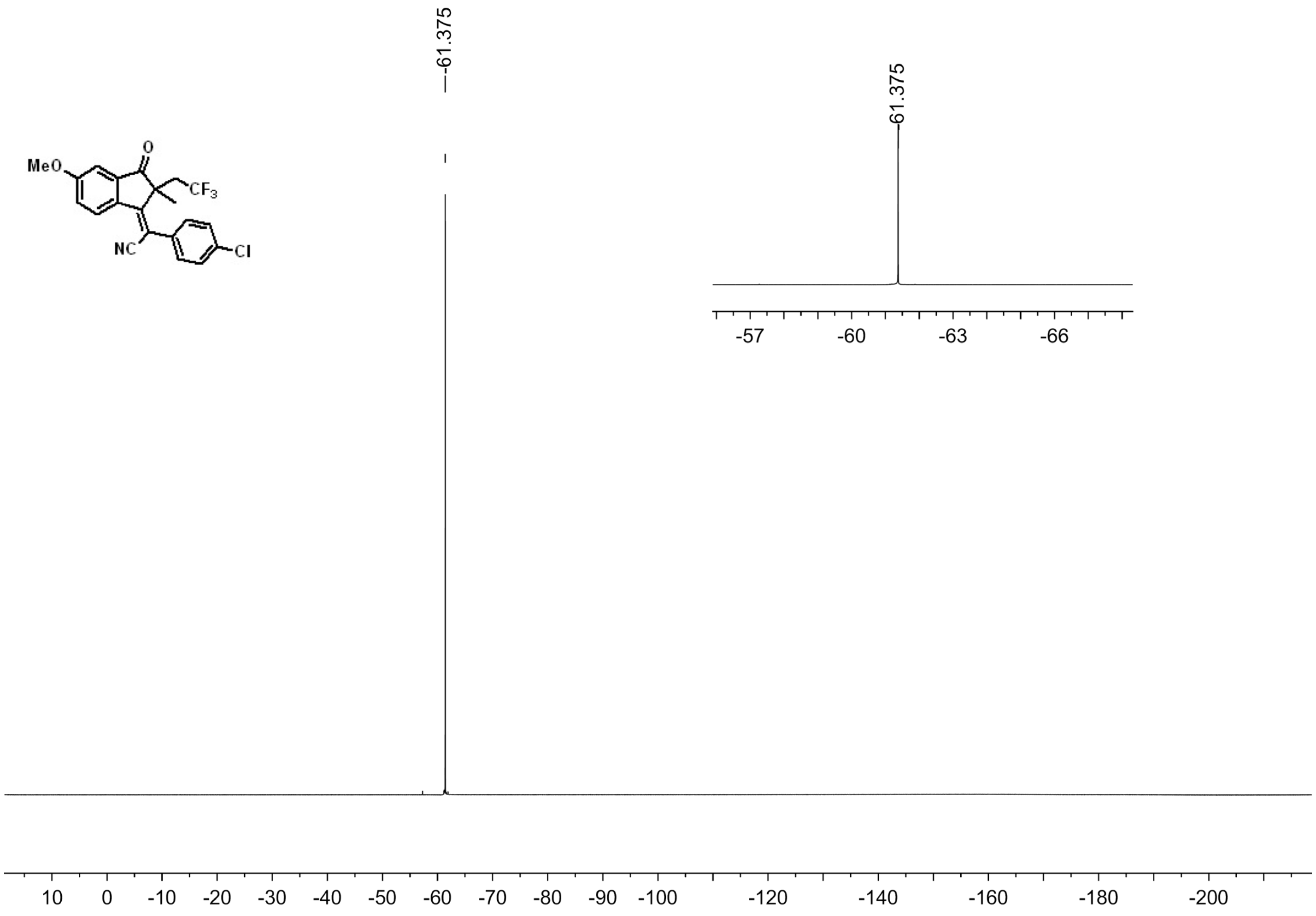

^{19}F NMR Spectrum of Compound **3q**

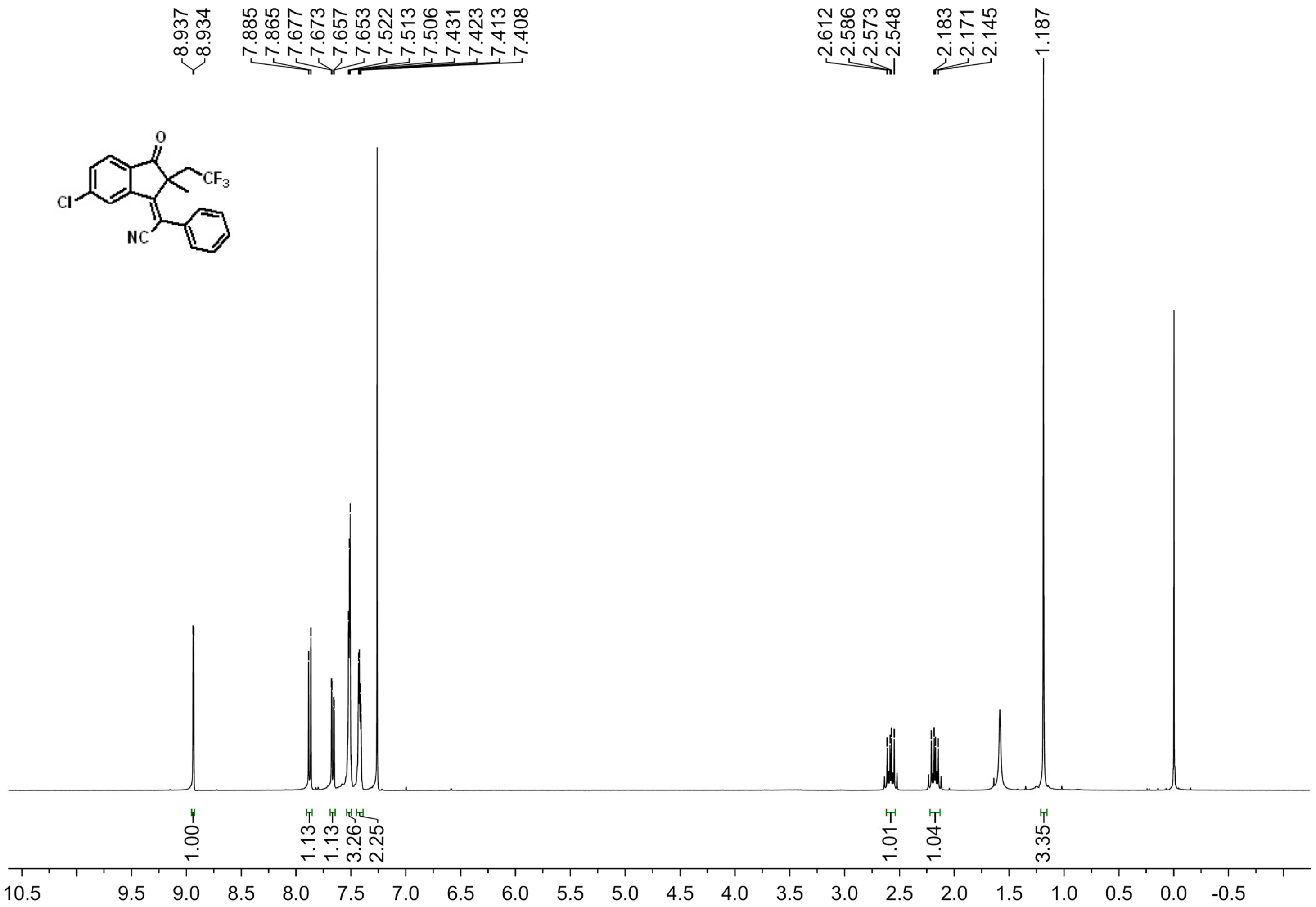

^{1}H NMR Spectrum of Compound **3r**

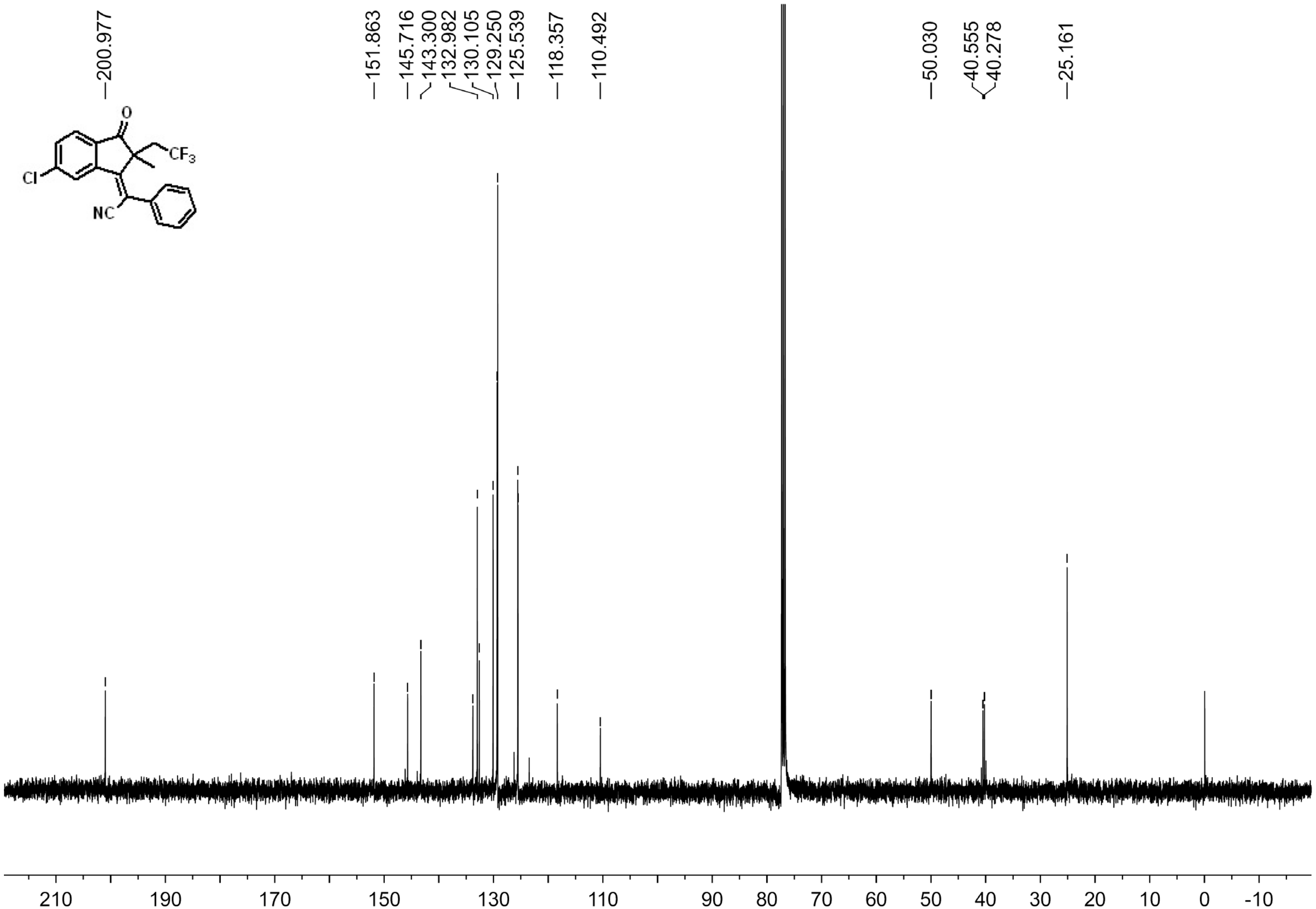

^{13}C NMR Spectrum of Compound **3r**

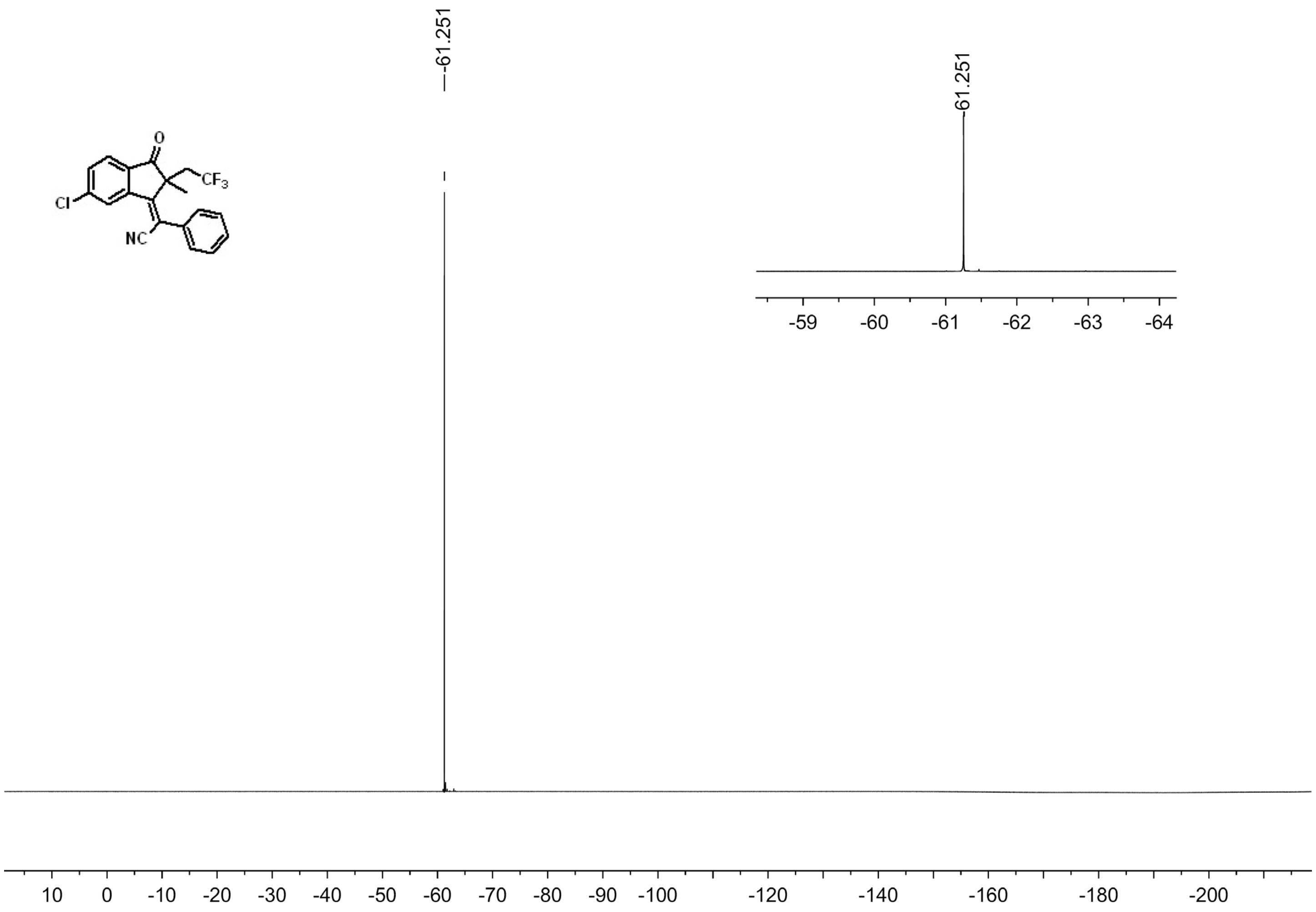

^{19}F NMR Spectrum of Compound **3r**

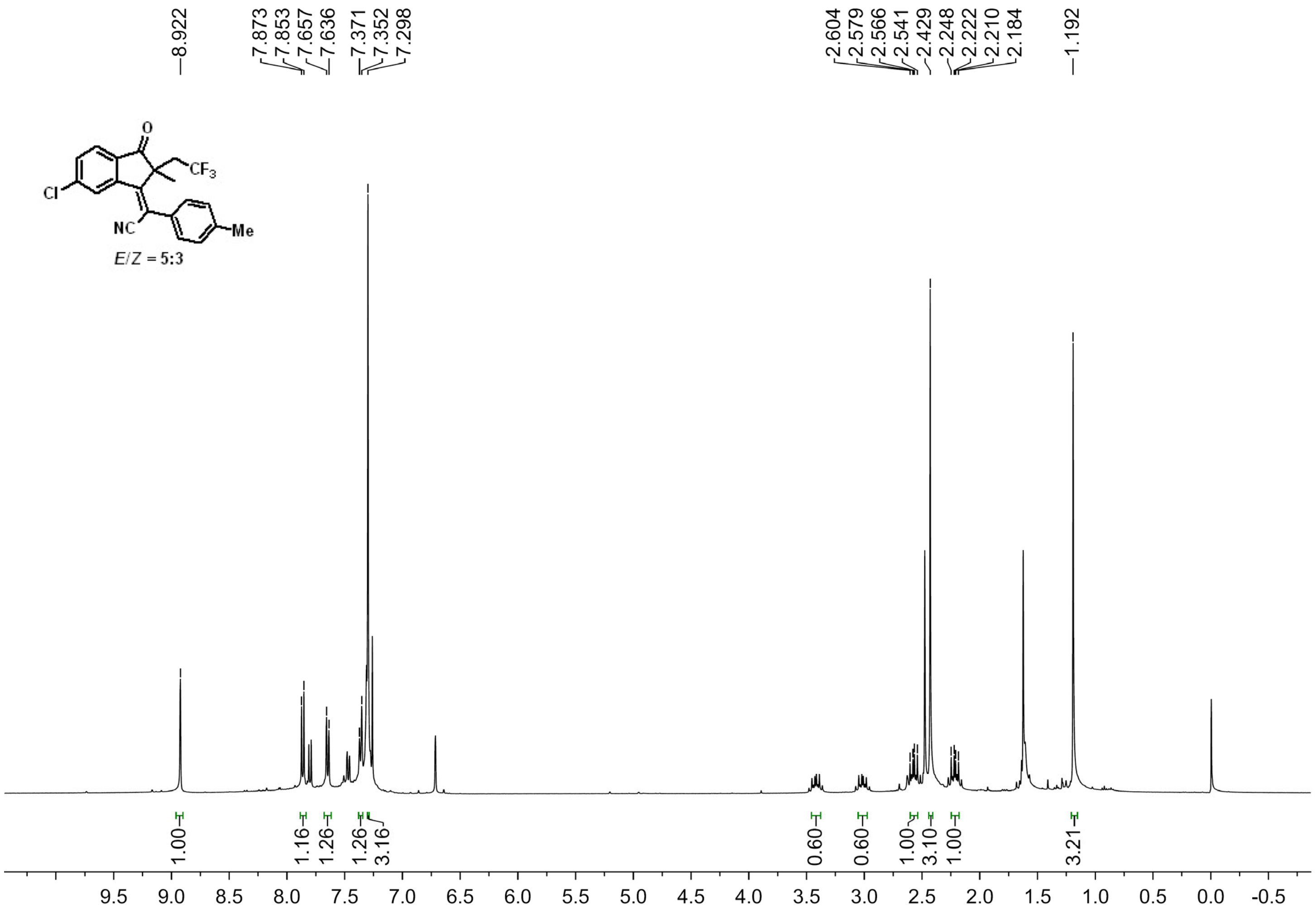

^{1}H NMR Spectrum of Compound **3s**

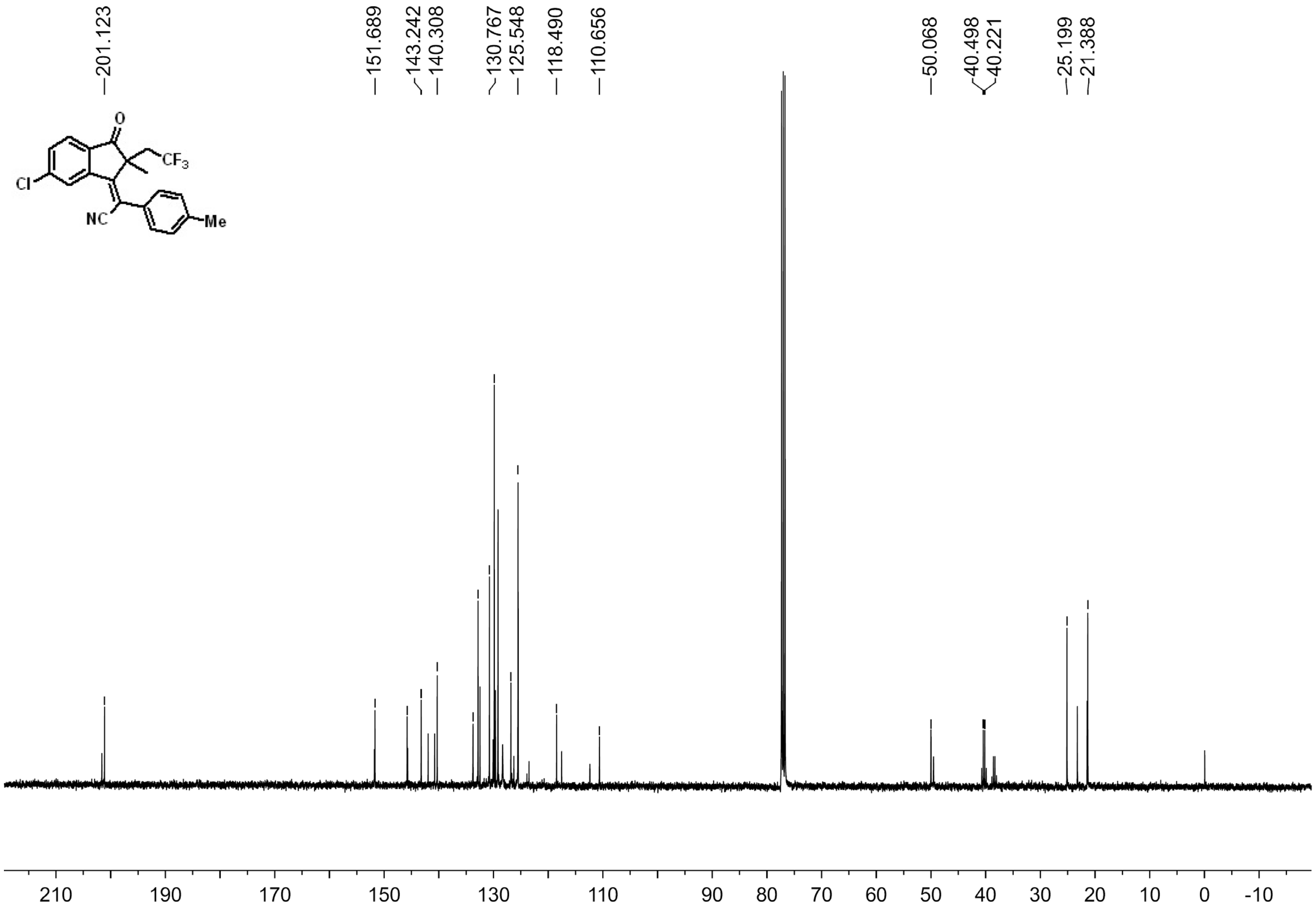

^{13}C NMR Spectrum of Compound **3s**

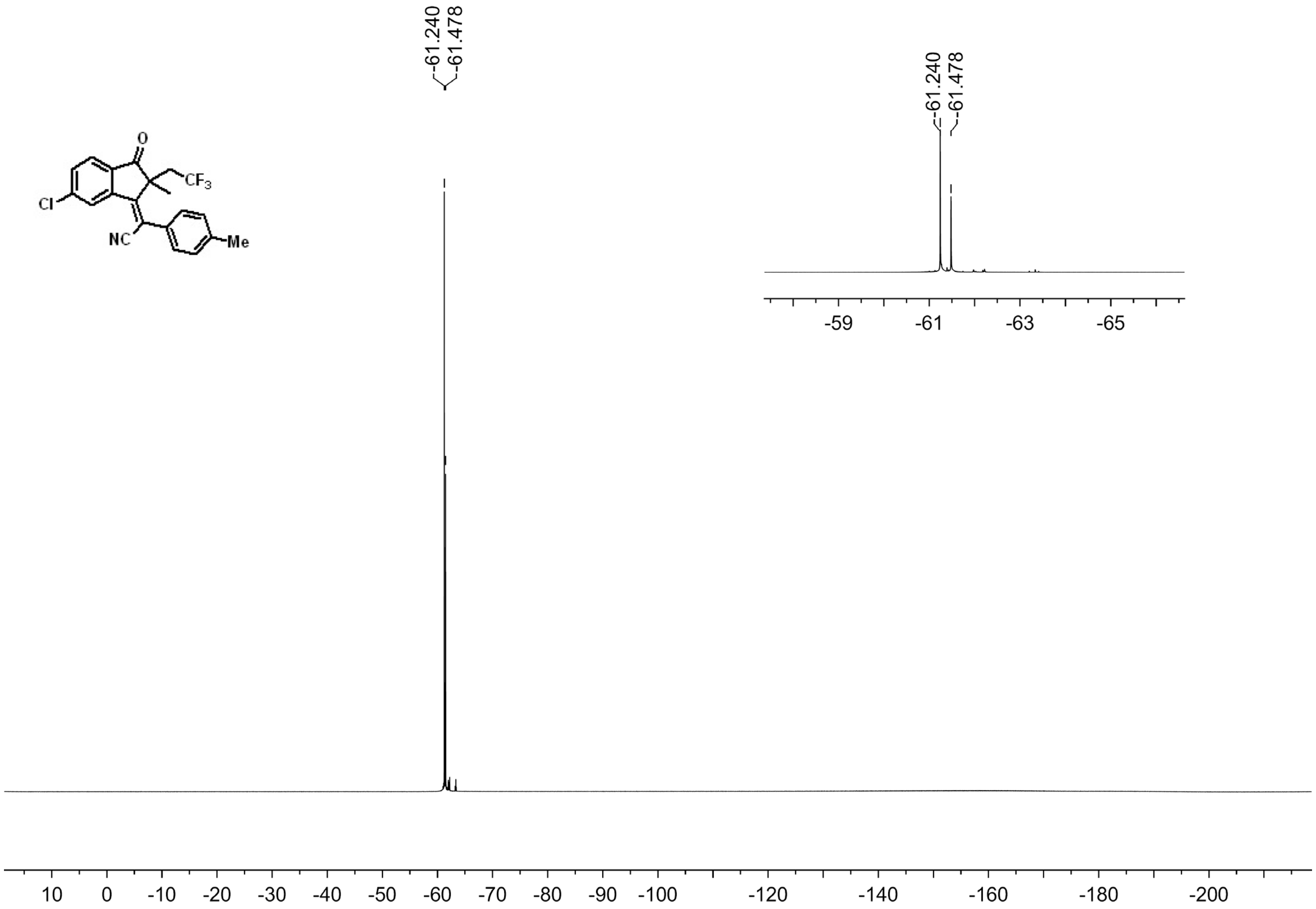

^{19}F NMR Spectrum of Compound **3s**

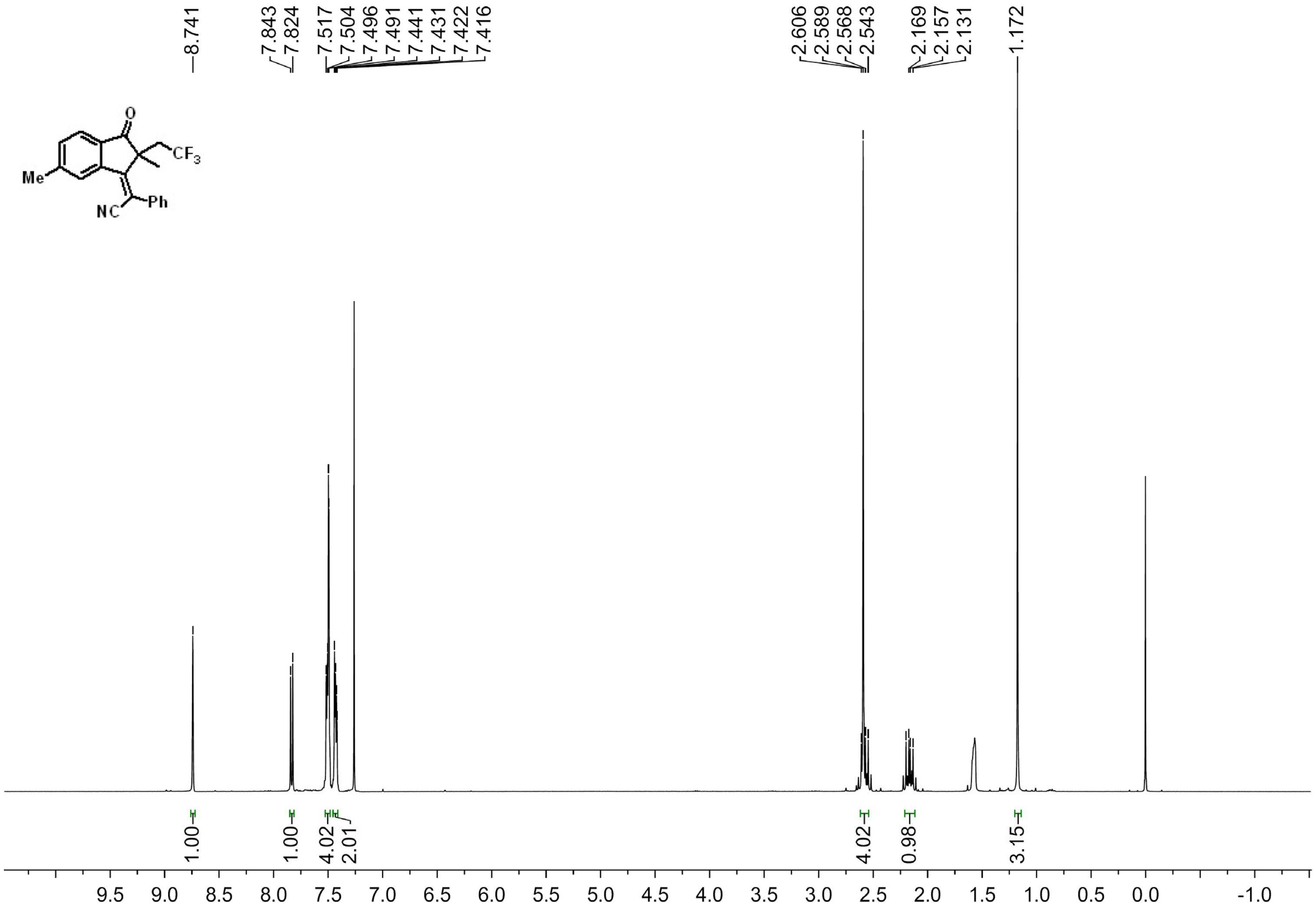

^{1}H NMR Spectrum of Compound **3t**

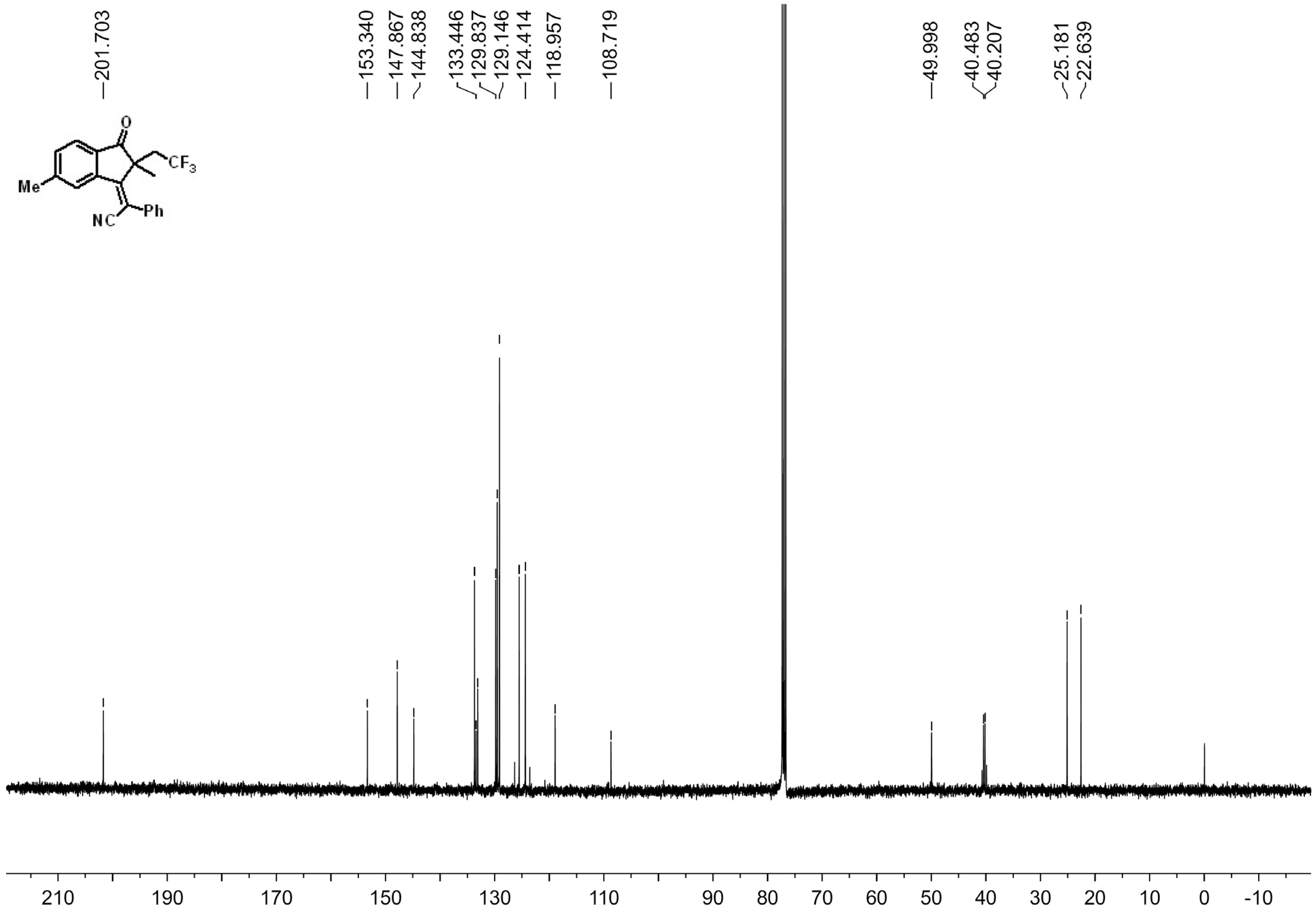

^{13}C NMR Spectrum of Compound **3t**

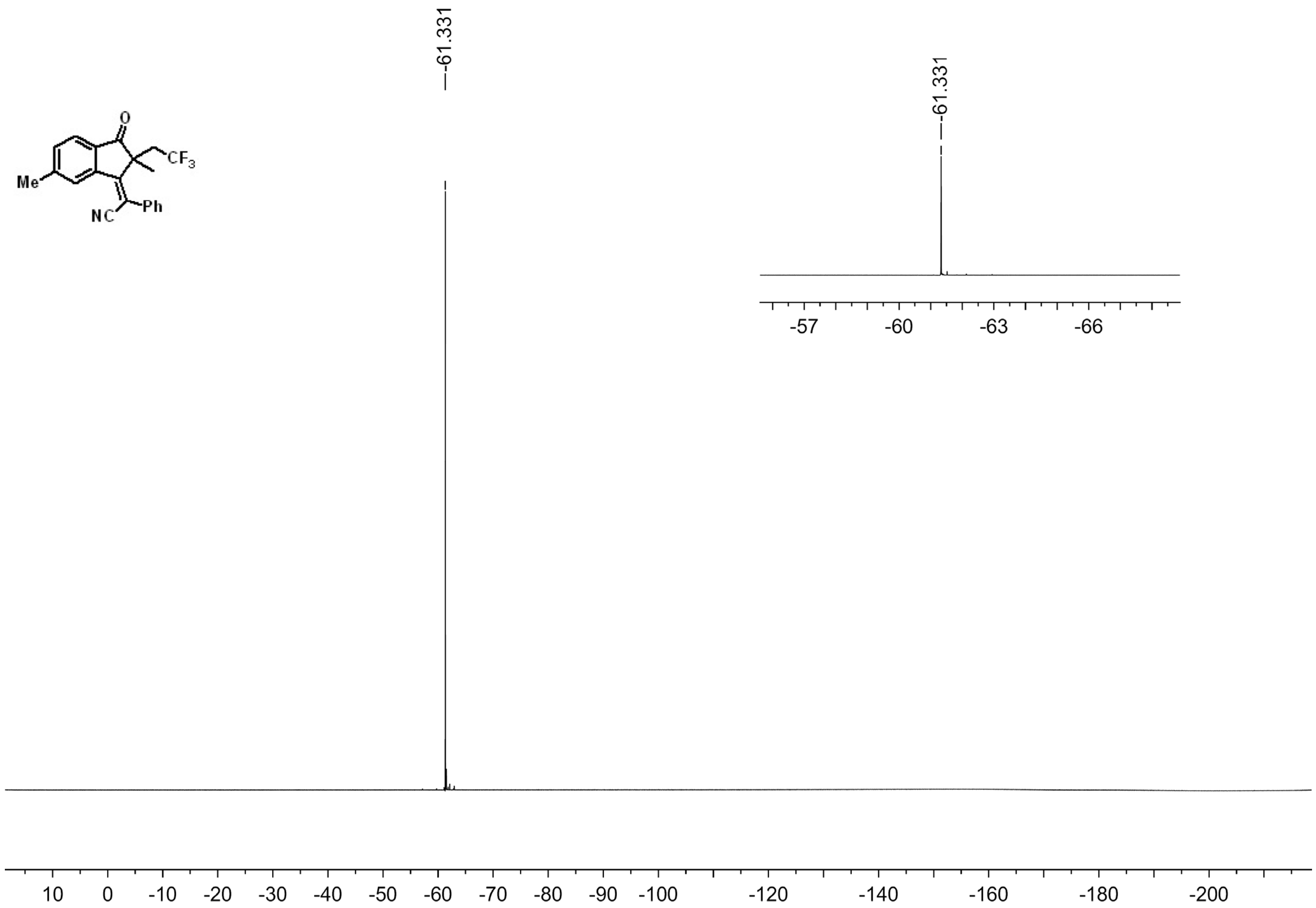

^{19}F NMR Spectrum of Compound **3t**

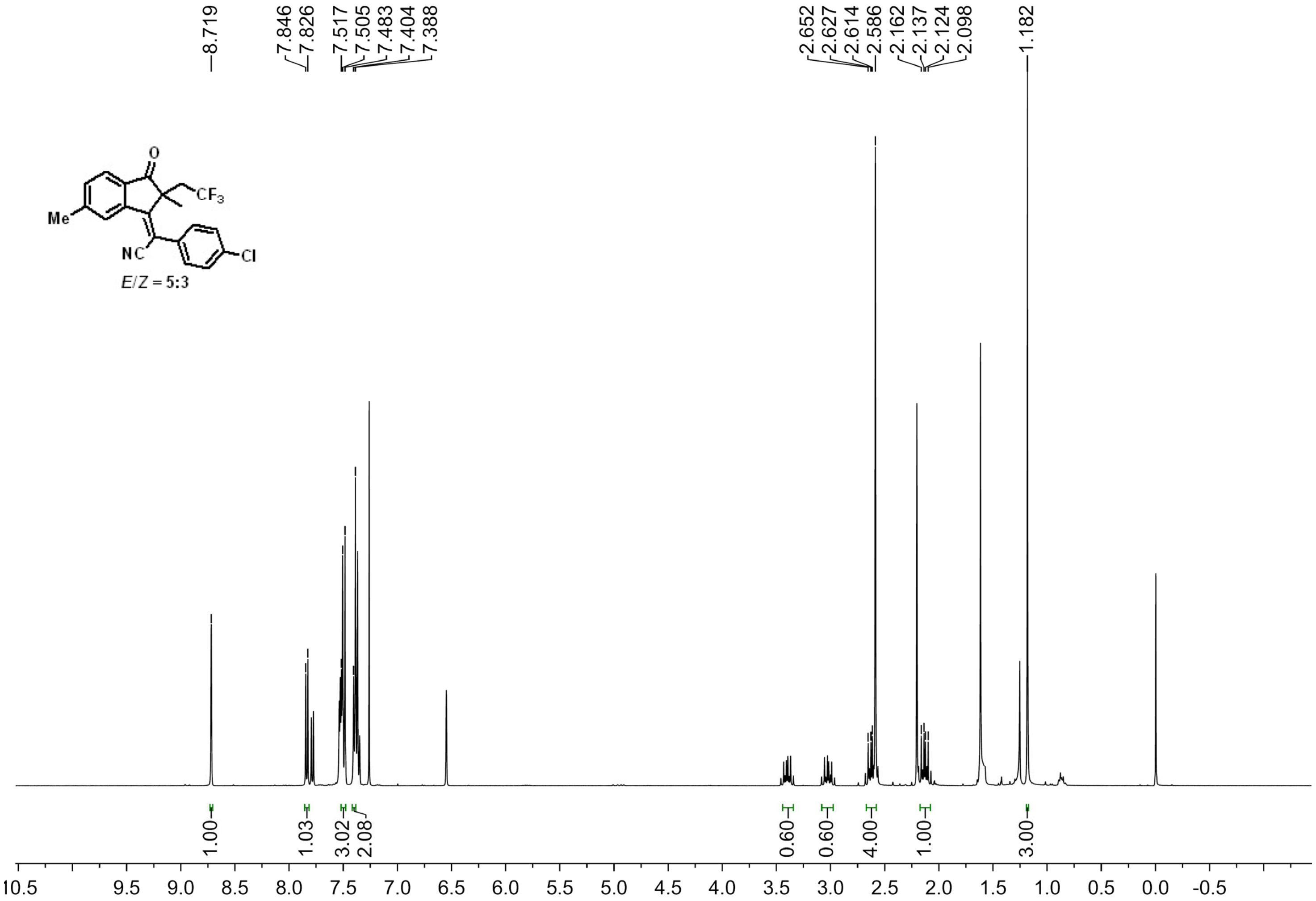

^{1}H NMR Spectrum of Compound **3u**

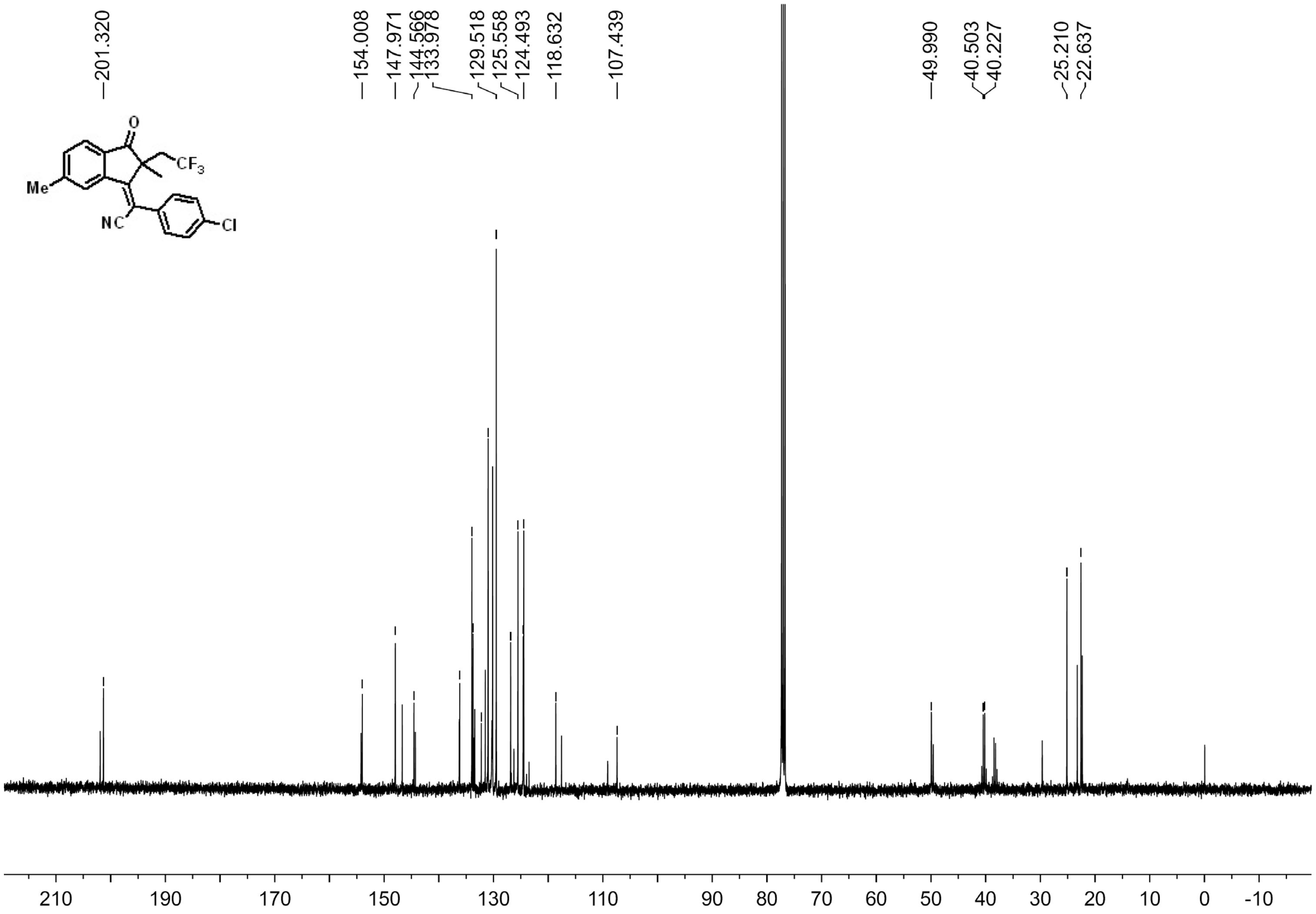

^{13}C NMR Spectrum of Compound **3u**

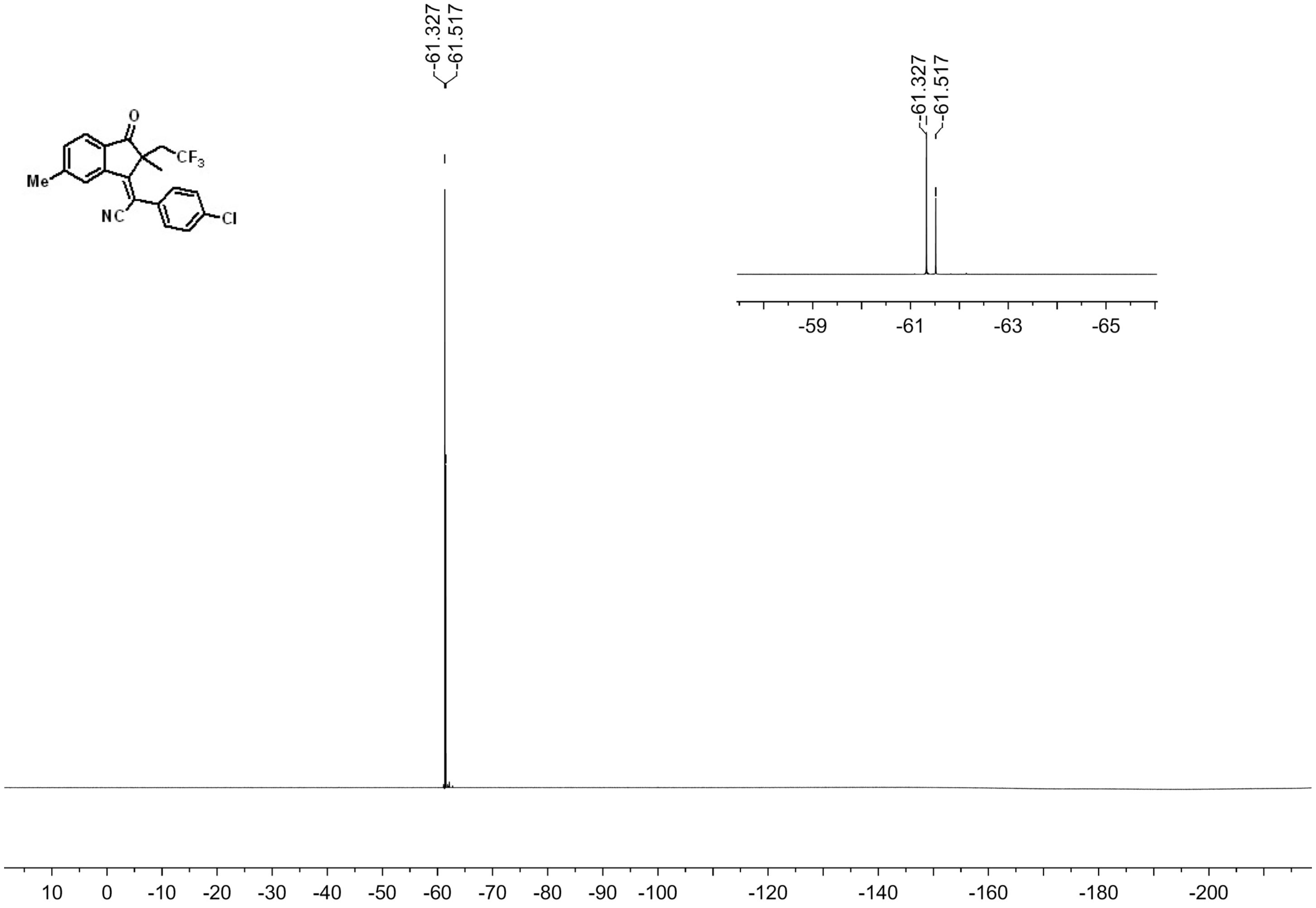

^{19}F NMR Spectrum of Compound **3u**

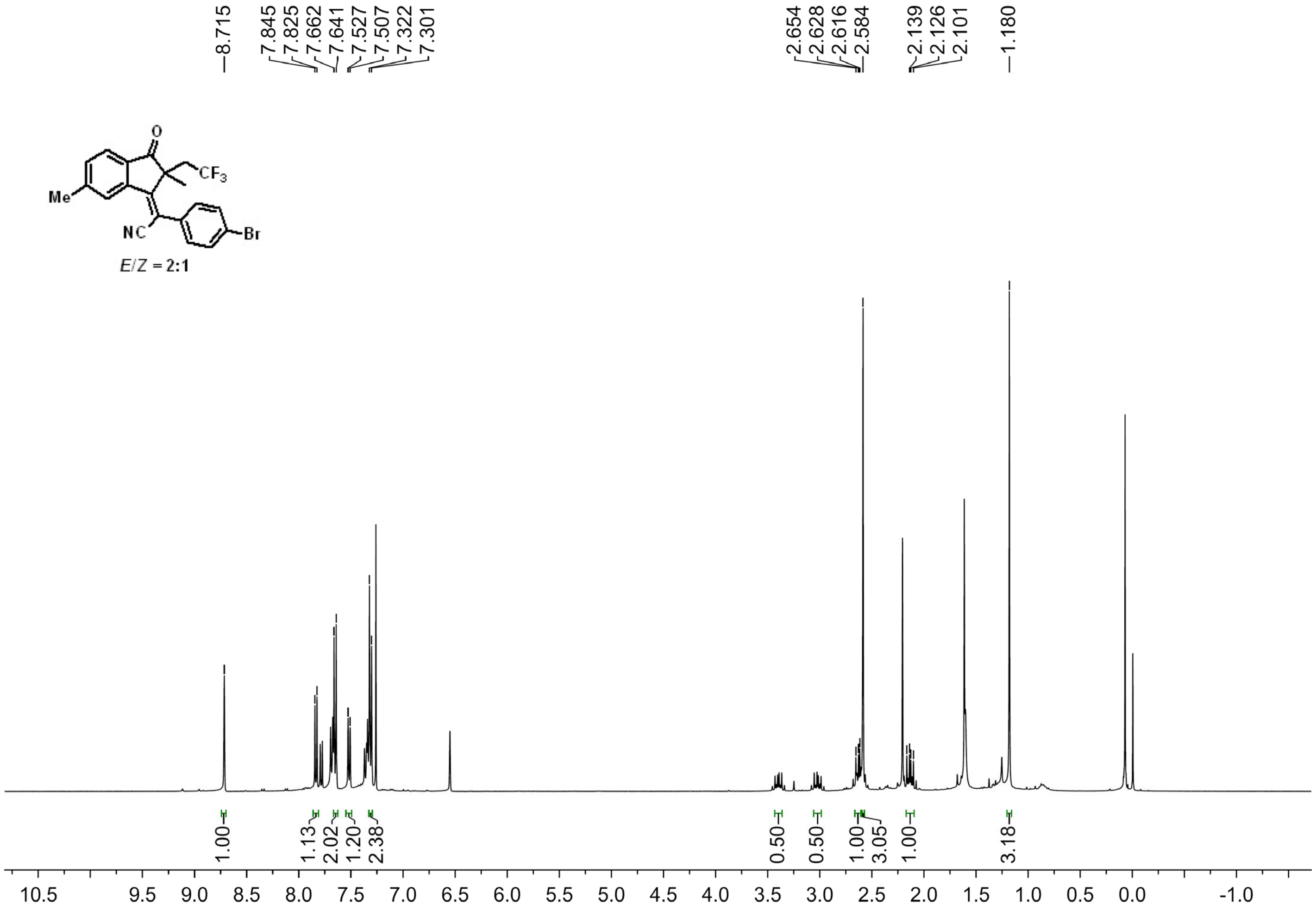

^{1}H NMR Spectrum of Compound **3v**

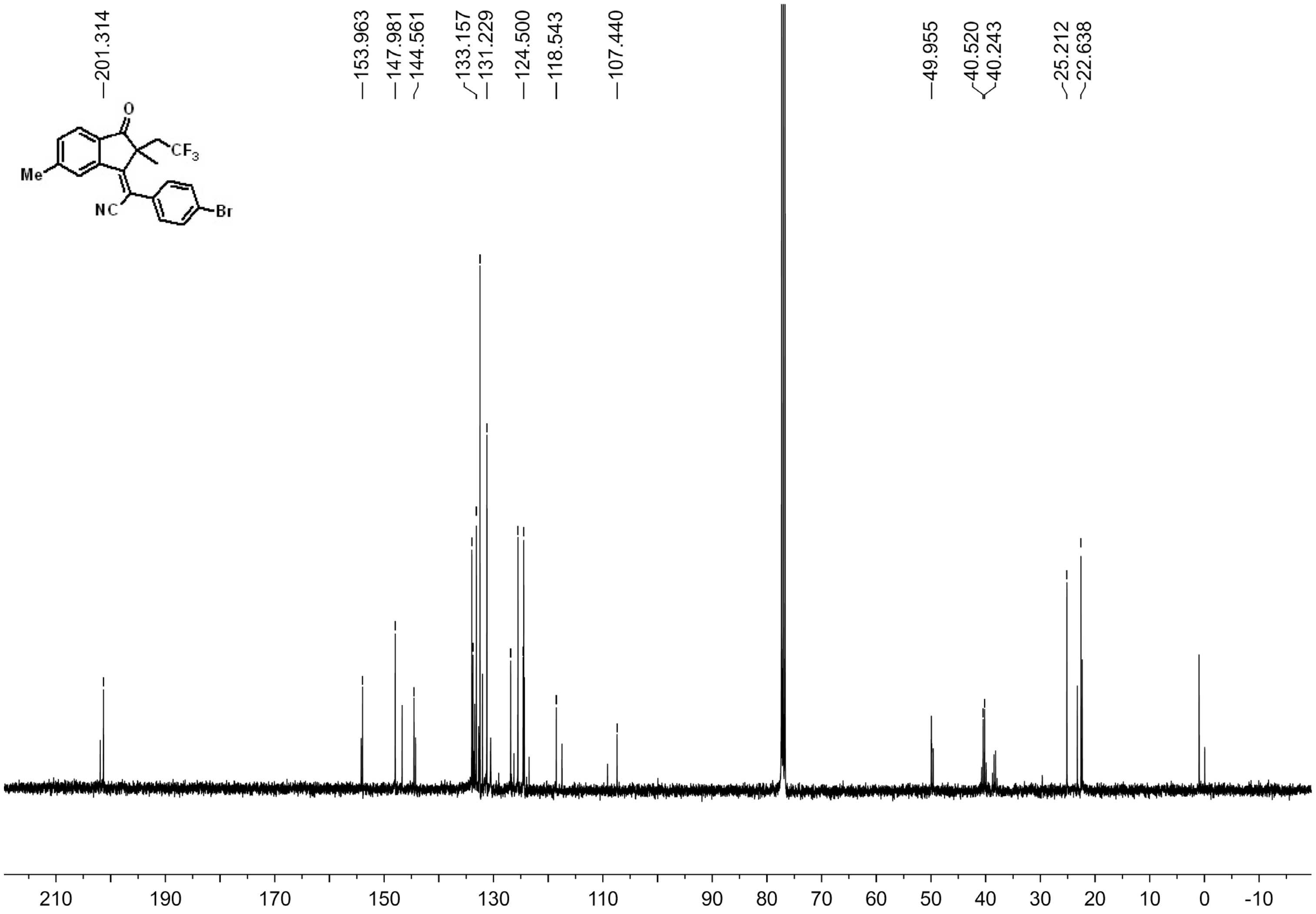

^{13}C NMR Spectrum of Compound **3v**

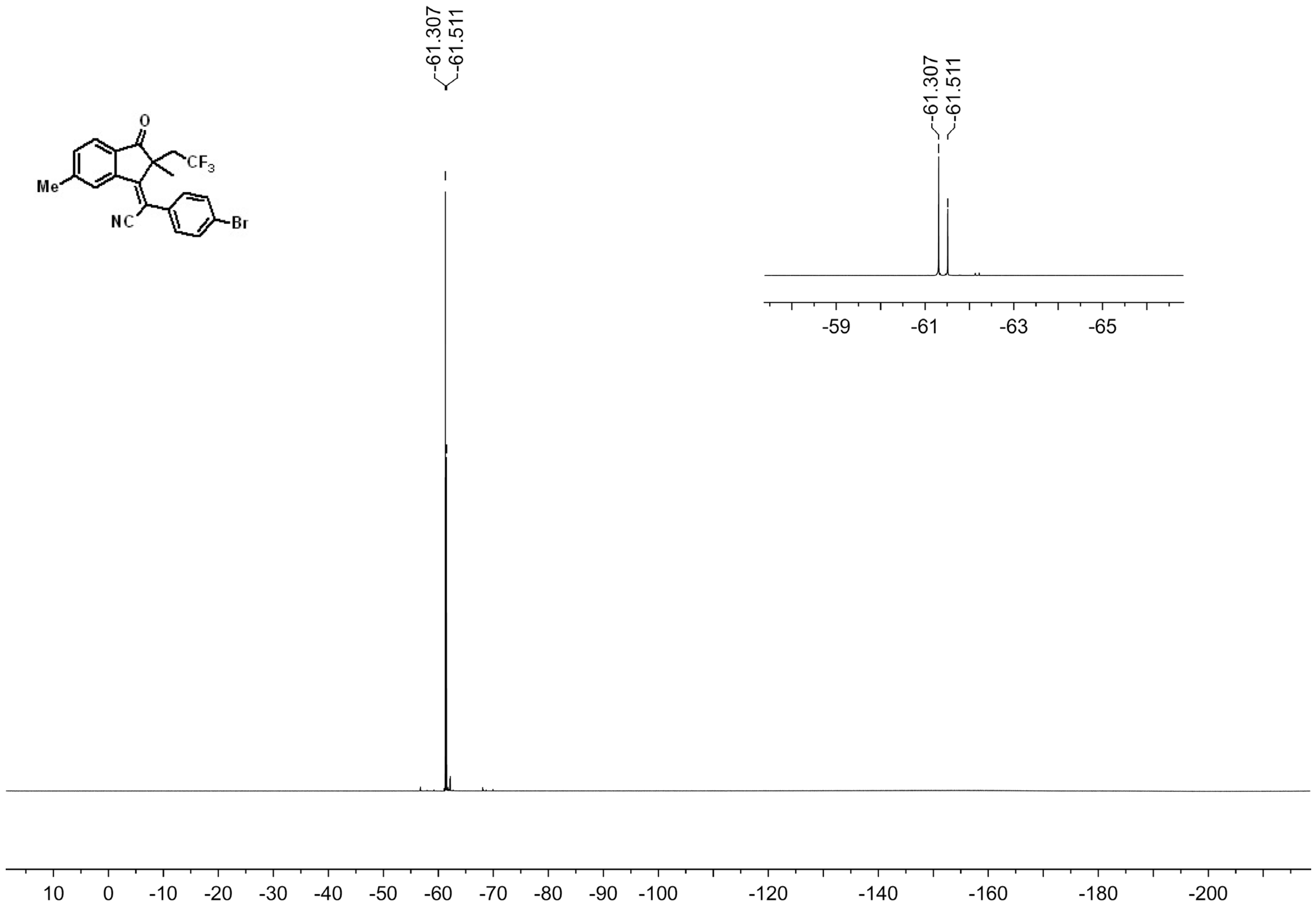

^{19}F NMR Spectrum of Compound **3v**

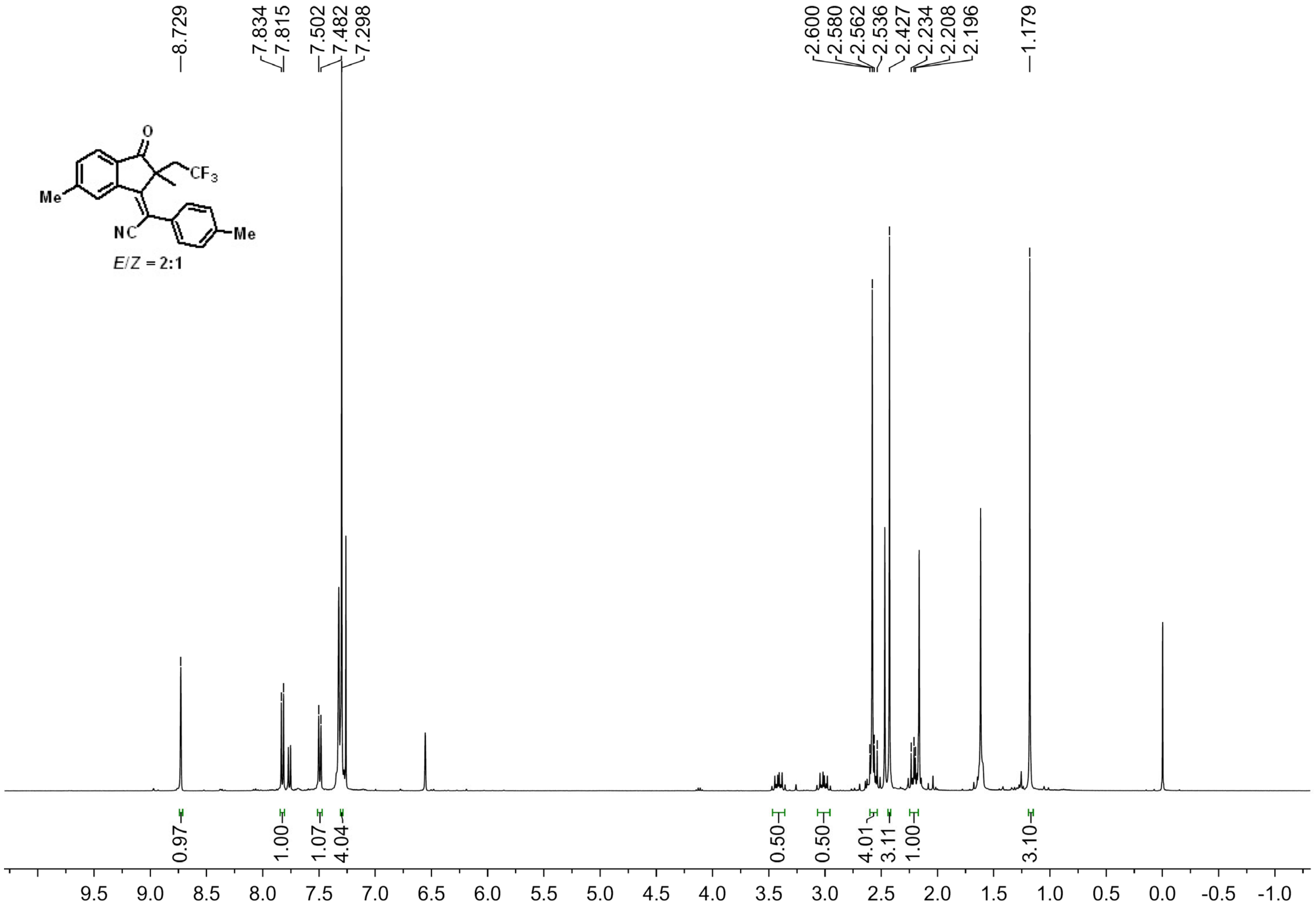

^{1}H NMR Spectrum of Compound **3w**

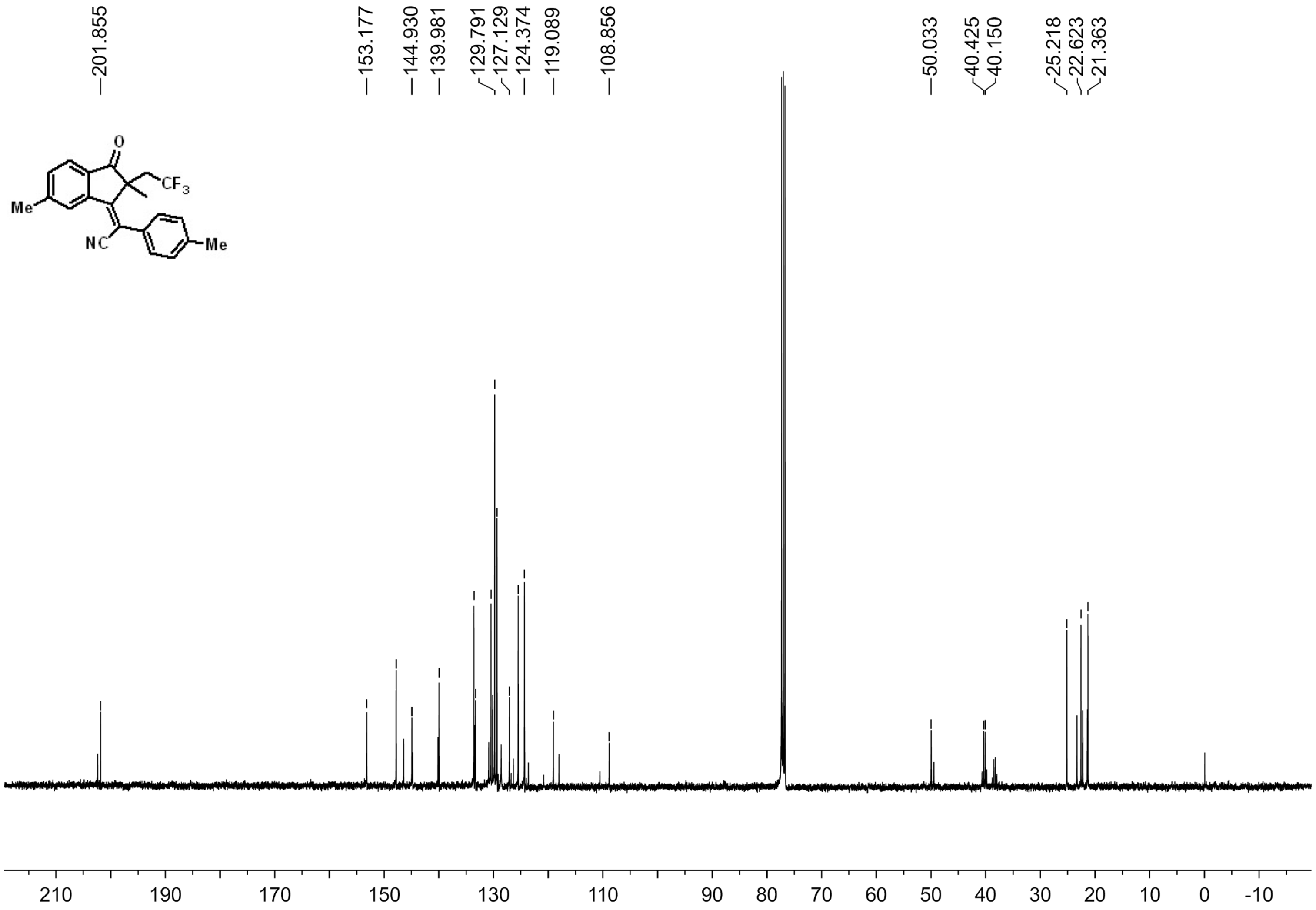

^{13}C NMR Spectrum of Compound **3w**

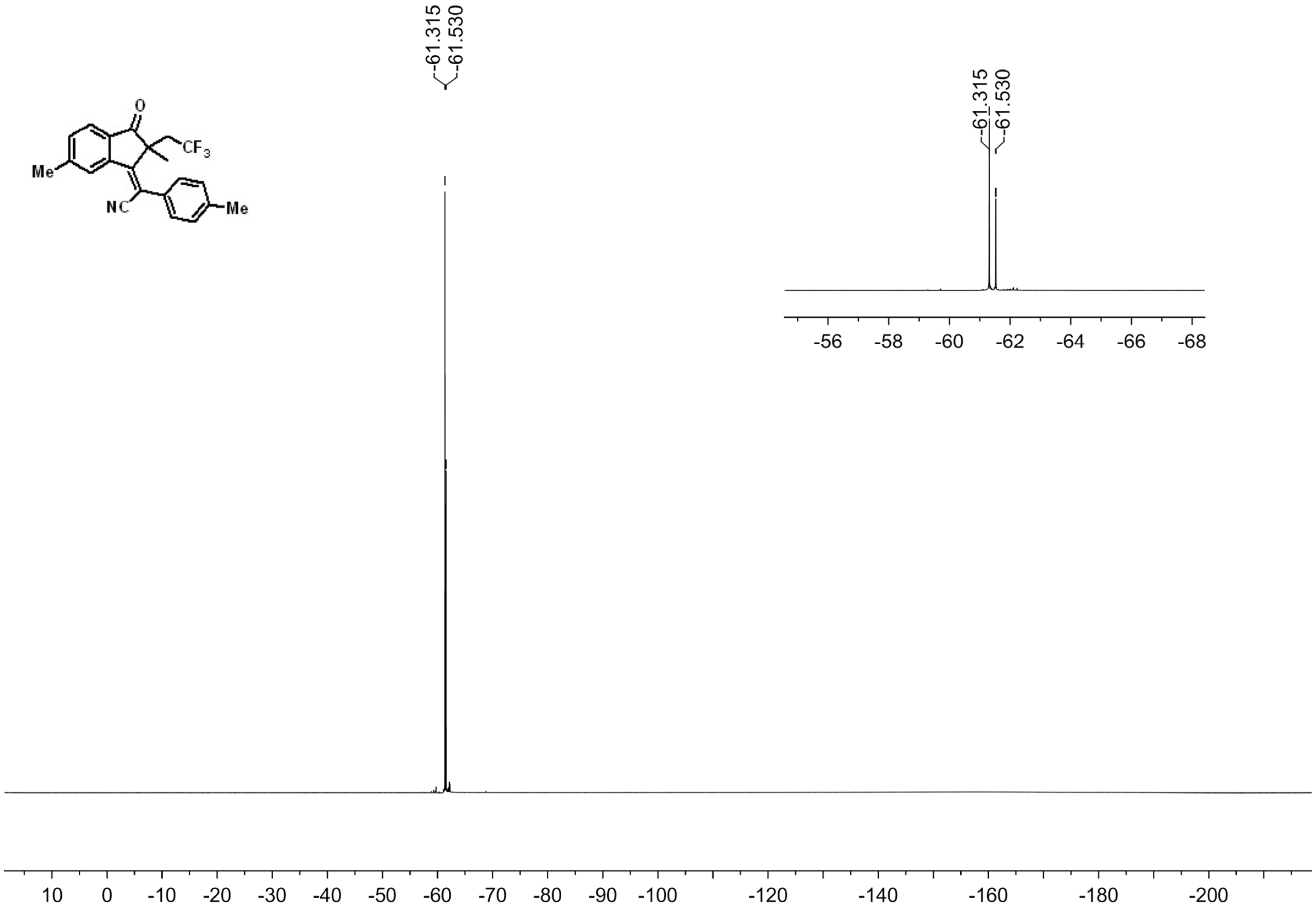

^{19}F NMR Spectrum of Compound **3w**

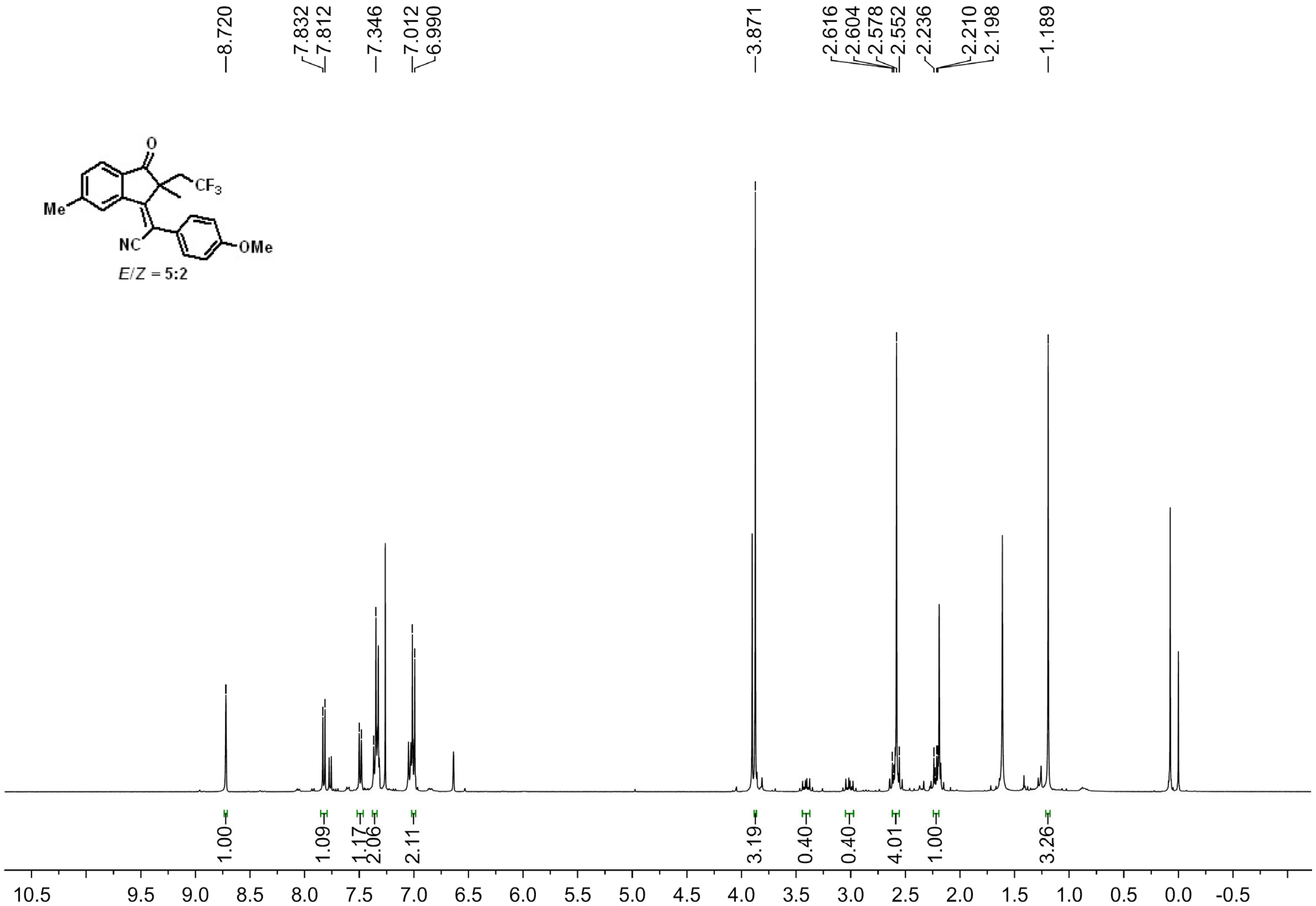

^{1}H NMR Spectrum of Compound **3x**

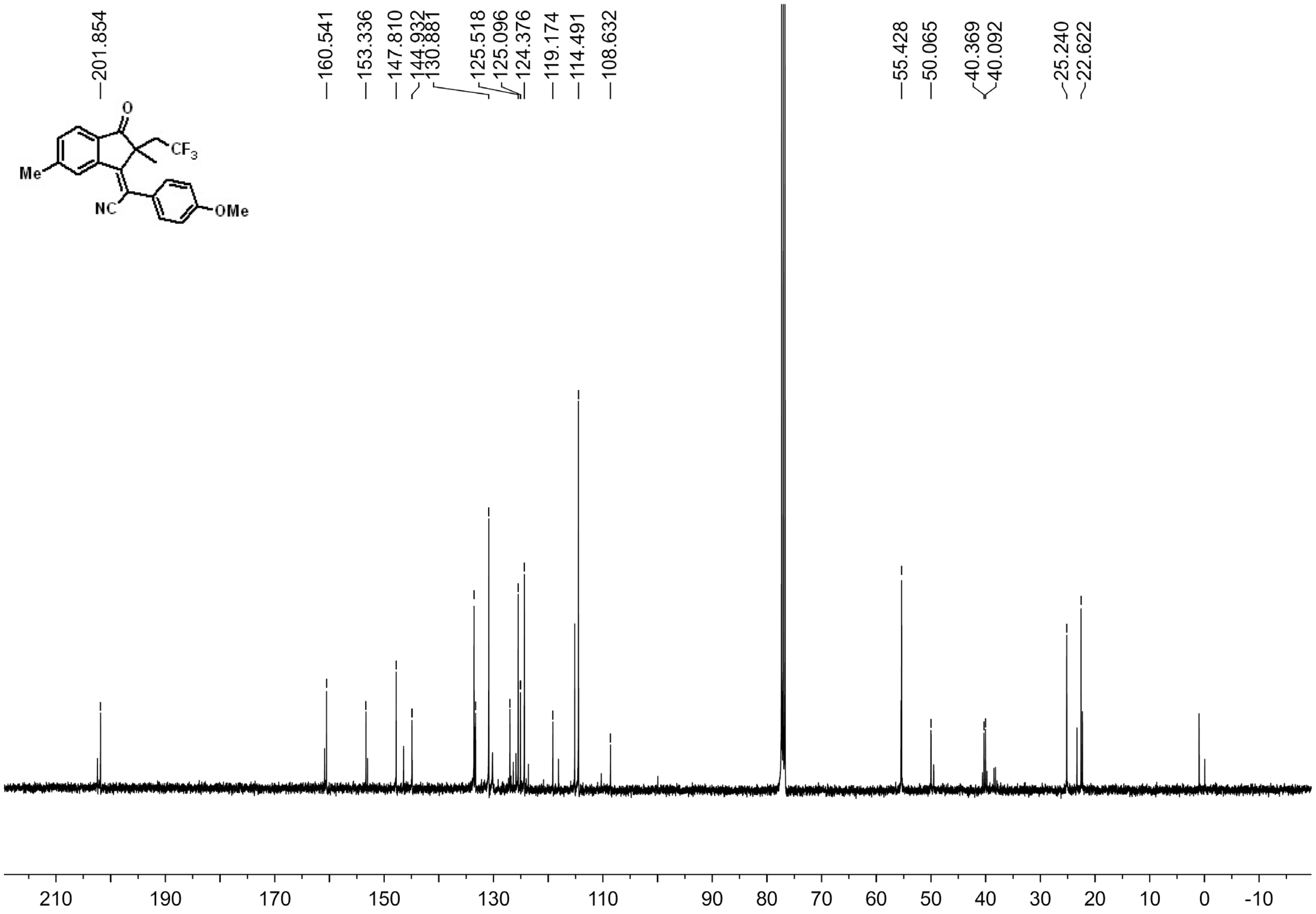

^{13}C NMR Spectrum of Compound **3x**

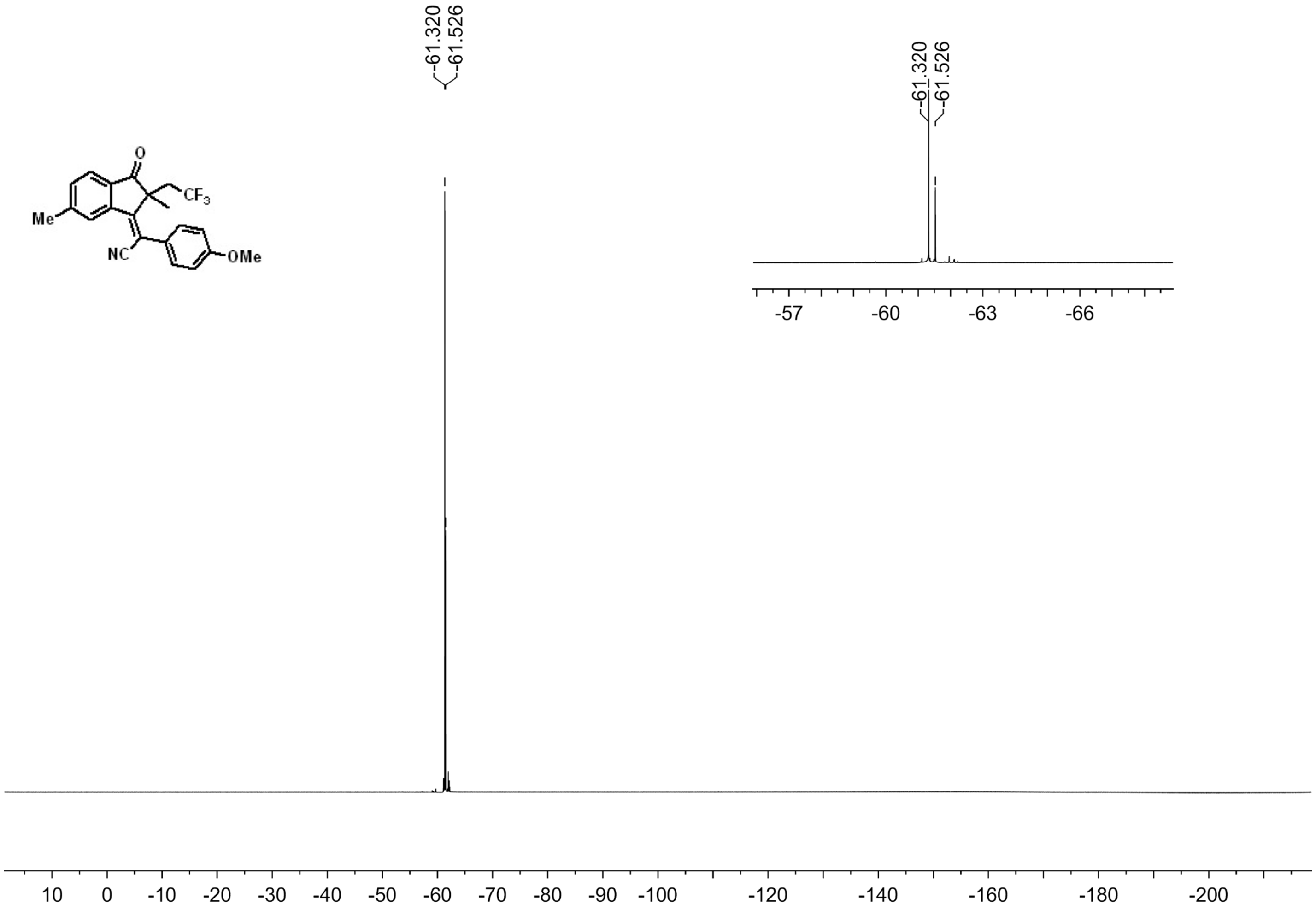

^{19}F NMR Spectrum of Compound **3x**

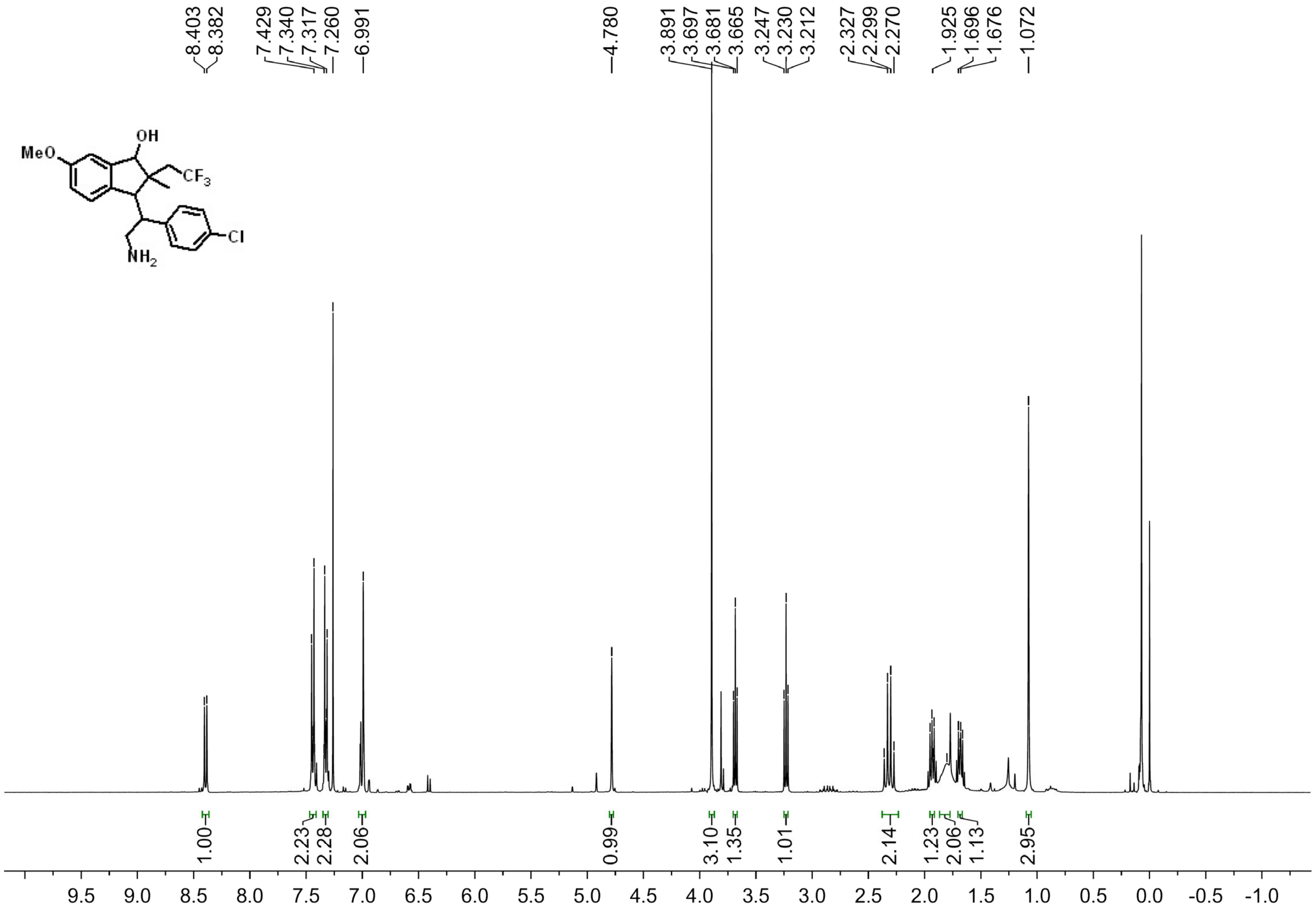

^{1}H NMR Spectrum of Compound **4**

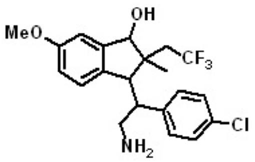

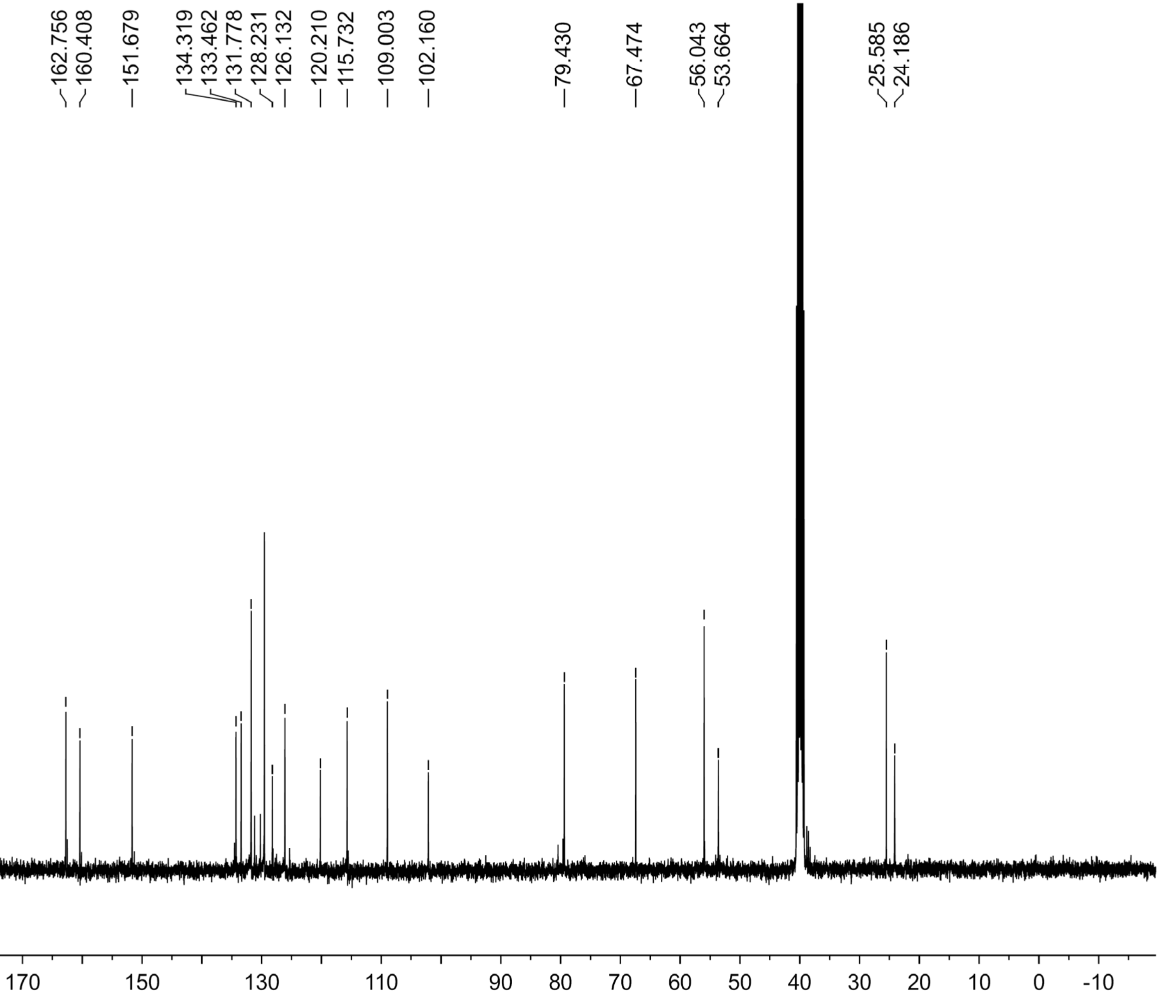

^{13}C NMR Spectrum of Compound **4**

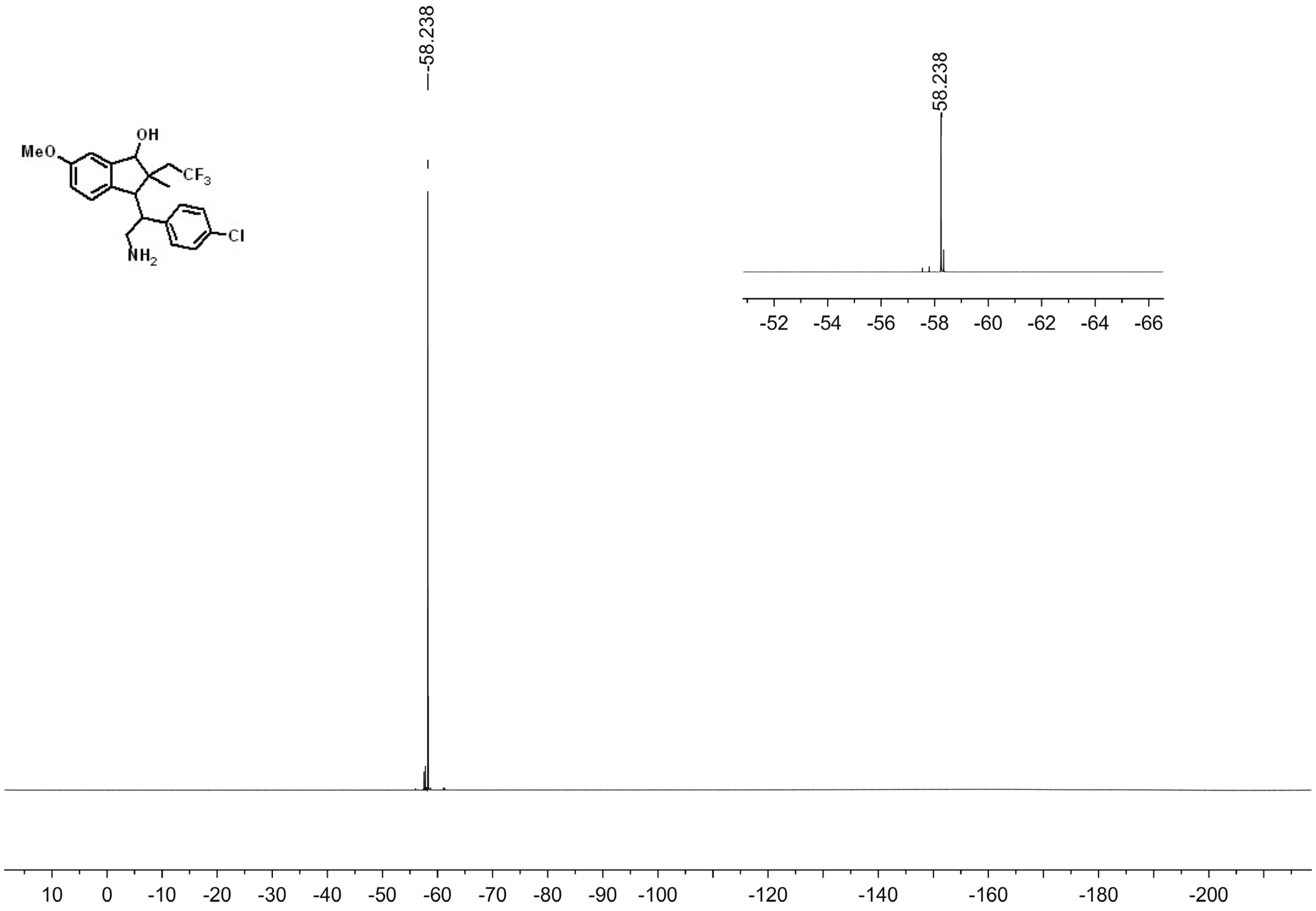

^{19}F NMR Spectrum of Compound **4**
